# AI-augmented ACT-R modeling in real interfaces: automating visual perception, knowledge acquisition, and motor misexecution

**DOI:** 10.3389/fpsyg.2026.1913953

**Published:** 2026-09-16

**Authors:** Amirreza Bagherzadeh, Farnaz Tehranchi

**Affiliations:** 1 Department of Industrial and Manufacturing Engineering, The Pennsylvania State University, University Park, PA, United States; 2 School of Engineering Design and Innovation, The Pennsylvania State University, University Park, PA, United States

**Keywords:** artificial intelligence, cognitive architecture, cognitive simulation, human-computer interaction, instruction parsing, motor errors, visual perception

## Abstract

**Introduction:**

Cognitive models in interactive environments often rely on hard-coded symbolic task descriptions, pre-specified interface objects, and idealized action execution. This paper's primary contribution is EVisiTor, an AI-augmented extension of the VisiTor eyes-and-hands tool for ACT-R that automates two forms of work commonly performed by the modeler: perception of the live interface and acquisition of task knowledge.

**Methods:**

Its visual-perception pathway converts interactive interface objects into ACT-R-compatible visicon features, while its knowledge-acquisition pathway converts human-readable instructions into executable declarative structures. A motor-execution pathway connects ACT-R actions to the operating system and supports probabilistic misexecution with perceptually grounded checking and correction. We use a previously studied Excel procedure as a behavioral case study.

**Results:**

Relative to an idealized perfect-user model, the EVisiTor-enabled model reduced task-level RMSE from 29.19 s to 17.37 s, human-SD-normalized RMSE from 1.24 to 0.66, and mean absolute per-subtask error from 23.1 s to 10.5 s, with smaller absolute error on 12 of 14 subtasks. A spreadsheet knowledge-acquisition analysis showed that prompting the LLMs with the instruction and UIA grounding can produce the intended action and live visual-object names for every scannable target; the file-name input was the sole exception because it has no stable UIA label. A six-task Windows analysis then tested the feasibility of EVisiTor beyond Excel. Broad instructions often resulted in alternative valid methods: exact action agreement ranged from 42.5%–63.6% without UIA and 18.8%–70.6% with UIA, although 41 of 42 generated parses in each condition were functionally sufficient. Detailed guides raised exact agreement to 70.0%–85.0% without UIA and 76.2%–90.0% with UIA, and validated chunks drove the task-independent model through all six tasks in live Windows applications.

**Discussion:**

Together, the results demonstrate EVisiTor's potential for automating interface perception and instruction-based knowledge acquisition for a symbolic cognitive model, while identifying instruction specificity and individual variability as targets for future work.

## 1 Introduction

Cognitive Architectures are applications of cognitive science theories that attempt to simulate cognition and behavior (Anderson, 2007; Newell, 1990). Newell describes cognitive architectures as the way to answer one of the ultimate scientific questions: “How can the human mind occur in the physical universe?” (Newell, 1990). Cognitive architectures are computational frameworks designed to model the mechanisms underlying human cognition and have played an important role in theory-driven human performance modeling (Anderson et al., 2004; Byrne and Pew, 2009; Ritter et al., 2019). Cognitive architectures such as ACT-R and Soar provide computational accounts of human cognition by modeling mechanisms such as memory retrieval, learning, decision-making, goal management, and procedural control (Laird, 2019; Ritter et al., 2019; Sun, 2016). In human-computer interaction and human performance modeling, these architectures are especially valuable because they can generate quantitative predictions about task completion time, learning, transfer, error patterns, and other behavioral measures (Byrne, 2005). However, many cognitive models still operate in simplified, idealized, or instrumented task environments. Instead of visually perceiving the interface as a human user would, the model often receives manually specified symbolic information about interface objects, locations, and states (Ritter et al., 2019).

This creates a perception-action gap in cognitive modeling: the gap between a model's ability to simulate internal cognition and its ability to acquire information, execute actions, and evaluate feedback through channels comparable to sensory inputs available to human users. This limitation is closely related to ecological validity, which concerns whether the modeled task, stimuli, action possibilities, environmental constraints, and feedback loops adequately represent the target context in which the model's predictions are intended to apply (Dhami et al., 2004; Holleman et al., 2020; Subramanian et al., 2023). Without a closed perception-action loop, model predictions may remain accurate only within simplified laboratory conditions or specially prepared simulations, limiting their generalizability to real-world user interfaces.

In this paper, we address this gap by presenting an AI-augmented extension of VisiTor, an eyes-and-hands tool for ACT-R, that allows a cognitive model to operate in real interfaces through a closed perception-action loop. We refer to this extended system as EVisiTor; throughout the paper, VisiTor is referred to the base eyes-and-hands tool and EVisiTor is referred to the AI-augmented extension contributed here. The framework integrates ACT-R with three external capabilities: live visual-environment extraction, instruction-to-declarative-knowledge conversion, and motor execution with probabilistic typing misexecution and correction. We evaluate the framework in a spreadsheet task by comparing an EVisiTor-enabled “realistic” model with an idealized “perfect” user model using human performance data from prior Excel studies.

This study addresses three research questions:

RQ1: Can EVisiTor extend ACT-R to support closed-loop perception and action in an unmodified spreadsheet interface?

RQ2: Can human-readable task instructions be converted into executable ACT-R-compatible declarative structures that support successful model execution similar to human users?

RQ3: Can an EVisiTor-enabled ACT-R model provide a closer approximation of human spreadsheet-task performance than an idealized perfect-user model?

The paper makes three mechanism-level contributions. First, it demonstrates a visual-perception pathway that converts interface objects into ACT-R visicon and refreshes the visicon as the task environment changes (i.e., the insight from RQ1). Second, it introduces a constrained knowledge-acquisition pathway in which an LLM converts human-readable task instructions into modeler-defined declarative structures while ACT-R remains responsible for retrieval, interpretation, production selection, and executing motor actions (i.e., finding of RQ2). Third, it adds a motor-execution pathway that connects ACT-R actions to the interface and supports probabilistic character-level motor misexecutions, error detection, and correction. Together, these pathways move the model from an idealized symbolic task executor toward an agent that perceives and acts in the same software environment as human users, thereby improving the model's accuracy (i.e., the finding of RQ3).

These mechanisms support two methodological advances: they ground perception and the action loop in the real interface rather than a manually simplified representation, and they reduce the burden of manually defining a model by converting task instructions into declarative chunk structures while preserving the interpretability of ACT-R theory.

Prior work has explored several strategies for connecting cognitive architectures to task environments. Some approaches use native perceptual-motor modules with simulated or specially represented interfaces (e.g., ACT-R virtual window or Soar spatial visual system), while others rely on software wrappers, application programming interfaces, or network bridges that expose interface state to the model (Hope et al., 2014; Yuan et al., 2011). Other approaches move closer to real-interface interaction through screen-based perception, image processing, or computer vision, as in SegMan, JSegMan, ACT-CV, and later eyes-and-hands extensions (Halbrügge, 2013; St. Amant and Riedl, 2001; St. Amant et al., 2005; Tehranchi and Ritter, 2017, 2018). Related work has also shown that cognitive models can interact with unmodified spreadsheet interfaces and that error-making behavior can improve model realism, although prior implementations still relied on manually specified or hard-coded components of the task and error process (Tehranchi et al., 2023). In parallel, embodied cognitive architectures such as ACT-R/E have extended similar concerns to physical and robotic environments, where perception and action are constrained by the body and the external world (Trafton et al., 2013).

These approaches reveal an important trade-off. Instrumented and symbolic interfaces are often easier to implement, more reliable, and more transparent for model development, but they can provide the model with task information that human users do not directly possess or require a simplified re-implementation of the environment within specially represented interfaces. Pixel-based, screenshot-based, or embodied approaches can improve ecological fidelity by requiring the model to perceive and act through channels closer to those used by humans, but they introduce perceptual uncertainty, brittleness across interface layouts, action-verification challenges, and greater engineering complexity (Halbrügge, 2013; Kotseruba and Tsotsos, 2020; Tehranchi and Ritter, 2018). The present work is positioned within this trade-off: it uses EVisiTor to connect ACT-R to a real spreadsheet interface while keeping the perceptual, declarative, and motor processes explicit enough to remain interpretable as a cognitive model.

A rapidly growing body of work outside cognitive modeling connects large language models and vision-language models directly to graphical interfaces. GUI and computer-use agents such as CogAgent and SeeClick ground natural-language goals in screen pixels (Cheng et al., 2024; Hong et al., 2024), UFO orchestrates such agents on Windows applications (Zhang et al., 2024), and benchmarks including Mind2Web, WebArena, OSWorld, and Windows Agent Arena measure end-to-end task success on real websites and operating systems (Bonatti et al., 2024; Deng et al., 2023; Xie et al., 2024; Zhou et al., 2023). Screen-parsing models such as OmniParser supply the underlying structured perception (Lu et al., 2024). This literature shares our technical problem of perceiving and acting in unmodified interfaces; however, it does not address our goal: this area of research optimizes task success and automation, whereas cognitive simulation aims to reproduce the human process, including its timing, errors, attention shifts, and individual variability, within an interpretable theory of cognition. EVisiTor therefore uses AI components only for perception and for converting instructions into declarative knowledge, while retrieval, interpretation, production selection, visual search, and motor execution remain ACT-R mechanisms; the model is not an automation agent but an instrument for explaining and predicting user behavior. The two lines of work are complementary: agent benchmarks provide ecologically valid tasks and recorded human demonstrations, and cognitive models provide process-level accounts of the behavior those benchmarks record, a connection we exploit directly in the generalization analysis below.

To evaluate the proposed AI-augmented ACT-R framework, we used a previously developed spreadsheet interaction task as the testbed. It requires users to read instructions, locate cells and interface controls, enter formulas or text, detect mistakes, and correct errors in a real spreadsheet environment, and therefore exercises the perception-action loop that traditional cognitive models often simplify.

The spreadsheet task contains 14 subtasks (see Table 1), including opening and saving a file, calculating frequency, normalization, string length, and typed-character values, inserting rows, entering a name, and inserting the current date. These *subtasks* can be decomposed into smaller perceptual, cognitive, and motor *steps*, such as reading an instruction, locating a target cell, selecting it, typing a formula, pressing Enter, checking the result, and moving to the next target. This structure makes the task useful for evaluating model predictions at both the overall task level and the subtask level.

**Table 1:** The 14 subtasks of the KST.

Subtask number	Subtask description
(1)	Open file
(2)	Save as
(3)	Calculate frequency (B7 to B11)
(4)	Calculate total frequency (B14)
(5)	Calculate normalization (C2 to C6)
(6)	Calculate total normalization (C14)
(7)	Calculate length (D2 To D11)
(8)	Calculate total length (D14)
(9)	Calculate typed characters (E2 To E11)
(10)	Calculate total typed char. (E14)
(11)	Insert two rows
(12)	Type in name (A1)
(13)	Insert current date (A2)
(14)	Save as

These subtasks are performed on both interfaces: Dismal and Excel.

Prior modeling work used this spreadsheet task to examine how cognitive architectures can account for user performance in spreadsheet environments. Paik (2011) and Paik et al. (2015) developed GOMS-like ACT-R models of the Dismal version of the task (see Figure 1), showing that cognitive models could approximate procedural performance across different levels of user expertise. Tehranchi and Ritter (2018) later developed an interactive Dismal model using JSegMan, allowing model actions to be visualized within the actual task environment. This interaction exposed missing actions and motor-positioning assumptions that were not apparent in the earlier non-interactive model, and reduced mean squared error between the completion time of the model and participants from 1745 to 820, an approximately 53% reduction, while *R*^2^ increased only slightly from 0.997 to 0.998.

**Figure 1:**
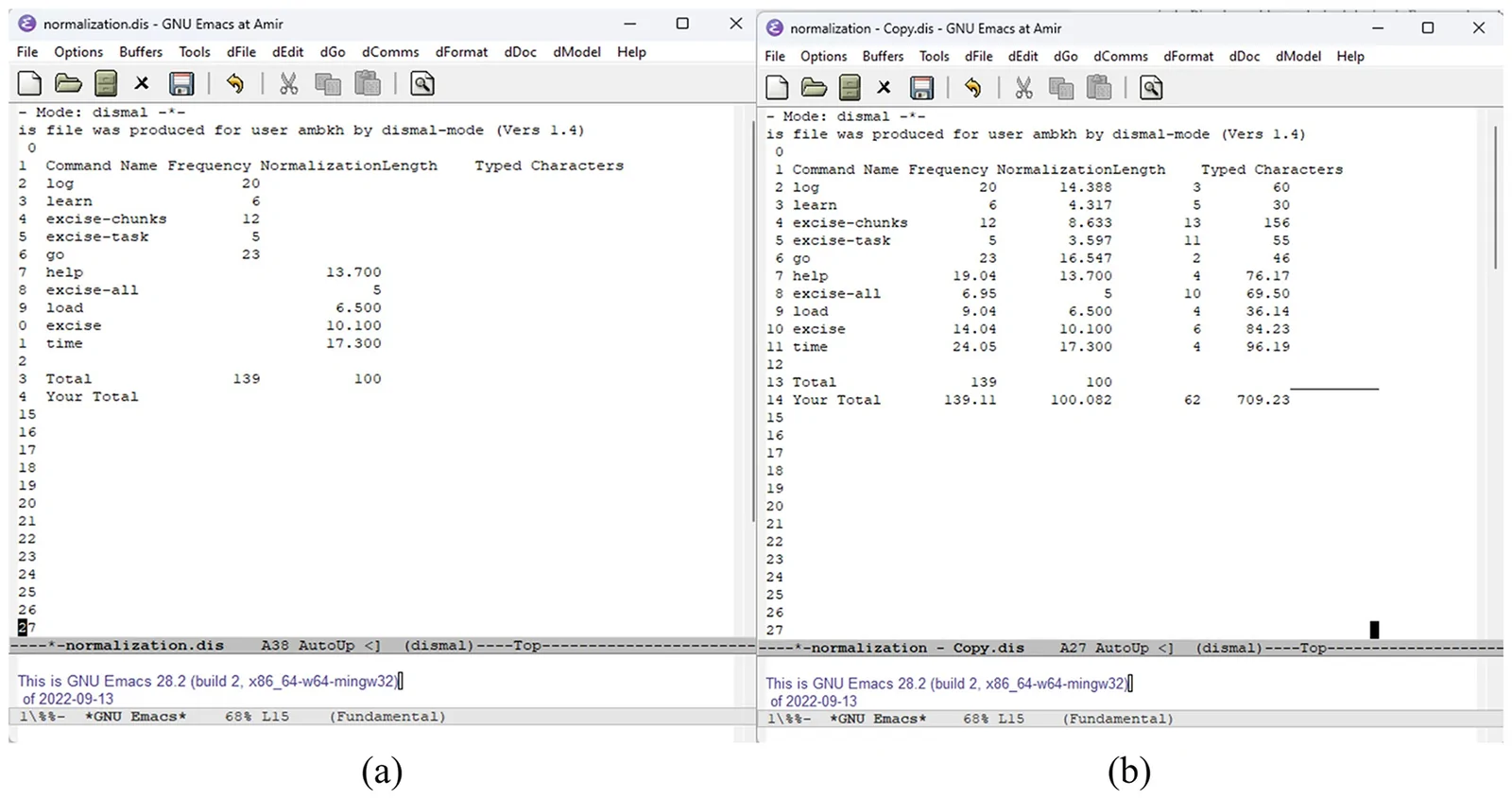
Screenshot of the KST environment containing two Dismal windows. **(a)** The initial state for Dismal is on the left side of the screen, and **(b)** the KST instructions for Dismal are on the right side. Participants use the paper copy of instructions and complete the KST task in the Dismal window (Paik et al., 2015; Tehranchi and Ritter, 2018).

Tehranchi et al. (2023) then replicated the same task structure in Microsoft Excel (see Figure 2) with 23 participants and developed an ACT-R model for the Excel version. Because Excel and Dismal shared the same high-level task goals but differed in interface-specific actions, the comparison demonstrated how cognitive models can be used to evaluate interface efficiency and usability.

**Figure 2:**
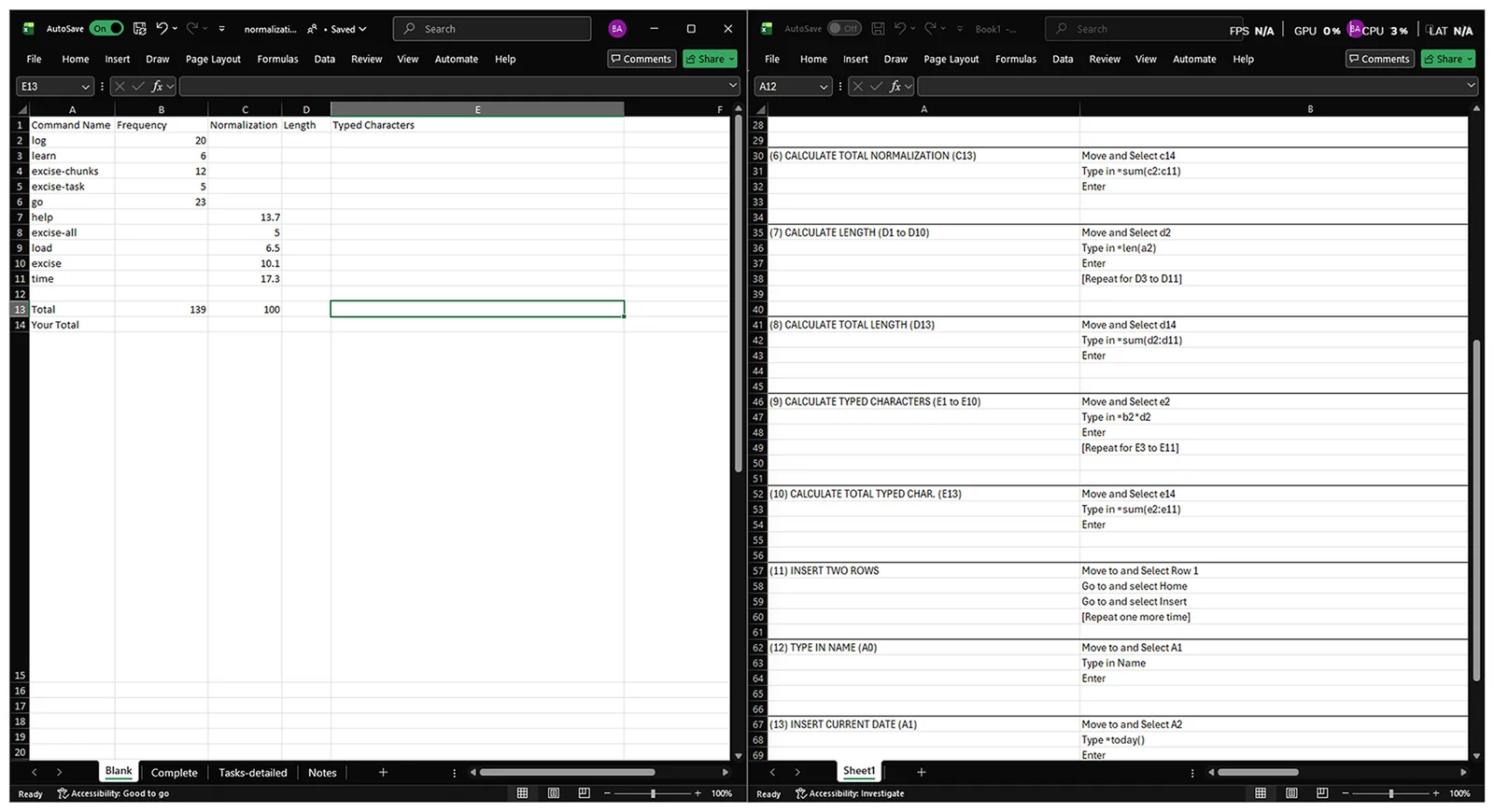
Screenshot of the spreadsheet environment containing two Excel windows. The initial state for Excel is on the left side of the screen, and the KST instructions for Excel are on the right side. Participants use the instructions and complete the KST task in the same way as the Dismal environment (Tehranchi et al., 2023).

The Excel study also revealed an important limitation: participants made and corrected typing errors, while the original model largely represented ideal, error-free performance. A later manually modified model showed that including participant-specific errors could improve correspondence with human behavior, but this approach was not scalable because the errors were hardcoded. The present work builds on this history by using EVisiTor to connect ACT-R to the real Excel interface, support live visual perception, generate task knowledge from human-readable instructions, and model probabilistic motor misexecutions and corrections.

To test whether behavioral error could improve model fit, Tehranchi et al. manually hardcoded one participant's specific typing errors into the model. This modified model produced a higher correlation with the participant's observed completion times, although the mean squared error was slightly higher. The result suggested that including error-making and error-correction behavior could make cognitive models more realistic, even if the exact implementation required further refinement. Their work demonstrated the feasibility of using cognitive models to evaluate interface usability and emphasized the importance of accounting for errors when predicting subtask completion times.

Building on these findings, we extend the previous work in two main ways. First, rather than manually hardcoding individual participant errors, EVisiTor generates and corrects typing errors from probabilistic data on participants' observed mistakes, so error-making becomes a generalizable component of the model rather than a manually specified sequence for a single participant.

Second, we address a limitation the previous work did not explore: the model's ability to perceive and represent the interface environment in real time. Prior models depended on predefined task representations and manually specified assumptions about the interface state, whereas EVisiTor supplies real-time environment representation through UIA and VLM, allowing the model to obtain information from the actual user interface rather than a static or pre-coded description of the task environment.

## 2 Materials and methods

### 2.1 Participants and spreadsheet-task dataset

To develop a model that can better capture the nuances of human performance, we first reanalyzed the data previously collected by Tehranchi (2020). The dataset included 23 participants (18 male, 4 female, and 1 participant whose gender was not specified in the main demographic table), all undergraduate or graduate students at The Pennsylvania State University. Participants had an average age of 25.13 years (SD = 5.87) and varied in Microsoft Excel expertise (4 basic, 17 intermediate, 1 advanced, and 1 who reported never using Excel). None had prior knowledge of the task or its specific operations. Keystrokes, mouse clicks, mouse movements, and eye movements were recorded using GazeTracker; however, only one data stream was recorded at a time, so cursor movement periods do not contain simultaneous eye-tracking data. Video recordings were available for eight participants and were used for exploratory extraction of gaze patterns. Twenty-one participants completed the session; the summed mean completion time across the 14 subtask windows was 715.4 s. The 715.4 s figure is the sum of the 14 subtask-window means; time spent between subtask windows (for example, transitions between finishing one instruction and attending to the next) is not assigned to any window, so this windowed total is the appropriate reference for the models, which are scored over the same 14 windows.

Because different analyses draw on different subsets of the recorded data, due to data collection issues from Tehranchi (2020) data, Table 2 summarizes the participant and observation flow for every analysis reported in this paper.

**Table 2:** Participant and observation flow across analyses.

Analysis	Participants	Observations	Exclusion reason
Enrollment Tehranchi (2020)	23	–	–
Completion time (Figure 6; Table 4; Supplementary Tables S17–S20)	21	21 × 14 subtask windows	two participants did not complete the session
Typing and correction (Supplementary Appendix B; Supplementary Tables S1, S2)	19	639 formula entries	four keystroke logs empty (logger failure); per-step *N* varies 13–19 (partial logs retained)
Formula error-rate calibration	19	6,236 formula content keystrokes	same as typing analysis
Word error-rate calibration	16	669 word content keystrokes	three further logs contained no word-entry segments
Gaze analysis (exploratory)	8	eight gaze-overlay videos	gaze overlay unavailable or unusable for the remainder

Each row gives the number of participants contributing to an analysis, the observation unit, and the reason for any exclusion.

An exploratory eye-tracking analysis of the gaze-overlay videos (*n* = 8 usable videos) was used to motivate the model's assumptions about attention shifts during formula entry, word typing, and cell search. Because these analyses are qualitative and exploratory, the full analysis, including the gaze figures (Supplementary Figures S1–S3), is reported in Supplementary Appendix A.

### 2.2 Typing analysis

Based on the keypress data that we have, we broke down the keypresses into per-step entries. Formula typing is segmented from the first “=” key through the next Enter. Keystroke logs were recorded for all 23 sessions, but four logs contained no keyboard events due to a logger failure, leaving *N* = 19 participants for all keystroke analyses in this section, including those reported in Supplementary Appendix B. Because some logs ended before the final steps, the number of contributing participants varies by step, from 19 (Steps 3–7) to 13 (Step 13); partial step sequences were retained rather than excluded, so a step can contain fewer entries than the number of participants times the expected entries per participant (for example, Step 9 comprises 174 entries from 18 participants, one of whom contributed 4 of the 10 expected entries before their log ended).

For model calibration, we also measured typing-error rates from the human keystroke logs as correction episodes per content keystroke. The measured rates were 3.35% for formula entry (SD = 1.72%, 19 subjects) and 6.28% for word entry (pooled; per-subject mean 6.66%, SD = 7.21%, and 16 subjects). These values were supplied to the model as 0.034 and 0.063, respectively, rather than fitted to completion-time outcomes.

Detailed keystroke metrics (including per-step formula-entry segment counts, correction and backspace definitions and values (Supplementary Table S1), within-task transition intervals (Supplementary Table S2), and a character-level correction summary) are reported in Supplementary Appendix B.

We used paired Wilcoxon tests to compare task-level mean IKI and error-making in a pairwise manner, with Holm correction for multiple pairwise comparisons. Mean IKI differed across the formula-entry tasks (Friedman χ^2^ = 38.8, *p* < 0.001, 13 complete-case participants): typing was slower in the encoding-heavy tasks, and seven pairwise contrasts remained significant after Holm correction (e.g., Step 3 vs. Step 7: mean IKI 0.83 s vs. 0.58 s, Holm *p* = 0.002). Error-making did not differ across tasks (correction episodes per entry: Friedman *p* = 0.079; no pairwise contrast significant after Holm correction).

### 2.3 ACT-R modeling and EVisiTor architecture

This section describes the modeling architecture used in the spreadsheet task and the EVisiTor extensions that allow ACT-R to operate in an unmodified Excel interface. We use ACT-R 7 coupled with EVisiTor so that the model can perceive the live spreadsheet interface, acquire task-relevant knowledge from human-readable instructions, perform visual search, and execute keyboard and mouse actions in the external environment. The central methodological idea is that the model should not be given direct access to the location of a desired cell, button, or typed response. Instead, it should operate through the same broad loop used by human participants: read the instruction, shift attention to relevant information, search the visual scene, locate the intended object, act, detect possible errors, and correct them. The comparison with the perfect-user baseline therefore evaluates the added value of modeling perception, action, and error correction in the real interface rather than only executing an idealized task script. Implementation-level corrections to the earlier baseline (Excel model developed by Tehranchi et al., 2023) are reported in Supplementary Appendix C.

### 2.4 ACT-R modeling framework

The model follows the ACT-R 7 cognitive architecture. In ACT-R, task knowledge is represented as declarative chunks, and behavior is controlled by production rules that match the model's current state and update buffers. In this model, the goal buffer represents the current task state; the retrieval buffer retrieves task and action knowledge from declarative memory; the visual-location and visual buffers support visual search and attention to interface objects; the imaginal buffer maintains temporary task and spatial information; and the manual buffer issues keyboard and mouse actions.

These buffers are used in a perception-action loop rather than as a direct script for manipulating Excel. The model retrieves the next instruction, interprets it through declarative task knowledge, requests visual information, shifts attention to the relevant interface object, evaluates the attended object, and then executes the corresponding motor action. This means that spreadsheet actions emerge from ACT-R mechanisms of retrieval, attention, visual search, and manual execution rather than from pre-specified calls that directly edit the spreadsheet.

EVisiTor supplies the bridge between these ACT-R mechanisms and the real Excel interface. It converts visible interface elements into ACT-R-compatible visual features, supports instruction-to-declarative-knowledge conversion, and routes ACT-R motor actions to the operating system. ACT-R controls goal management, retrieval, production selection, visual attention, and motor execution, while EVisiTor makes those mechanisms operate in the same interface environment used by human participants. Detailed chunk definitions (Supplementary Table S3), production-rule logic, and implementation details are provided in Supplementary Appendix C.

### 2.5 EVisiTor extension 1: visual perception

The first EVisiTor extension supports visual perception. Standard ACT-R vision can search and attend to items in a visicon, but in many models, the visicon is manually specified or tied to a simplified experimental display. This is limiting for real software such as Excel, where interface objects appear, disappear, move, and change state after clicks or keypresses. EVisiTor addresses this by converting the current computer interface into ACT-R-compatible visual objects that can be searched and attended by the model.

In the spreadsheet task, EVisiTor combines screenshot-based template matching with structured interface extraction. The resulting visual description allows ACT-R to search for cells, row and column headers, buttons, tabs, and other visible objects using ordinary visual-location requests. This preserves the cognitive role of visual search: the model must locate the relevant object in the currently perceived scene instead of receiving its position in advance.

The visual scene is refreshed after interface-changing actions, allowing the model to search the current display rather than a static representation created before the task began. EVisiTor also externalizes ACT-R attention by making attention shifts observable on the screen, which supports validation against human gaze behavior. Figure 3 summarizes the visual-perception pathway; implementation-level fields and conversion details are reported in Supplementary Appendix C.

**Figure 3:**
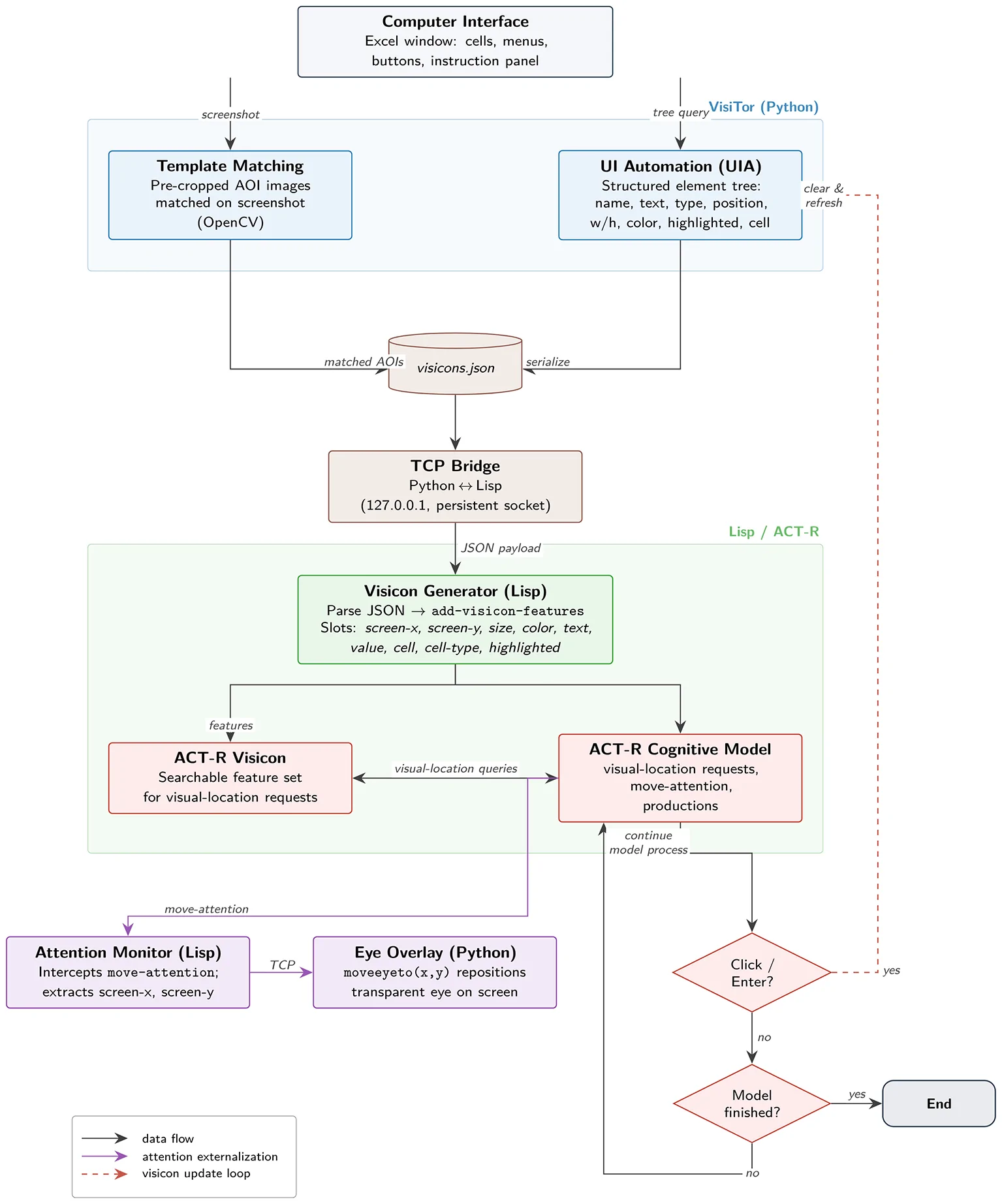
The EVisiTor visual-perception pipeline.

UI Automation raw output exposes metadata that human users do not necessarily have direct visual access to. EVisiTor filters the UIA tree of the visible task windows and converts only the retained interface elements into visicon features. Each feature carries only: its on-screen location and size (from the element bounding rectangle), its visible label or value text, a coarse item type (cell, row or column header, button, tab, menu item, list item, or text), its average display color mapped to the nearest ACT-R color name (colors are not used for the analysis but it is important for future studies that want to incorporate bottom-up visual search), and, for cells, highlight status. Table 3 classifies the interface information by how a human user could obtain it and states what the model receives.

**Table 3:** Interface information available to human users and to the model.

Information class	Examples	Human access	Model access
Visible on screen	Cell contents, button and tab labels, menu items, dialog fields	Read directly	Received (label text, location, size, and color)
Visually inferable	Which cell is highlighted; row/column of a cell; element type (button vs. cell)	Inferred from appearance	Received as pre-classified attributes (highlight status, row/column role, and item type)
Accessibility-derived metadata	UIA element names identical to the visible label; control types	Not read directly (inferred visually)	Received; used as the searchable label of the visible object
Hidden interface state	UIA tree hierarchy, automation IDs, control patterns, off-screen elements, application internals (e.g., stored formulas)	Not available	Withheld (discarded during conversion)
Target locations for the current instruction	Found by visual search	Found by visual search	Not provided; the model must search the visicon

“Received” means the information is included in the visicon features; “withheld” means EVisiTor discards it.

Two safeguards prevent an informational advantage over human participants. First, the visicon contains only elements of the currently displayed windows, that is, the model cannot query hidden state, off-screen content, application internals, or the UIA tree structure itself (parent-child relations, control patterns, and automation IDs are discarded). Second, and more importantly, receiving a labeled feature is not the same as knowing where to act: the model is never handed the location of the target of the current instruction. It must generate visual-location requests, search the scene, attend candidate objects, and evaluate them, the same search process the limited eye-tracking data show in participants. The UIA label plays the role of the word a user reads once the object is fixated; acknowledged residual differences (perfectly legible labels, no perceptual confusability between similar glyphs) are noted as limitations.

### 2.6 EVisiTor extension 2: knowledge acquisition

The second EVisiTor extension supports knowledge acquisition. ACT-R models normally require modelers to manually define declarative memory chunks, but users often read task instructions during task performance. A model that begins with every instruction already encoded as internal task knowledge has an informational advantage and ignores part of the user's cognitive work. EVisiTor reduces this problem by translating human-readable task instructions into declarative structures that ACT-R can retrieve and use.

This translation is intentionally constrained. EVisiTor does not ask a language model to write the ACT-R model or create new production rules. Instead, the modeler defines the representational schema and action vocabulary, and the language model converts the task instructions into structures that fit that schema. Thus, AI supports the acquisition of task knowledge in a predefined format, while ACT-R remains responsible for retrieval, interpretation, production selection, visual attention, and action execution.

For the spreadsheet task, instructions are represented as ordered action-item structures such as going to a cell, selecting it, typing a formula, or pressing Enter. The model does not simply execute this list directly. It attends to the instruction region, retrieves the current task structure, maps the human-readable action to an internal action meaning, searches for the relevant object, and then acts through the manual buffer. Figure 4 illustrates this separation between AI-assisted knowledge conversion and ACT-R cognitive control; prompt, endpoint, and schema details are reported in Supplementary Appendices D–E (Supplementary Table S4).

**Figure 4:**
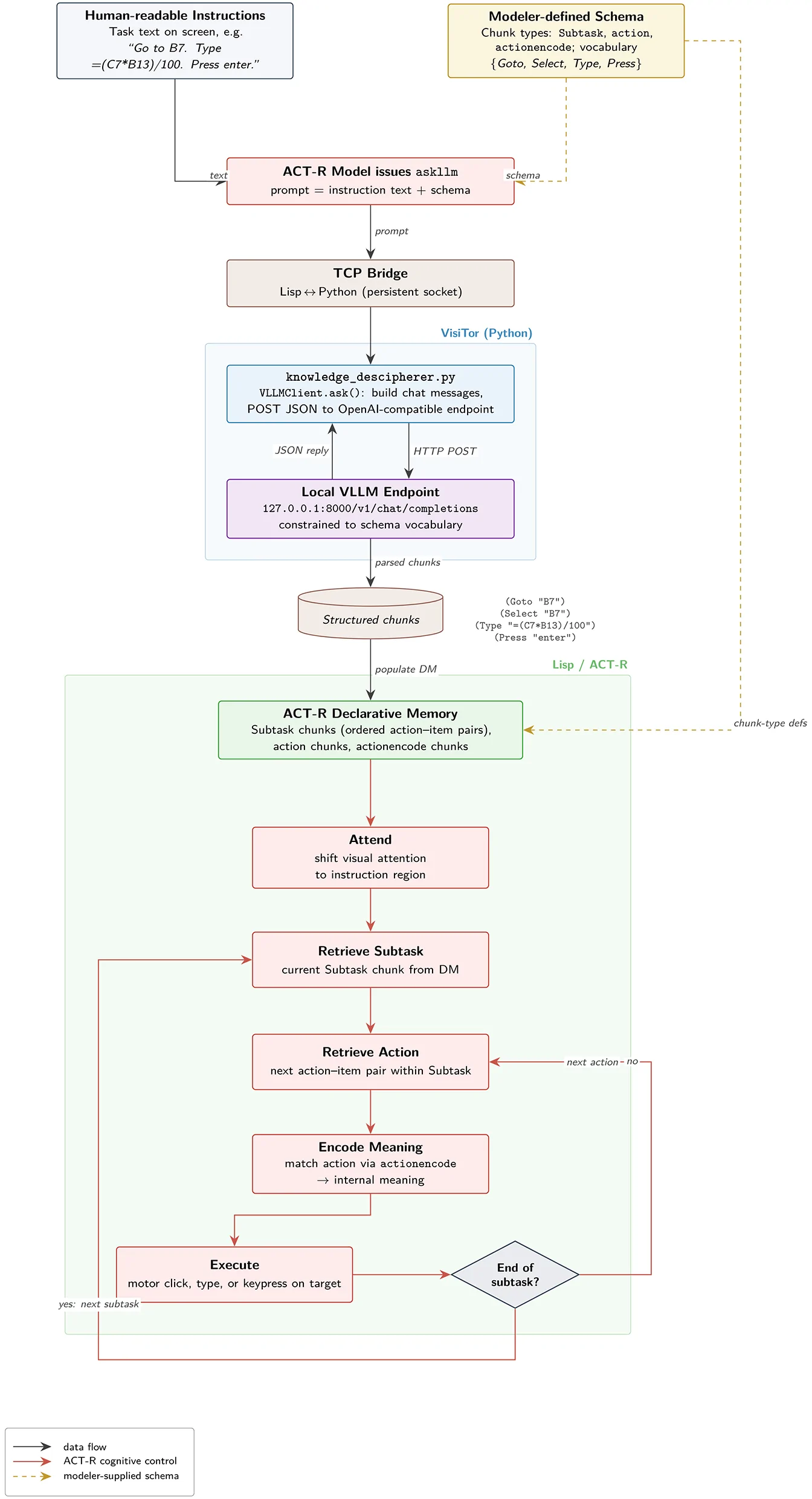
Flowchart of the knowledge acquisition extension. Modeler-supplied schema and action vocabulary constrain the LLM output, while ACT-R retrieves, interprets, and executes the resulting declarative task structures through its cognitive-control cycle.

### 2.7 EVisiTor extension 3: motor execution and error production

The third EVisiTor extension connects ACT-R motor execution to the real operating system and supports errorful execution. ACT-R can issue manual actions through its manual buffer, but those actions do not affect an external application. EVisiTor routes ACT-R keyboard and mouse actions to Excel so that the model's choices change the interface state that a human user would use.

This bridge also makes it possible to distinguish the intended key from the key actually delivered to the interface. In the realistic model, character-level typing slips are sampled as motor misexecutions, while command keys such as Enter or Backspace are treated separately. The delivered character is what appears on the screen: EVisiTor exposes it to the model the same way the interface exposes it to a human user, as visible text at the typing location. To detect a slip, the model shifts visual attention to that location, encodes the typed character, and compares what it saw with the character it intended to type; a mismatch sends the model into a correction state before retyping the intended character.

This design treats typing mistakes as motor-execution errors rather than as failures of task knowledge or as cognitive errors. Higher-level mistakes would need to be modeled in ACT-R's cognitive control process, but character-level substitutions that are due to motor misexecution can be represented through the EVisiTor motor pathway. Figure 5 summarizes the motor-execution and correction loop; TCP commands, JSON messages, protected-key rules, and implementation details are reported in Supplementary Appendix C.

**Figure 5:**
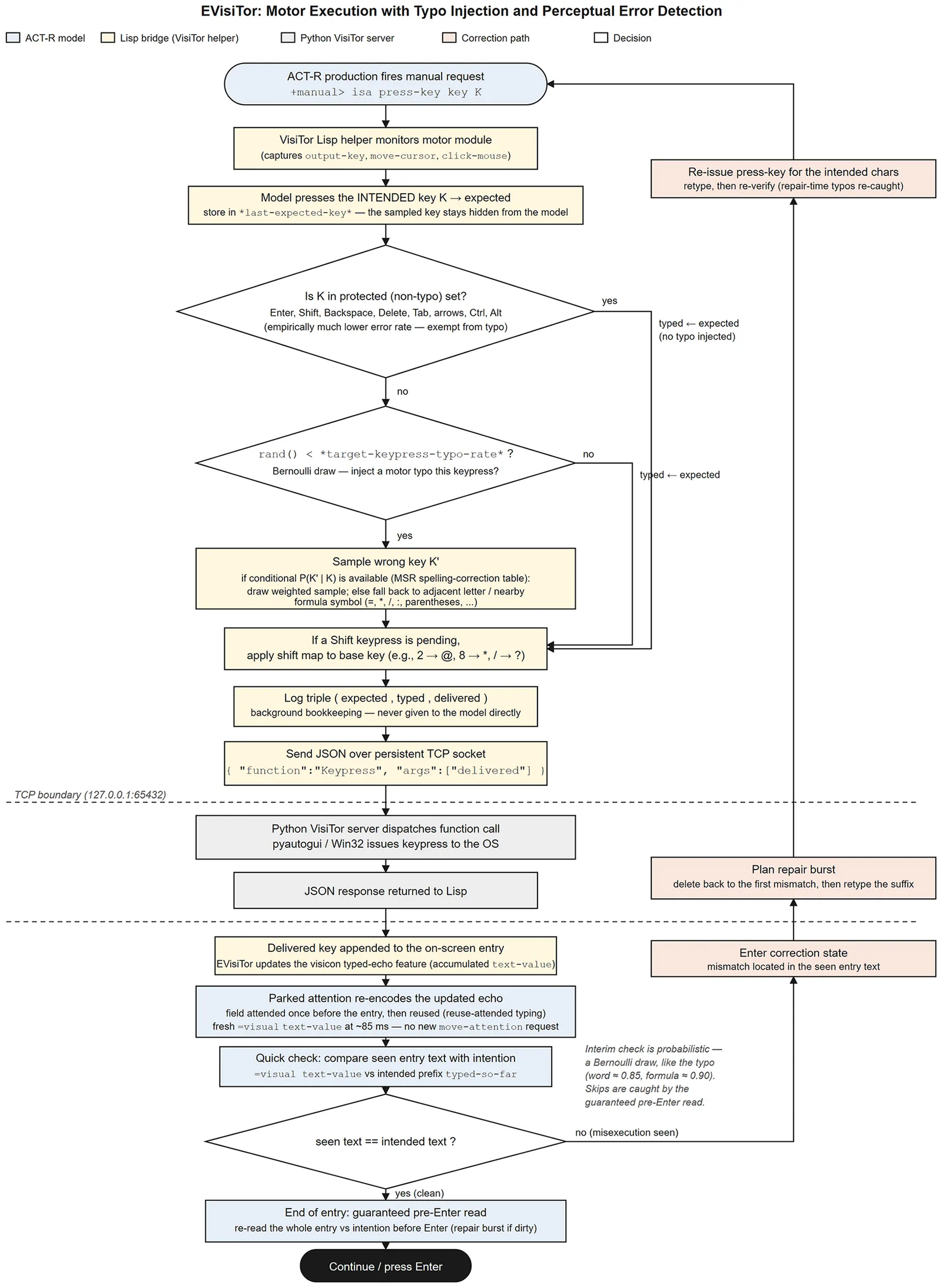
EVisiTor motor-execution pathway with typo injection and perceptual error detection. ACT-R issues manual requests, EVisiTor samples possible character-level misexecution and delivers the resulting keypress to the interface, where the typed character becomes visible at the typing location. The model detects a misexecution by shifting visual attention to the typed text, encoding the character on the screen, and comparing it with its intention; a mismatch puts the model into a correction state. The intended/typed/delivered triple is logged in the background for analysis and is never given to the model except through vision.

Error detection is perceptual. Before the first keypress, the model attends the editable field once. Each delivered key updates that same visicon feature's accumulated TEXT-VALUE. ACT-R's vision module treats a change to the currently attended object as a new encoding: it becomes busy immediately and, after the standard 85 ms visual-attention latency, places the fresh percept in the visual buffer. This re-encoding runs in parallel with motor activity. Checking productions therefore do not issue a new visual-location or move-attention request; they wait for vision and the hand to be free, then match the visible accumulated entry against the intended characters on the production left-hand side. For common word characters, the model checks for errors with probability 0.85, and the final word character is always checked. Formula characters are typed in tokens; after each completed nonfinal token, the model looks back to the formula source, returns to the target, and checks the accumulated entry with probability 0.90. The final formula token is covered by the entry-level check. Before Enter, every completed entry receives a guaranteed whole-entry comparison. If the visual buffer was harvested, the model refreshes the same stored typing location before comparing.

### 2.8 Spreadsheet task model

The Excel model performs the 14-Subtask spreadsheet normalization procedure used by Tehranchi et al. (2023). At a high level, each Subtask follows the same cognitive cycle: retrieve the current subtask instruction, attend to the relevant instruction region, interpret the next action, search for the target object in the current interface, execute the required motor action, and advance to the next action, subtask, or task. This cycle allows the model to represent spreadsheet work as instruction-guided perception and action rather than just a direct spreadsheet manipulation from memory.

Visual search differs between cell and non-cell targets. For spreadsheet cells, the model uses the structure of the sheet by locating column and row information and then attending to the target cell at their intersection. For non-cell interface objects, such as menus or buttons, the model uses remembered locations when available and otherwise searches the visible interface. This distinction is important because it reflects the way spreadsheet structure constrains visual search while still requiring the model to perceive the actual interface state.

Typing is also modeled at a process level. Ordinary words are typed character by character, whereas formulas are treated as structured input that may require repeated attention shifts between the instruction source and the target cell. The realistic model includes probabilistic typing misexecutions, perceptual checking (shifting attention to the typed text and encoding it before deciding whether a correction is needed), delayed detection, and correction, while the perfect baseline suppresses these errorful-execution mechanisms.

#### 2.8.1 Formal definition of the ablation conditions

Across all eight conditions, the core setup remained identical, utilizing the same ACT-R architecture, 14-subtask declarative specification, interface bridge, Visicons, and architectural motor timings. The conditions differed only through a complete 2 × 2 × 2 factorial design that crossed three explicitly controlled mechanisms: V (visual search for target locations), B (batched formula typing, where the model looks back at instructions between typed token batches), and C (an integrated error-and-correction package). Group C deliberately pairs probabilistic, character-level motor errors with corresponding perceptual checking and repair mechanisms. This coupling is essential; generating errors without an active repair mechanism would leave the model unable to resolve Excel's persistent error pop-ups, stalling task completion. Therefore, C should be understood as a complete error-and-recovery process, rather than just a baseline checking cost added to error-free typing.

1. Factor *V* (visual search). *V* = 1 enables the local/global visual-search mechanism for cells and interface objects; *V* = 0 disables it and supplies target locations through the same idealized direct or recalled-location access used by the perfect baseline. This factor isolates the time and stochasticity introduced by locating and attending targets in the perceived interface.

2. Factor *B* (batched formula typing). *B* = 1 enables formula look-backs, so the model returns to the formula instruction between typed token batches; *B* = 0 disables them, so formulas are typed from memory after their initial encoding. This factor isolates the formula-encoding and look-back strategy.

3. Factor *C* (errors and correction). *C* = 1 sets the formula content-key typo probability to the human-measured formula rate of 0.034 and the word content-key typo probability to the human-measured word rate of 0.063, and enables the perceptual checking chain: (a) probabilistic checks of the typed text (word characters are checked with probability 0.85 each; formula tokens are checked with probability 0.90 at each formula look-back, so an in-progress formula check exists only when typing is batched (*B* = 1). When a formula is typed without batching (*B* = 0) no per-keystroke or per-token check probability applies during typing, and the formula is typed straight through), (b) delayed word-error realization, (c) the pre-Enter read-back of every completed entry, and (d) the repair productions. Each check shifts visual attention to the typed text, encodes the character on the screen, and compares it with the intended character before typing continues. *C* = 0 sets both typo probabilities to 0 and disables every checking and repair mechanism.

4. Cells. The eight factor combinations define the eight ablation conditions, reported in the canonical order 000, 100, 010, 001, 110, 101, 011, 111 (*VBC*). Cell 000 is the perfect lower-bound baseline of Tehranchi et al. (2023); cell 111 is the full realistic model; the single-mechanism cells 100, 010, and 001 correspond to the visual-search-only, batched-formula-typing, and correction-checking conditions.

5. Provenance. One archived 142-element seed list was applied in the same order to every cell, seeding ACT-R's random module. The per-cell run count of 142 was set a priori by a power analysis (Ritter et al., 2011): detecting a medium effect size (Cohen's *d* = 0.5) at α = 0.05 with a statistical power of 0.99 (1 − β) requires 142 runs per condition.

Detailed chunk types, production-rule sequences, visual-search logic, typing pathways, logging streams, and baseline-model fixes are provided in Supplementary Appendix C; the ACT-R parameter set, typing and checking parameters, seed list, and batch-run configuration are given in Supplementary Appendix K (Supplementary Table S10).

### 2.9 Summary of the methodological contribution

The methodology combines ACT-R's symbolic cognitive architecture with EVisiTor's ability to perceive, acquire task-relevant knowledge, and act in a real software environment. In the Excel task, the three extensions together produce a model that is not merely an ideal task executor but a perceptual and motor agent operating through ACT-R mechanisms in the dynamic interface supplied by EVisiTor.

## 3 Results

### 3.1 Model completion-time performance analysis

The human data are available at the level of the 14 subtask windows, so these windows are the primary units for model-human comparison. The evaluation tests two distinct claims: whether the realistic model improves the mean performance fit relative to the perfect model, and whether it reproduces the variability observed across human participants.

On mean fit, the realistic model improved every error metric that is sensitive to per-subtask deviation. The task-level RMSE was 17.37 s for the realistic model vs. 29.19 s for the perfect model, a 40% reduction. The human-SD-normalized RMSE, which divides each model-human mean difference by that task's human SD, was 0.657 human-SD units for the realistic model vs. 1.240 for the perfect model. The mean absolute per-subtask error (the metric least sensitive to sign cancellation between over- and under-prediction) was 10.52 s for the realistic model and 23.11 s for the perfect model. Correlation is reported as a secondary pattern measure because it can remain high despite systematic timing error; it was *r* = 0.950 for the realistic model and *r* = 0.908 for the perfect model. Table 4 summarizes these metrics, and Figure 6 shows the subtask means. Unless noted otherwise, all model results in this and the following sections come from the eight-cell factorial batch described in the Methods (142 runs per cell).

**Figure 6:**
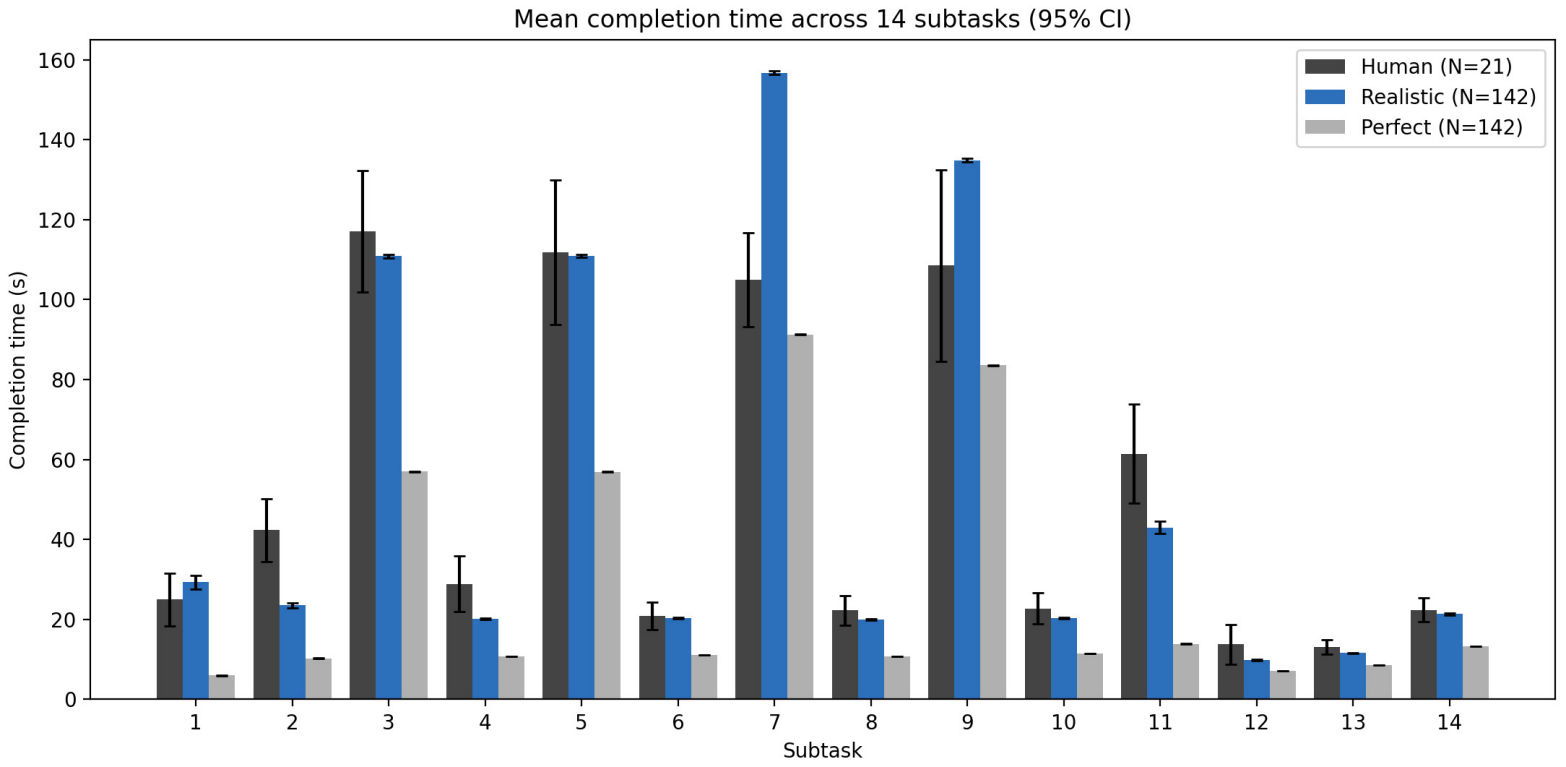
Average completion time across the 14 subtasks for humans (*N* = 21), the realistic model (*N* = 142), and the perfect model (*N* = 142). Error bars show variability across participants or runs; the human total baseline is 715.4 s.

**Table 4:** Model performance summary (142-run batch, human total 715.4 s).

Model	*r*	RMSE (s)	SD-norm RMSE	Mean abs err (s)	Model SD (s)	Total (s)
Realistic	0.950 [0.920, 0.991]	17.37 [6.46, 26.63]	0.657 [0.306, 0.986]	10.52 [4.32, 18.63]	3.05	732.6 ± 16.2
Perfect	0.908 [0.843, 0.982]	29.19 [17.41, 38.84]	1.240 [1.047, 1.413]	23.11 [14.36, 33.20]	0.27	391.9 ± 1.1
Difference	+0.043 [+0.005, +0.088]	−11.82 [−27.23, +4.02]	−0.583 [−0.981, −0.132]	−12.59 [−24.02, −0.98]	–	–

Brackets give 95% percentile bootstrap confidence intervals from 10,000 resamples of the 14 subtasks; the difference row is realistic minus perfect.

Because these metrics are computed over only 14 subtask means, we quantified their uncertainty rather than treating the point estimates as precise. Percentile bootstrap confidence intervals (10,000 resamples of the 14 subtasks) are reported alongside each metric in Table 4. The two models' individual intervals overlap on *r*, RMSE, and mean absolute error but not on human-SD-normalized RMSE, and the realistic-minus-perfect difference intervals in Table 4 exclude zero for *r*, normalized RMSE, and mean absolute error (only the RMSE difference interval includes zero). Consistent with this, the realistic model's smaller absolute error on 12 of 14 subtasks is significant by an exact paired test (Wilcoxon signed-rank *W* = 13, *p* = 0.011); Supplementary Table S14 repeats these metrics with Tasks 7 and 9 excluded. Because these subtask-mean comparisons nonetheless treat the 14 subtasks as the units of analysis, we support them below with a per-participant distributional test that uses the participants and runs as the sampling units.

The per-participant subtask windows permit a sharper distributional comparison that uses the participants and runs themselves as the sampling units. For each subtask, we computed the Wasserstein-1 distance between the 21 participants' completion-time distribution and each model's 142-run distribution, the average absolute discrepancy between two distributions, which penalizes both mean offset and unmatched spread. The realistic model's run distribution was closer to the human distribution on 12 of the 14 subtasks (the exceptions were Subtasks 7 and 9, where it overpredicts), and its mean per-subtask Wasserstein distance was 16.45 s vs. 23.44 s for the perfect model. A percentile bootstrap that resampled participants and runs (10,000 resamples) placed the 95% confidence interval for the realistic-minus-perfect difference at [−11.21, −2.52] s (bootstrap *p* = 0.002); the corresponding comparison for total completion time was 125.5 s vs. 305.6 s ([−250.1, −101.1] s, *p* < 0.001); the per-subtask distances and their sensitivity to Tasks 7 and 9 are given in Supplementary Table S15. Thus, the distributional results are statistically reliable and consistent with the mean-error results: the realistic model is closer to the human completion-time distribution than the perfect model in the primary 14-subtask analysis.

The aggregate completion time should not be read as evidence of subtask-level accuracy. The realistic model's total time (732.6 s) exceeds the human total (715.4 s) by 17.2 s (+2.4%), and that signed difference is dominated by a single subtask: Subtask 7 alone contributes +51.7 s, more than the net total difference. The largest positive deviations are Subtask 7 (+51.7 s), Subtask 9 (+26.3 s), and Subtask 1 (+4.3 s); the largest negative deviations are Subtask 11 (−18.5 s), Subtask 2 (−18.9 s), and Subtask 4 (−8.7 s). Over- and under-predictions partially cancel in the total, so fit claims are based on RMSE, human-SD-normalized RMSE, mean absolute error, and Wasserstein distance. Pearson correlation is reported only as a secondary description of rank-order pattern, not as a principal differentiating criterion.

At the subtask level, Supplementary Tables S17, S18 report the human means and standard deviations alongside the realistic and perfect model estimates.

### 3.2 Typing and error correction

Correction probability, *P*(correction), the proportion of formula entries containing at least one Backspace, pooled across all formula-entry steps and segmented identically for humans and the model (first “=” through the following Enter), was 25.0% for participants (160 of 639 entries) and 27.5% for the realistic model (1,368 of 4,970 entries across 142 runs). The realized formula keystroke error rate was 3.3%, matching the measured human formula rate supplied to the model. At the error checking rate of 0.9 for formulas and 0.85 for common words, the model produced 19.0 Backspaces per run against a human average of 18.6 per participant over the same formula segments (353 Backspaces from 19 participants). The perfect model produced no corrections (0%). The realistic model is thus behaviorally more plausible (it detects its errors by looking at the typed text and corrects them) and quantitatively calibrated on all three keystroke measures: correction probability, realized error rate, and the number of Backspaces per formula entry.

Typing speed remained the main residual discrepancy. Inter-key interval was computed identically for humans and the model: the mean of the intervals within each formula-entry segment (from the first “=” through the following Enter), over all keystrokes and with no gap cutoff. By this matched measure, the realistic model typed more slowly than humans (0.88 s vs. 0.70 s), whereas the perfect model typed considerably faster than humans (0.37 s), because its pipelined keystrokes carry no formula look-backs and no checking. The dominant contribution is the tokenized look-back mechanism: the batched-formula-typing condition, which adds look-backs but no errors or visual search, raised IKI from 0.37 s to 0.89 s, overshooting the human rate. Perceptual checking itself contributes far less: the correction-checking condition, which adds only misexecution and checking, barely moved IKI (from 0.373 s to 0.376 s). That cell types formulas without look-backs (*B* = 0), so it carries no in-progress formula check at all; the residual cost is the guaranteed whole-entry read-back before each Enter and the repair keystrokes it occasionally triggers. Visual search left IKI unchanged at the perfect-model baseline (0.37 s), as it does not touch the typing pathway.

These timings also reflect how keystrokes are scheduled. A fully serial scheme required the manual (motor) module to be entirely free before the next keystroke (the ?manual> state free query), so a key could not begin until the previous key-press had passed through every motor stage (i.e., feature preparation, initiation, execution, and the finishing return to rest) with no overlap between successive keys; under that fully serial scheme the idealized model reproduced the human mean IKI (about 0.70 s) but matched humans on essentially nothing else, as its task completion times and deviation-sensitive fit were poor. Because users do not fully complete one finger movement before starting the next, the model used here instead schedules each keystroke as soon as the motor processor is free (the ?manual> processor free query): ACT-R's manual module advances through separate preparation, processor, and execution stages, and the processor frees once a movement has been prepared and initiated, before its execution and finishing return complete, so the model can prepare and launch the next keystroke while the current one is still being executed. The realistic model's behavior under this pipelined scheme, with the residual slowdown localizing to the look-back and checking mechanisms rather than to raw motor cost, indicates it is the more faithful account of spreadsheet typing. Future versions should add practice effects and larger formula chunks for repeated simple formulas, user pattern recognition in formulas and could overlap checking with the next keystroke as skilled typists do.

### 3.3 Ablation study

The ablation crossed the three mechanism groups in a complete 2 × 2 × 2 factorial design: every combination of visual search (*V*), batched formula typing (*B*), and the error-and-correction package (*C*) was run for 142 seed-matched runs, giving the eight cells of Supplementary Table S19. The mechanisms are operationally separable in the implementation: each factor can be enabled or disabled independently, and every run's configuration manifest records the actual state of every switch. Unlike a mechanism-addition design that enables one mechanism at a time, the full factorial allows interactions between mechanisms to be estimated rather than assumed (see Table 5). Supplementary Tables S19, S20 report what each cell does to human-fit and process measures. The process measures confirm the intended switch specificity: visual search changes attention and search behavior without changing formula inter-key intervals, formula look-backs and correction mechanisms change typing-related measures (i.e., look-backs through pauses between token batches, and perceptual checking through check-driven attention shifts to the typed text) without creating visual-search events, and factor-off mechanisms remain genuinely off in the combination cells.

**Table 5:** Saturated 2 × 2 × 2 factorial estimates for total completion time (effect coding −0.5/+0.5; 142 runs per cell; stratified bootstrap with 10,000 resamples).

Effect	Estimate (s)	95% CI	*p*
*V* (visual search)	+86.78	[+85.09, +88.50]	< 0.001
*B* (batched formula typing)	+197.74	[+196.02, +199.46]	< 0.001
*C* (errors and correction)	+56.18	[+54.47, +57.89]	< 0.001
*V* × *B*	+0.99	[−2.42, +4.30]	0.58
*V* × *C*	−0.53	[−3.88, +2.89]	0.76
*B* × *C*	−23.04	[−26.49, −19.69]	< 0.001
*V* × *B* × *C*	+0.17	[−6.64, +6.89]	0.98

Estimates are in seconds with 95% percentile confidence intervals and two-sided bootstrap p values (resolution 2/10,000).

The conditions separate fit improvement from process realism. Visual search was the main contributor to attention shifts: enabling it alone lowered RMSE from 29.19 s to 23.68 s and raised *r* from 0.908 to 0.924. The single largest mean-fit gain, however, came from batched formula typing, which alone lowered RMSE to 20.72 s; correction checking alone lowered it to 24.19 s. Attention shifts rise from 524 per run in the perfect model to about 1,701 in the full model, the increase coming from visual search for targets and from the 164 per-run formula look-backs. Perceptual checking does not appear as a separate class of shift in this model: rather than issuing a distinct attention shift per check, the model keeps the already-attended entry field encoded and re-encodes it automatically as characters arrive, with an explicit shift to the typing area once per entry (38 per run). Motor errors and perceptual correction checking added keystrokes (481 to 528 per run, most of the increase being end-of-entry repair retyping), produced the error-repair behavior of the previous section, and because checking and repair cost real time contributed a fit improvement of their own. Each mechanism adds interaction cost, moving completion time upward from the perfect baseline (391.9 s) through the human range (Figure 7). The per-cell process measures behind these counts are given in Supplementary Table S20, and the per-subtask breakdown of each mechanism's increment in Supplementary Table S16.

**Figure 7:**
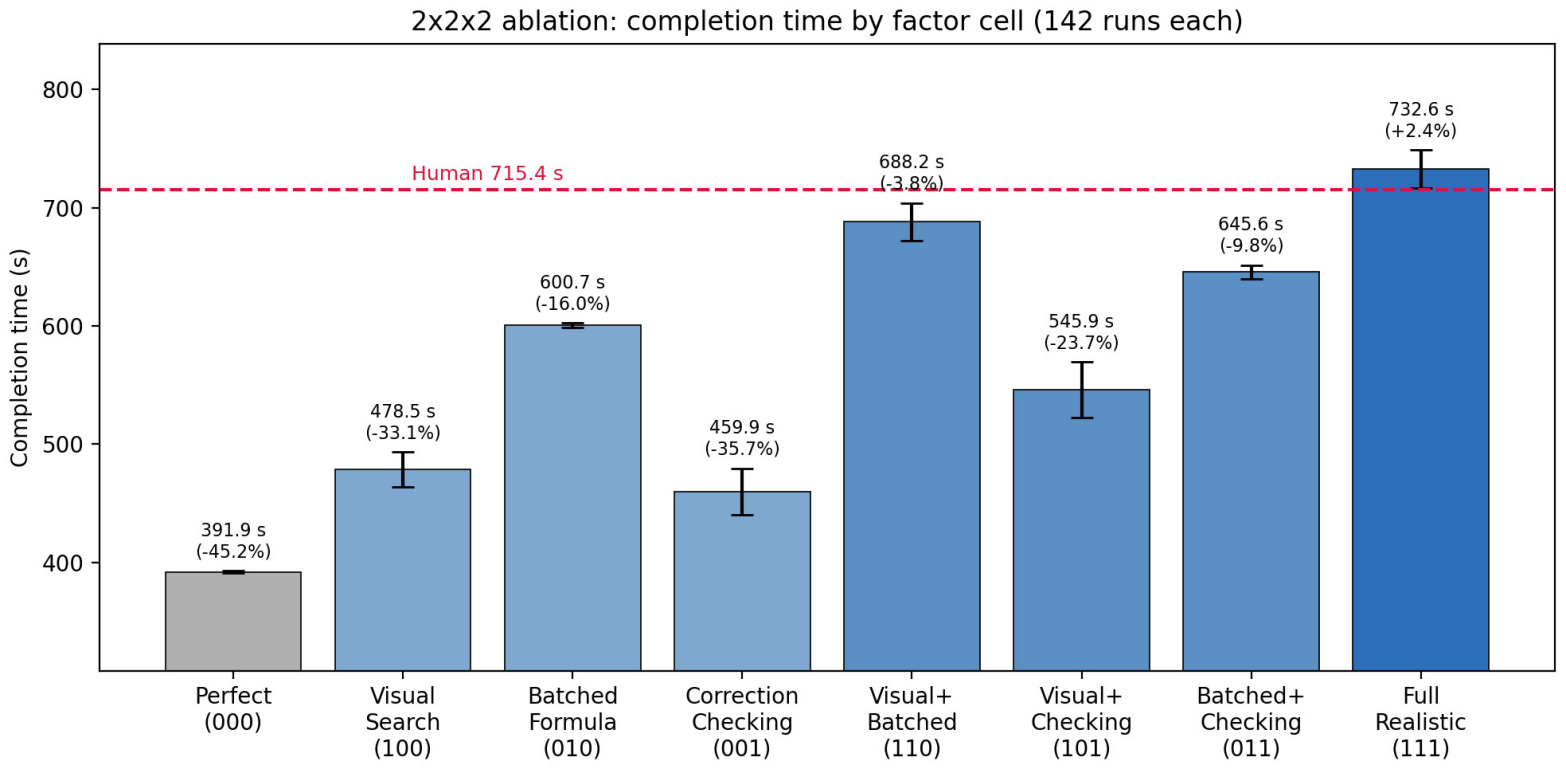
Completion time by ablation cell (complete 2 × 2 × 2 factorial, 142 runs each). Bars show mean total time, error bars show ±1 SD, and the dashed line marks the human mean total (715.4 s).

### 3.4 Mechanism interactions: factorial estimates

Because the eight cells form a complete factorial, each mechanism's contribution can be estimated while averaging over the states of the other two, and interactions can be tested rather than assumed. For total completion time and for each subtask, we fit the saturated model time ~*V* × *B* × *C* with effect coding (−0.5, +0.5), so each main effect is the average total-time change produced by enabling that mechanism and each interaction coefficient is the average change in one mechanism's effect when another is enabled. Uncertainty was quantified with a stratified bootstrap that resampled runs independently within each of the eight cells (10,000 resamples); Table 5 reports the estimates with 95% confidence intervals and two-sided bootstrap p values.

All three main effects are large and significant: enabling visual search adds 86.8 s [85.1, 88.5] to total completion time, batched formula typing adds 197.7 s [196.0, 199.5], and the error-and-correction package adds 56.2 s [54.5, 57.9] (all *p* < 0.001). The only significant interaction is *B* × *C*, and it is sub-additive: −23.0 s [−26.5, −19.7] (*p* < 0.001; −23.1 s at *V* = 0 and −23.0 s at *V* = 1). The error-and-correction package therefore costs about 23 s less when formula typing is token-batched: it adds 68.0 s to unbatched typing (391.9 s to 459.9 s) but only 44.9 s to batched typing (600.7 s to 645.6 s). This is a smaller increment, not a cheaper condition: batched formula typing is the most expensive mechanism in the model, and both *B* = 1 (Batched typing) cells remain far slower than their *B* = 0 counterparts. Under batching, every completed nonfinal token already returns attention to the formula source, so a formula check rides along at each token boundary and a slip is detected at most one token after it is made; without batching there are no such boundaries: no per-keystroke or per-token check probability applies during formula typing, so the model types the whole formula straight through and detection falls entirely to the pre-Enter read-back. Because repair deletes back to the first wrong character and retypes the tail, later detection means longer repairs, and the process measures carry that signature: the typo rate is essentially unchanged between the two checking cells (16.3 vs. 14.7 per run) while correction deletes fall from 70.8 to 22.5 per run. With Holm correction across the 14 subtasks, the interaction is significant and negative on eight subtasks, concentrated on the long formula entries (Subtask 3: −6.7 s [−7.9, −5.5]; Subtask 5: −6.9 s [−8.2, −5.7]; Subtask 7: −3.8 s [−4.8, −2.8]), with smaller effects on Subtasks 4, 6, 8, 9, and 10 (−0.8 to −1.2 s); all Holm-adjusted *p* ≤ 0.007. No interaction involving visual search is significant (*V* × *B*: +1.0 s [−2.4, +4.3], *p* = 0.58; *V* × *C*: −0.5 s [−3.9, +2.9], *p* = 0.76; *V* × *B* × *C*: +0.2 s [−6.6, +6.9], *p* = 0.98): the perceptual cost of finding targets combines additively with the typing-side mechanisms. We retain the full realistic cell (111) as the paper's behavioral model.

### 3.5 Error-rate calibration and sensitivity analysis

The realistic model's two typing-error rates were measured from the human keystroke logs rather than fitted (0.034 per formula keystroke and 0.063 per word keystroke) and were supplied directly to the model. A sensitivity analysis that varied each rate independently from 0% to 5% (holding the other at its measured value, with visual search disabled to isolate the manipulation) confirms that the measured formula rate is also the behaviorally correct operating point: pooled *P*(correction) rises from 1.0% to 38.7% across the formula sweep and crosses the human 25.0% value at the 3% condition (25.3%), that is, at the independently measured human rate; the word rate has little leverage because the model types roughly nine times as many formula keystrokes as word keystrokes; and RMSE moves by about 1 s across the entire range. The full sweeps with per-condition completion times, correction probabilities, realized error rates, and RMSE values with 95% bootstrap confidence intervals (Supplementary Table S11), together with the sensitivity figure (Supplementary Figure S4), are reported in Supplementary Appendix L.1. A companion sweep of the formula-check probability itself (Supplementary Table S12, Supplementary Appendix L.2) shows that fit is likewise flat across that grid, so the a priori value of 0.90 was retained rather than tuned.

### 3.6 Knowledge-acquisition accuracy: instruction-to-chunk conversion

To evaluate the Knowledge Acquisition extension of VisiTor, we tested whether large language models could convert the natural-language task instructions given to human participants into the executable action-chunk representation consumed by the ACT-R model, for example (Goto “B7”) (Select “B7”) (end) followed by (Type “=(c7*b13)/100”) (Press “enter”) (end). Generated structures were scored against a modeler-defined gold-standard decomposition of the full 14-subtask spreadsheet protocol, consisting of 94 subtask chunks (Supplementary Appendix G, Supplementary Table S6; scoring criteria in Supplementary Appendix I, with the per-model scores repeated in Supplementary Table S8). To evaluate the robustness to wording, each model was run on the original instruction text and on two independent paraphrases that preserved subtask content but varied phrasing (Supplementary Table S5).

Six models from three providers were evaluated: OpenAI GPT-5.5 (OpenAI, 2026), GPT-5.4-mini, and GPT-5.4-nano; Google Gemini-3.5-Flash and Gemini-3.1-Flash-Lite; and the open-weight openai/GPT-OSS-120b served through OpenRouter. All six models were accessed through the same OpenAI-compatible client with identical decoding settings (temperature 0.2, top-p 0.95, maximum 512 output tokens, and no streaming); outputs were generated in a single pass per instruction variant, with no retries and no manual repair before scoring. The GPT-OSS-120b evaluation was an early pilot run performed with the model's public release build (5 August 2025; OpenAI, 2025); we retained it because gpt-oss-120b remains one of the most widely used open-weight baselines. The exact model identifiers, providers, access dates, API endpoints, and parameter settings are reported in Supplementary Appendix D (Supplementary Table S4), and the archived request/response payloads in Supplementary Appendix H (Supplementary Table S7). Because these are evolving hosted endpoints, exact reproduction of the external LLM calls is not guaranteed; the reported results are snapshots of the accessed versions, and the prompts, gold structures, and generated structures needed to re-score any replacement model are archived in the repository.

We report three measures: schema validity (the fraction of generated outputs that parse into the chunk schema without repair), action-verb accuracy (Goto, Move, Select, Type, or Press chosen correctly), and target accuracy (the cell, menu item, formula, or key extracted correctly). Table 6 reports schema validity, mean action-verb accuracy, and target accuracy for each of the three instruction variants.

**Table 6:** Instruction-to-chunk conversion accuracy by model.

Model	Provider	Schema valid	Action acc.	Target orig.	Target P1	Target P2
GPT-5.5	OpenAI	100%	99.5%	90.3%	96.8%	89.8%
Gemini-3.5-Flash	Google	100%	99.5%	94.6%	92.5%	96.8%
GPT-5.4-mini	OpenAI	100%	76.6%	90.3%	88.2%	10.7%
GPT-5.4-nano	OpenAI	100%	68.9%	89.9%	86.0%	10.6%
Gemini-3.1-Flash-Lite	Google	100%	77.4%	91.4%	92.5%	12.8%
GPT-OSS-120b	OpenRouter	100%	78.2%	90.3%	88.2%	17.7%

Schema validity and action-verb accuracy are averaged across the three instruction variants; target accuracy is shown separately for the original instructions and the two paraphrases (Para-1 and Para-2).

All six spreadsheet models produced schema-valid output. On the original instructions and the first paraphrase, target accuracy was 86%–97%; the smaller models were less robust to the second paraphrase. GPT-5.5's target accuracy on Paraphrase 2 was 89.8%, seven percentage points below Gemini-3.5-Flash and slightly below its own original-instruction score; this modest decline differs qualitatively from the smaller models' drop to 10.6%–17.7%. Averaged across the three instruction variants, target accuracy was 94.6% for Gemini-3.5-Flash and 92.3% for GPT-5.5. The remaining mismatches were dominated by interface-naming conventions rather than failures to understand the requested operation in the instructions. A state-correct UIA-grounding follow-up made this distinction explicit: when the prompt contained only the live objects available in the relevant interface state and required verbatim use of their names, the model selected the intended object for every scannable visual target. The sole unresolved target was the Save dialog's file-name input, whose UIA name changes with its contents and therefore provides no stable “file_name” label. Thus UIA grounding brought the spreadsheet visual-object mapping close to ceiling except for an interface element that UIA itself does not name stably. We suspect Save dialog's file-name can also be found if a screenshot of the environment was given to the model alongside the UIA-grounding in the prompt but did not test it.

The conversion also quantifies the manual-authoring savings claimed for the Knowledge Acquisition pathway. From the 14 natural-language task instructions, the pipeline generated 94 subtask chunks and 187 atomic action items, for a total of 281 declarative structures that would otherwise have to be modeler-defined. It is important to note that the modeler is still responsible for providing the chunk schema and the action vocabulary, which can be reused across tasks. This supports the claim that the Knowledge Acquisition extension can reduce manual chunk-authoring burden while remaining interpretable and schema-constrained.

To test whether generated structures were not only syntactically valid but also operational in the ACT-R model, we ran the spreadsheet model using Gemini-generated task structures after adding the visual objects UIA naming constraints. The generated structures successfully executed the model. The state-correct UIA analysis further showed that every scannable visual target was grounded to the intended live object, with only the unstable file-name input remaining outside the UIA naming scheme. Because the normalized action structures used for the execution check were equivalent to the modeler-defined gold-standard structures, downstream performance was identical by construction; this is an executability result rather than an independent model-fit condition (Supplementary Appendix J, Supplementary Table S9).

### 3.7 Generalization analysis: novel Windows tasks from a public computer-use dataset

To test whether EVisiTor's perception and knowledge-acquisition pathways were limited to Excel, we selected ten novel tasks from the public Paradigm Shift AI computer-use dataset (Howland et al., 2025). The tasks span the Run dialog, File Explorer navigation and command bars, Windows context menus, taskbar search, and File Explorer search (Supplementary Table S21). Four tasks were excluded for cause, which we report because the exclusions illustrate the method's current boundary (Supplementary Appendix M, Supplementary Table S13): three had under-specified demonstrations (an unstated confirmation dialog, an instruction with no resolvable target, and an unstated data range), and one, reading command output in a terminal, was excluded because UI Automation does not expose terminal text as elements, so the perception pathway itself does not apply there.

For each task, we created a modeler-defined gold decomposition of the method shown in the recorded demonstration, reconciled its visual target labels with the UIA output available to EVisiTor, and validated the gold chunks in live execution. This instruction to chunk mapping reference contains 21 subtask chunks and 39 atomic actions. One mapping deserves emphasis because it changed the gold itself. The demonstrated method for W03 creates the folder by right-clicking an unoccupied area of the folder view and choosing New > Folder; that unoccupied area is not a visual item in the UI Automation tree. No scan exposes an element named ‘Desktop background.' A gold step anchored on that region can therefore never be grounded in what the model (or a UIA-grounded LLM) actually perceives, which is why this step failed to reproduce. Because the same effect is available through real, scannable interface objects, the W03 gold and guide were revised to create the folder with the Ctrl+Shift+N chord (clicking the command-bar New > Folder entry is an equivalent groundable route, and the models perform either perfectly). This gold is one executable reference path, not the only correct solution. Broad instructions such as “open a terminal” or “create a folder” do not specify a unique method: a user may use a keyboard shortcut, taskbar search, a context menu, or another valid route. A generated sequence can therefore differ from the gold and still be correct.

Seven LLMs generated chunks from two forms of each task description. The broad-instruction condition used the dataset sentence; the detailed-guide condition specified the intended method step by step, parallel to the detailed spreadsheet instructions. Each form was tested without UIA and with full UIA grounding, in which the prompt contained the union of live UIA element names captured for the task. Exact agreement was computed over the flat action sequence and deliberately ignored subtask numbering, because models break the same procedure into subtasks differently. In W04, for example, all seven models given the guide without UIA generated exactly the gold's nine actions, but grouped them differently: the gold treats the Send-to cascade (locating and selecting “Send to,” then locating and selecting “Compressed (zipped) folder” as one four-action subtask, whereas the models split it into two two-action subtasks. Such parses count as exact matches, because the grouping does not affect the model's performance: execution proceeds action by action, so the same actions in the same order produce the same behavior regardless of how they are numbered into subtasks. For broad instructions only, a deterministic functional check also asked whether the generated actions would accomplish the task from its actual start state, including known-valid alternative Windows methods.

For the broad dataset instructions, exact action agreement with the gold ranged from 42.5% to 63.6% without UIA and from 18.8% to 70.6% with full UIA (Table 7). Those values should not be interpreted as task-success rates: 41 of 42 no-UIA parses and 41 of 42 full-UIA parses were sufficient to complete the task. Most disagreements were valid method choices, such as Win+E instead of clicking File Explorer, F2 or the context menu instead of the Rename toolbar button, the context-menu New > Folder path instead of the Ctrl+Shift+N chord. UIA grounding made generated targets resolvable against the live interface, but it did not force the model to choose the demonstrator's method and therefore did not necessarily increase exact gold agreement (DeepSeek-V4-Flash, for example, dropped to 18.8% exact under full UIA while remaining functionally sufficient on all six tasks).

**Table 7:** Gold-sequence agreement and task sufficiency for the six-task Windows analysis (21 gold subtasks, 39 atomic actions).

Model	Instr. exact No UIA (%)	Instr. exact Full UIA (%)	Instr. tasks done No/full UIA	Guide exact No UIA (%)	Guide exact Full UIA (%)
GPT-5.5	63.6	60.0	6/6; 6/6	82.5	90.0
GPT-5.4-mini	51.6	70.6	6/6; 6/6	82.5	90.0
GPT-5.4-nano	45.0	40.9	6/6; 6/6	70.0	76.2
Gemini-3.1-Flash-Lite	50.0	64.7	6/6; 6/6	82.5	90.0
GPT-OSS-120b	46.9	58.3	5/6; 5/6	85.0	90.0
DeepSeek-V4-Flash	51.4	18.8	6/6; 6/6	82.5	81.0
Nemotron-3-Super	42.5	50.0	6/6; 6/6	82.5	90.0

“Exact” is flat action-level verb-and-target agreement with the modeler-defined gold. “Task completion” is reported only for the broad-instruction condition and counts gold matches plus validated alternative paths; each cell gives no-UIA; full-UIA.

The detailed guide tested a different question: when the desired procedure is stated explicitly, can the LLM reproduce that procedure? Here we compare only against the gold sequence because the guide defines how the task is intended to be performed. Exact agreement rose to 70.0%–85.0% without UIA and 76.2%–90.0% with full UIA across the seven models; GPT-5.4-nano, the smallest model, sets both lower bounds, and the remaining six models reached 82.5%–85.0% and 81.0%–90.0%. More informative than the aggregate is what the residual mismatches actually are. Before the W03 revision described above, the single largest source of disagreement was the ungroundable right-click step: every model paraphrased or re-grounded the nonexistent “Desktop background” target (ungrounded models echoed the guide's wording (“empty background,” “Desktop folder view”), grounded models substituted the nearest real element (“Items View,” “This folder is empty.”), and models that merged the look-then-right-click pair into a single action shifted the alignment of every subsequent step). After the revision, every model reproduced the W03 sequence exactly in both grounding conditions. The remaining disagreements fall into two small categories, neither of which is a strategy error. First, naming granularity without grounding: models echo the guide's descriptive phrasing where the gold uses the interface's accessible name (“Search box” for the taskbar element named “Search,” “Calculator window” for “Calculator”); these disappear under full UIA, where the model uses the scanned names verbatim. Second, grounded models occasionally insert a legitimate motor step that the gold leaves implicit, such as clicking the Run dialog's “Open:” field before typing the command.

The full verbatim guides used for W01, W02, W03, W04, W09, and W10 are provided in Supplementary Appendix M.1. These results show that two things jointly determine whether generated chunks match a human-authored decomposition: the instruction must specify the intended method, and the gold itself must name real, perceivable interface objects.

For the executability claim, the task-independent generic model driven by the validated gold chunks completed all six tasks in live Windows 11 through real UIA perception and real mouse and keyboard output. Completion was verified from system state (i.e., applications launched, files renamed or created, an archive produced, and the requested file found) rather than from the model merely halting. For W03, the model created the folder with the Ctrl+Shift+N chord, completing live in 12.4 s with the created folder verified on the Desktop. Supplementary Table S21 reports the single human demonstration time and the model's live simulated time for descriptive context; no parameter was fit to these six observations.

The main contribution of this work is to show that two traditionally modeler-authored inputs to an ACT-R model can be automated: EVisiTor builds the model's perceptual representation from the live interface through UI Automation, and it can acquire task knowledge by converting human-readable instructions into executable declarative chunks. The Excel behavioral study is a use case that exercises and showcases these capabilities in a complete perception-cognition-action loop; it is not the sole contribution and does not imply a complete cognitive model of Excel expertise. The six Windows tasks tested the portability of these capabilities beyond the spreadsheet setting. In the live demonstrations, the unchanged task-independent model executed validated gold chunks through live perception and motor output; in the complementary parsing evaluation, LLMs produced functionally sufficient chunks for nearly all broad instructions. Because the generated chunks were not the chunks used in the six live demonstrations, these are complementary tests of knowledge acquisition and execution portability, not a single end-to-end behavioral validation.

The analysis therefore supports a bounded generalization claim: EVisiTor's environment perception, instruction-to-chunk conversion, and end-to-end execution are not limited to Excel. It also shows that knowledge extraction is strongly dependent on the precision of the instruction. A mismatch with one gold path is not evidence that a generated path is wrong when the task admits several valid strategies; if a particular behavior is required, the instruction must specify that behavior. Because each Windows task has only one recorded demonstration rather than a participant sample, the analysis is not a second model-fit study and no distributional timing claim is made.

## 4 Discussion

The spreadsheet behavioral results provide a use-case evaluation of the EVisiTor framework rather than defining the paper's primary contribution. That contribution is the automation of live interface perception and instruction-based knowledge acquisition for a symbolic cognitive model. The Excel case shows what these capabilities enable when embedded in a complete perception-cognition-action loop and compared with human behavior. Across the 14 subtasks, the realistic model reduced task-level RMSE from 29.19 s to 17.37 s (40%), human-SD-normalized RMSE from 1.240 to 0.657, and mean absolute per-subtask error from 23.1 s to 10.5 s. The realistic total (733 s) overshoots the human total (715 s) by 2.4%, largely because of Task 7, and formal uncertainty quantification shows the realistic model closer on 12 of 14 subtasks, with the full-sample distributional (Wasserstein) difference reaching statistical significance.

The perfect model is useful as an ideal-performance baseline, but it is not behaviorally plausible because it assumes error-free execution and direct access to task-relevant object locations. Real spreadsheet users must read instructions, locate cells and control objects, enter formulas, check their work, recover from mistakes, and sometimes hesitate or reorient (not addressed in this paper). The realistic model improves the fit by enhancing the modeling of perceptual and motor cognitive processes through EVisiTor, although it should not be interpreted as a complete account of all human strategies or individual differences.

The largest improvement came from mechanisms that added realistic interaction costs where the perfect model was unrealistically efficient. Batched formula typing gave the largest deviation-sensitive fit gain of any single mechanism, reducing RMSE from 29.19 s to 20.72 s when enabled alone, because the formula-entry subtasks dominate total task time. Visual search reduced error on the interface-navigation subtasks, where the perfect model was implausibly fast because it was handed its targets: enabling it alone reduced RMSE to 23.68 s. Its cost is sharply localized to the subtasks that require finding menus, dialog fields, and row headers rather than typing into cells, adding 23.3 s, 11.2 s, and 28.2 s on Tasks 1, 2, and 11 and under 1 s on each of the typing-dominated formula subtasks (Supplementary Table S16). Task 14 is instructive precisely because it is cheap: it repeats the Save-a-Copy navigation of Task 2 and costs only 2.2 s, because the model reuses the locations it has already stored in remembered-location memory rather than searching for them again. The mechanism therefore behaves as a spatial-memory-backed search rather than a flat per-action tax. Motor misexecution and perceptual correction checking added keystrokes and error-repair behavior and improved RMSE to 24.19 s when enabled alone. These mechanisms are calibrated from human keystroke data: the realistic model produced corrections close to the human rate (27.5% vs. 25.0% under the same pooled definition), matched the human formula-Backspace count (19.0 model Backspaces per run vs. 18.6 per human participant), and its realized formula keystroke-error rate (3.3%) matched the measured human rate.

The factorial ablation adds an interaction-level result to this mechanism story: the three mechanism groups combine near-additively, the one exception being a significant sub-additive B × C interaction of about 23 s. Checking and repair cost less when formula typing is token-batched (44.9 s rather than 68.0 s) because token boundaries already return attention to the formula source, so slips are caught within a token and the deleted-and-retyped tail stays short. The saving is concentrated on the long formula subtasks (Tasks 3, 4, 5, 6, 7, 8, 9, and 10), and it is a saving on the increment only: batched typing remains the single most expensive mechanism in the model (198 s). No interaction involving visual search was significant, so the perceptual-search cost the model adds is statistically separable from the typing-side mechanisms; this supports treating the visual-search improvement and the typing-side deviations as independent modeling problems, and identifies batched formula typing as the locus of the Task 7 overshoot.

The variability results reveal both a success and a limitation. Visual search substantially improves the approximation of participant-to-participant variability across the four interface-navigation subtasks it governs (Tasks 1, 2, 11, and 14): mean between-run SD rises from 0.51 s without visual search to 6.34 s with it, and on Task 1 the full model's SD (10.83 s) approaches the human SD (15.44 s) without reaching it. We interpret this as a partial approximation, not a reproduction of the participant distribution. The typing mechanism tells the same story. On formula-entry Tasks 3, 5, 7, and 9, the model varies far less than participants (between-run SD about 3 s, supplied mainly by the stochastic checking and repair mechanism) while human SDs range from 27 to 56 s. Because the spreadsheet procedure is dominated by formula entry, the human mean subtask SD across all 14 tasks (20.6 s) remains much larger than the realistic model's mean subtask SD (3.0 s), and the perfect model is nearly deterministic throughout. The remaining task-specific discrepancies suggest what might be missing: participants differ in cognitive load, Excel expertise, formula familiarity, how much of a formula they encode as one chunk, how often they look back to the instruction, and when they check or recheck their entry. The model's overprediction on Task 7, where participants typed repeated formulas relatively quickly, is consistent with formula chunks that are too small or too costly, a gap that the factorial analysis localizes to batched typing and its interaction with checking and repair. Future work should model chunk-size selection, practice, instruction encoding, and checking strategy as sources of individual differences. The defensible conclusion is that grounding the model in actual interface perception reduces aggregate deviation-sensitive error across the 14 tasks and adds meaningful variability to visual-search behavior, while the current typing implementation, even though it improves the model's overall performance, does not yet reproduce participant-to-participant variability.

The Tasks 7/9 require a long sequence of closely related formulas. Human users can find a pattern within the sequence, reuse a formula template or a larger memory chunk, type from memory after the pattern is learned, and adapt when and how often they check. The current model uses fixed formula tokenization, repeated instruction look-backs, and a largely fixed perceptual-checking policy, so it does not represent those spreadsheet-specific strategy changes. The primary conclusion therefore remains based on all 14 subtasks; the restricted analysis reported in Supplementary Appendix O (Supplementary Tables S14, S15) identifies both where the EVisiTor mechanisms improve fit and where the spreadsheet typing model requires further development.

The generalization analysis clarifies what should and should not be claimed about task generality. A single task-independent cognitive model completed six novel Windows tasks through live perception and motor output, demonstrating that the EVisiTor pathways are not spreadsheet-specific. At the same time, broad instructions produced many valid strategies that differed from the recorded demonstrator's path. Exact agreement therefore measured procedural agreement, not correctness: nearly every instruction was functionally sufficient even when its chunks differed from the gold. However, different models chose different methods to complete the task. When the intended method was supplied in the prompt as a detailed guide, gold agreement rose substantially. The result is an instruction-design finding as much as a model finding: interface vocabulary can be grounded automatically with UIA, but a particular procedure must be stated if that procedure is what the model is expected to acquire. Model-fit claims remain limited to the spreadsheet study because the Windows tasks have single demonstrations rather than participant samples.

Overall, these results support integrating EVisiTor-enabled environment perception and instruction acquisition with ACT-R cognitive simulation: the resulting framework is more flexible and behaviorally plausible than an idealized symbolic executor.

### 4.1 Limitations

The scope limitation should be interpreted relative to the paper's primary objective: to demonstrate that EVisiTor can automate live interface perception and instruction-based knowledge acquisition for a cognitive model. We selected the spreadsheet procedure as a demanding use case because it exercises the complete interaction loop, from perceiving interface objects and finding targets to typing, slipping, checking, and correcting. The 40% RMSE and 54% mean-absolute-error reductions relative to the perfect-user baseline are therefore evidence that the automated pathways produce behaviorally consequential mechanisms, not a claim that Excel modeling is the central contribution or that the current model captures every Excel strategy.

The six Windows tasks serve a different purpose. They show that EVisiTor's environment perception and knowledge-extraction pathways are not limited to Excel. They also expose a substantive dependence on instruction detail. A request such as “open a terminal” can be satisfied through Win+X and a menu key, taskbar search, or a File Explorer context menu; people likewise may choose different valid methods. Consequently, deviation from a human-authored gold sequence does not by itself make an LLM-generated chunk sequence incorrect. If the model must reproduce a particular method, the instruction must explicitly specify that method, as in the guide condition.

Other limitations remain. The eye-tracking analysis is exploratory because only eight usable gaze-overlay videos were available and participant-level gaze accuracy and data loss were not fully reported in the original dataset, so gaze findings are treated as motivation for model assumptions rather than inferential evidence. The model underrepresents between-participant variability, especially in formula entry. This discrepancy may be the result of expertise, formula familiarity, chunking, cognitive load, and checking strategy. In particular, the present results show that the model still fails to capture nuances of human formula typing: participants typed repeated formulas (most visibly in Task 7) considerably faster than the model's token-by-token pace, human formula entry is far more variable than the model's timing, and the model's fixed checking rate and mandatory pre-Enter read-back likely overstate how uniformly skilled typists monitor their typed text. Future work needs to address these nuances (i.e., practice and chunk-size growth over repeated formulas, pattern recognition in formulas, expertise-dependent look-back and checking strategies, and adaptive monitoring granularity) and, more broadly, to investigate the typing cognitive process in spreadsheets and update the typing procedural process. The human sample is narrow and drawn from one university which may skew the completion time distribution. Typing and correction parameters were measured from the same participant pool used for evaluation; although they were derived from keystroke counts rather than fit to completion times, participant-level cross-validation on an independent sample remains needed. Finally, the instruction-to-chunk accuracies depend on hosted and evolving LLM endpoints and should be treated as snapshots of the accessed versions.

Results show that the EVisiTor-enabled model improves mean human-performance fit and process realism relative to a perfect-user baseline. They do not show that the model fully captures the distribution of human behavior, all sources of typing error, or all strategies used by novice and expert spreadsheet users.

### 4.2 Future work

Future work should improve generality, speed, and calibration. UI Automation was useful for Excel, but it is only one perception pathway and depends on the accessibility structure exposed by the target application. Future versions of EVisiTor should support interchangeable perception backends, including accessibility-tree extraction, application-specific parsers, screenshot-based GUI parsers, and specialized screen-parsing models. The relevant evaluation criterion for cognitive modeling is not only aggregate object-detection accuracy, but whether task-critical objects such as cells, row and column headers, menus, dialog fields, and dynamically appearing overlays are preserved with sufficient spatial precision for ACT-R visual-location requests.

We also tested whether general-purpose vision-language models and screenshot-based GUI parsers could serve directly as EVisiTor's perception backend, replacing UI Automation. They could not: across the models we tried, task-relevant objects were missed, adjacent spreadsheet cells were merged, labels were hallucinated, and bounding boxes were too imprecise for ACT-R visual-location requests. These attempts are reported in Supplementary Appendix N (Supplementary Figures S5–S7) and should be read as future-direction evidence rather than as validation results. We note them because the negative result motivates the hybrid perception architecture described above, in which UI Automation and application-specific extractors handle spatial grounding while vision-language models are reserved for semantic interpretation of unfamiliar layouts.

Web-based interfaces add another challenge because the DOM, browser accessibility tree, and rendered visual scene may not contain the same object decomposition. In preliminary Google Sheets tests, UIA exposed menus and toolbar controls more reliably than the spreadsheet grid. A custom JavaScript userscript provided more controlled extraction of visible spreadsheet objects and overlays, but this approach is application-specific and must be constrained to visible or visually inferable elements. Figure 8 shows examples of this application-specific extraction pathway.

**Figure 8:**
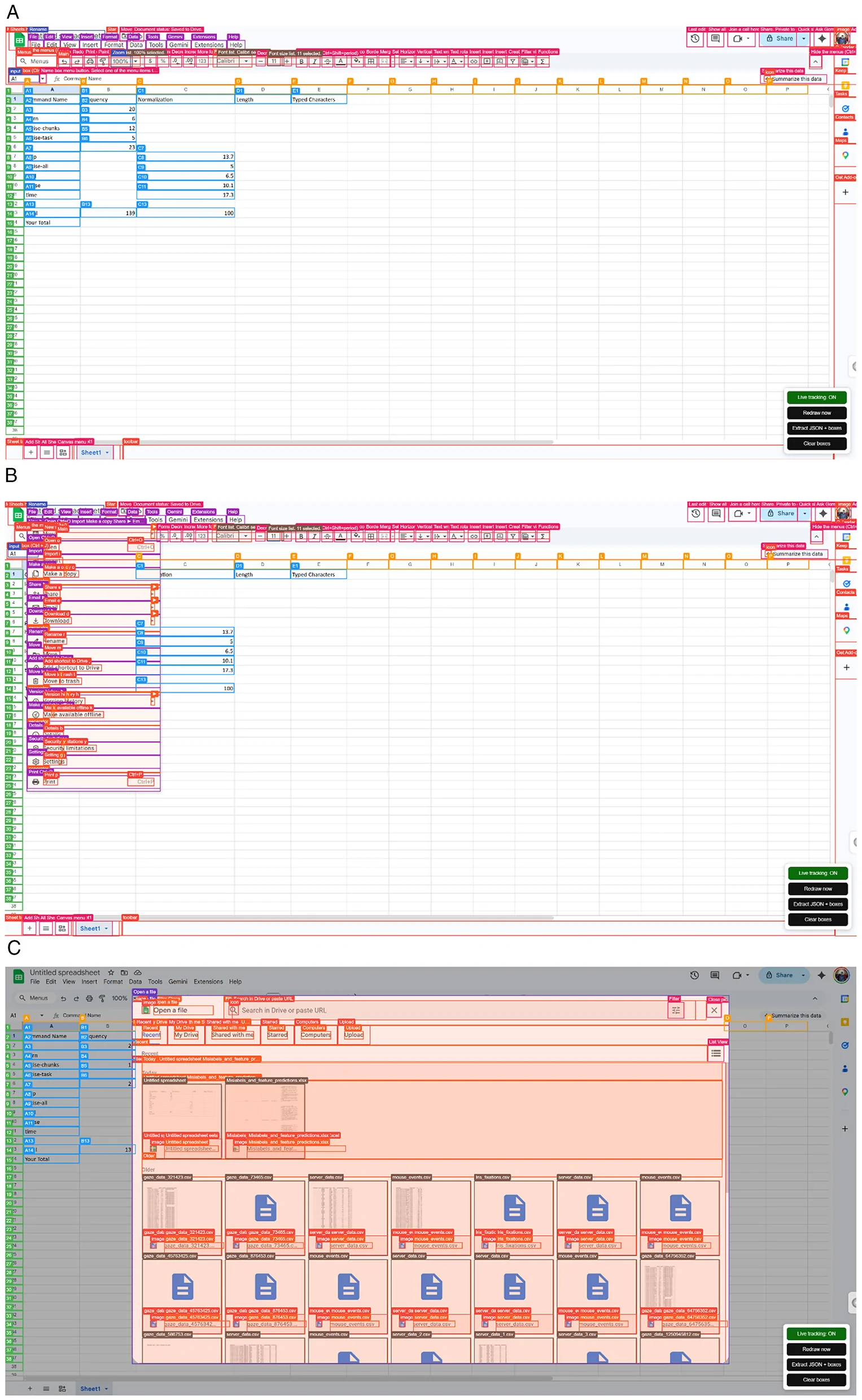
Visual-environment parsing in Google Sheets using a custom JavaScript userscript executed through Tampermonkey. The extractor identifies visible UI objects and overlays their bounding boxes on the rendered interface across different application states: **(A)** the spreadsheet grid and toolbar in the default view, **(B)** an opened drop-down menu, and **(C)** a pop-up/file-selection dialog displayed above the spreadsheet. These examples illustrate that the application-specific extractor can parse both the underlying spreadsheet interface and dynamically displayed overlay elements within the supported Google Sheets environment.

## 5 Conclusion

This work addressed a central limitation in cognitive models of interface use: the gap between models that simulate internal cognition and models that can perceive, act, acquire task knowledge, and adapt within the same interface environments used by human participants. By extending VisiTor, this study moved toward an AI-augmented cognitive-simulation framework in which an ACT-R model can convert task instructions into declarative knowledge structures, perceive visual objects in an external software environment, execute erroneous actions through the operating system, and detect and correct those actions.

The knowledge-acquisition component was evaluated both as an Instruction-to-chunk conversion task and as an end-to-end execution check. In the spreadsheet analysis, adding UIA objects to the prompt made the model select the intended live visual object for every scannable target; the only exception was the file-name input, which has no stable UIA name. The normalized generated structures used in the execution check were equivalent to the modeler-defined gold, so downstream behavior was identical by construction, an executability result rather than an independent behavioral finding. In the Windows analysis, gold chunks were validated against live UIA output and executed across six tasks. Broad instructions often produced different but functional methods, whereas detailed guides substantially increased agreement with the intended gold path. Together these results strengthen the RQ2 claim while defining it carefully: the AI component did not merely generate plausible text; it produced executable declarative structures when constrained by the schema, grounded in interface vocabulary, and given instructions specific enough for the desired procedure. However, to observe a certain behavior from the model, the instructions must include all the steps required to complete the task.

This capability opens the door to applications beyond the specific spreadsheet task evaluated here. If cognitive models can acquire task instructions from ordinary interface documentation, tutorials, prompts, or on-screen instructions, they can be used more flexibly for automated user-interface evaluation: a model could acquire the procedural structure of a task for a new interface, attempt it through the visible interface, and reveal where visual search becomes difficult, where instructions are ambiguous, where actions fail, or where the interface imposes unnecessary cognitive or motor burden. Such a framework could support comparative usability evaluation, accessibility assessment, intelligent tutoring systems, automated workflow testing, and interface redesign, and could be closed into a loop in which model output guides design changes that are then re-tested. This would not eliminate the need for human validation, but it could reduce reliance on repeated user recruitment during early design iterations. EVisiTor currently relies on UIA, which supports many applications but incompletely represents some environments, including terminals; general-purpose visual parsing is not yet reliable enough for arbitrary interfaces, although many widely used real-world applications do support UIA.

The empirical comparison between the realistic and perfect models shows the value of moving beyond idealized task execution. The EVisiTor-enabled realistic model reduced RMSE, human-SD-normalized RMSE, and mean absolute per-subtask error, and the complete factorial ablation showed that its three mechanism groups contribute near-additively, with one significant typing-side interaction between batched formula typing and the error-and-correction package. Visual search yielded a much closer approximation of participant variability on the visual-search-dominated subtasks. Formula-entry behavior remains far less variable than humans' and types more slowly (formula IKI 0.88 s vs. 0.70 s); the slowdown is almost entirely attributable to the look-back pathway, which by itself raises IKI from 0.37 s to 0.89 s, whereas perceptual checking and repair leave the interval essentially unchanged. The conclusion is therefore that EVisiTor can enable model processes that improve mean fit and process realism and begins to approximate participant to participant variability in completion time where visual search operates, while richer models of formula chunking, expertise, cognitive load, and checking are needed for participant-to-participant variability.

Overall, this work demonstrates that EVisiTor can extend cognitive architectures from idealized symbolic task models toward interactive agents that perceive, acquire task knowledge, act, make errors, and correct those errors in real software environments. More broadly, the work provides a methodological step toward AI-augmented cognitive simulation for interface evaluation, while identifying the next challenges: reliable visual parsing, formula-entry strategy and practice effects, and stronger modeling of individual differences.

## Data Availability

The datasets presented in this study can be found in online repositories. The names of the repository/repositories and accession number(s) can be found below: https://github.com/HCAI-Lab/EVisiTor_Code.
