# Supplementary material for: AI-augmented ACT-R modeling in real interfaces: automating visual perception, knowledge acquisition, and motor misexecution
Source: Front Psychol. 2026 Sep 16;17:1913953. doi: 10.3389/fpsyg.2026.1913953 (PMC13625840; doi:10.3389/fpsyg.2026.1913953)
Supplement: Supplementary file 1 [file Data_Sheet_1.pdf]

Supplementary Material

This supplementary file provides the implementation-level appendices supporting the ACT-R/EVisiTor model. The paper's primary contribution is the automation of live interface perception and instruction-based knowledge acquisition; the Excel behavioral analysis is a use case that exercises and showcases those capabilities. This file contains the lower-level model vocabulary, task-knowledge representation, prompts, instruction variants, gold-standard declarative structures, scoring and execution checks, configuration details, and the Task 7/9 sensitivity and ablation-design diagnostics.

Contents

Supplementary Appendix A. Exploratory eye-tracking analysis

Supplementary Appendix B. Detailed keystroke and typing-metric analysis

Supplementary Appendix C. ACT-R model implementation details

Supplementary Appendix D. LLM and VLM model reporting table

Supplementary Appendix E. Prompt templates for instruction-to-chunk conversion

Supplementary Appendix F. Original and paraphrased instruction sets

Supplementary Appendix G. Gold-standard declarative structures

Supplementary Appendix H. Raw LLM outputs and normalized generated structures

Supplementary Appendix I. Scoring procedure for instruction-to-chunk accuracy

Supplementary Appendix J. End-to-end execution check

Supplementary Appendix K. Model parameters, seeds, and batch configuration

Supplementary Appendix L. Sensitivity analyses: typing-error rates and formula-check probability

Supplementary Appendix M. Generalization probe: novel Windows tasks

Supplementary Appendix N. Exploratory vision-language-model perception

Supplementary Appendix O. Task 7/9 sensitivity and factorial ablation diagnostics

Supplementary Appendix P. Per-subtask and ablation-cell tables

Supplementary Appendix A. Exploratory Eye-Tracking Analysis

A limited subset of participants had videos with overlaid gaze indicators on top of them (n = 8 usable videos for the analyses reported below). These videos were post-processed to extract approximate gaze locations for exploratory behavioral interpretation. Because the original dataset did not report participant-level eye-tracking accuracy or complete data-loss information, these analyses are treated as qualitative and exploratory rather than as primary inferential evidence. They were used to ground the model assumptions about attention shifts during formula entry, word typing, cell search, and non-cell visual-object search. We then used the insight from the exploratory eye-tracking analysis and modeled different behaviors that were observed.

Gaze positions were extracted from the overlay videos automatically and analyzed with scripted areas of interest: the spreadsheet window (screen x < 800 px) and the instruction window (x > 1000 px), separated by a dead band so that fixations near the window boundary are not counted. An attention shift is a transition of the gaze point between the two areas of interest during a formula-entry segment (first "=" to the final Enter of the step). The coding was fully automated; no manual frame coding was performed. For this reason, and because only eight usable videos were available, we report the gaze statistics (including the attention-shift contrast below) as descriptive, exploratory evidence that motivated model assumptions; the model–human comparisons reported in the Results section do not depend on them.

A.1 Formula writing

Based on the recorded participants' interaction, participants shift back and forth between the formula in the instruction (Figure 2 in the main article, right window) and the location they are typing at (Figure 2 in the main article, left window). Depending on participants' expertise, the number of back-and-forths between the windows varies. More expert participants can fixate on the formula and type without looking back at the typing area, while less expert participants make more shifts of attention between the formula and the typing. Figure S1 showcases a participant’s eye movement in the formula typing process. The Figure shows 4 attention shifts between the instructions and the typing area.

A.2 Typing common words

Videos show that most participants (7 out of 8 available), once they knew what they needed to type, shifted their attention to the location of the typing bar and kept writing without looking back at the instructions. For more complex texts, such as “normalization-subject,” some participants shifted their attention back to the word, but these shifts were not as frequent as the attention shifts in steps where the participant needed to type a formula. Figure S2 highlights this eye movement in typing words.

A.3 Finding cells

In finding the first cell in a task such as “B7”, participants first searched for the column location, then shifted their attention to the row location, and finally found the cell at the intersection. In later steps (such as finding “B9”) some participants only looked for the changed element (here, the changed element in comparison to B7 or B8 is the row) and found the cell that matched the new row and the same column as the active cell. Figure S3 highlights the cell finding visual search.

A.4 Finding non-cell visual item locations

Some participants (who seemed less familiar with Excel) showed a top-down visual search strategy when finding the visual object they were looking for. These participants did not look around the cell area; instead, they seemed to search randomly across the screen for a text that matched what they were looking for.

Supplementary Appendix B. Detailed Keystroke and Typing-Metric Analysis

This appendix reports the detailed keystroke metrics summarized in the Typing Analysis section of the main text. All statistics use the N = 19 participants with usable keystroke logs; because some logs ended early, the number of contributing participants varies by step (Tables S1 and S2 give per-step contributors).

B.1 Formula-entry segments

A participant who completed the task has 35 expected formula-entry segments:

Step 3: 5 entries

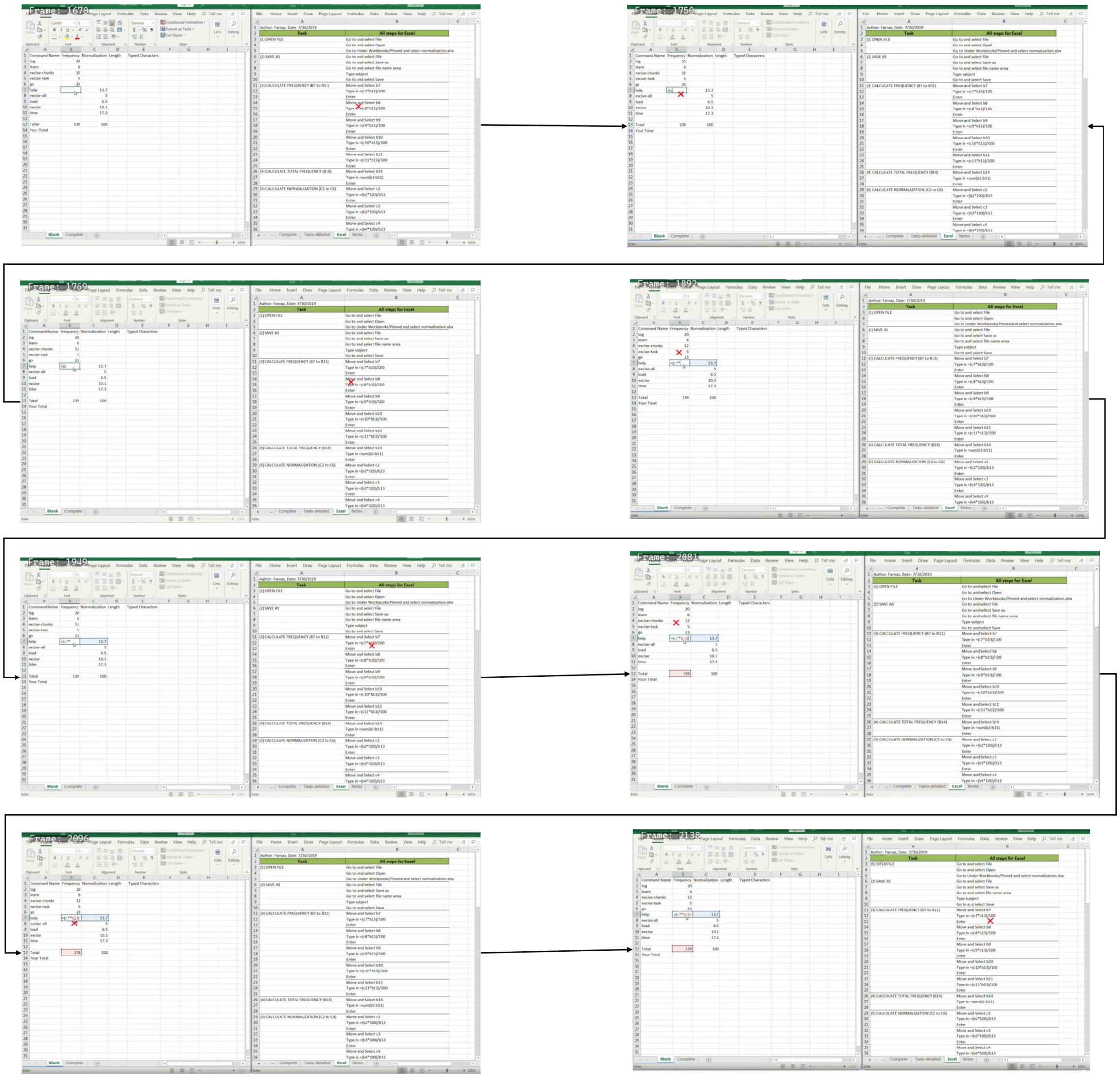

Figure S1: Participant eye movement and attention shifts during formula typing. This sequence of screenshots demonstrates visual attention shifting between the typing area in the left window and the task instructions containing the required formula in the right window. The red crosshairs show eye fixations over time, and the sequence illustrates four attention shifts between reading the formula and executing the input.

Step 4: 1 entry

Step 5: 5 entries

Step 6: 1 entry

Step 7: 10 entries

Step 8: 1 entry

Step 9: 10 entries

Step 10: 1 entry

Step 13: 1 entry

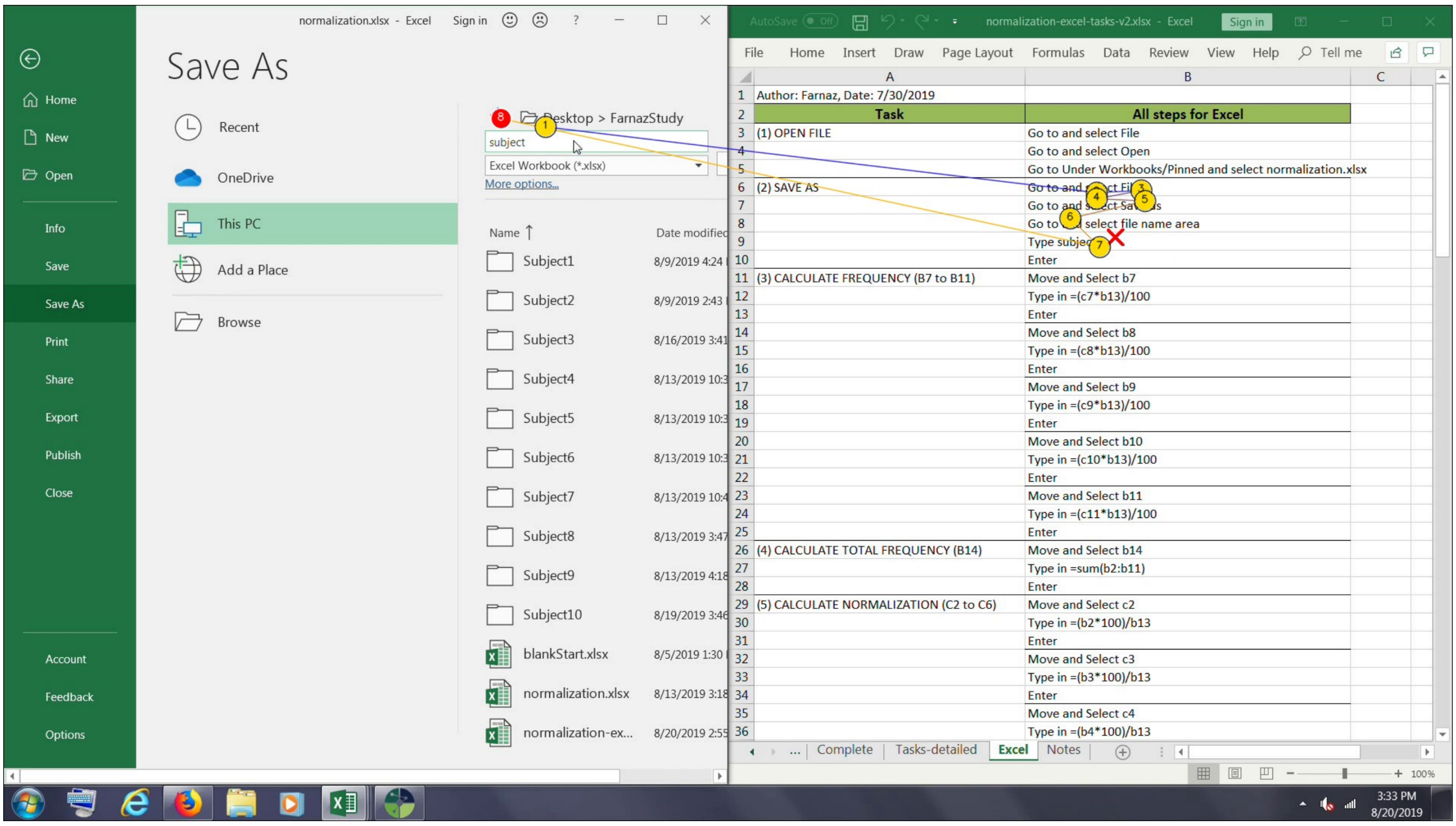

Figure S2: A participant's eye movement and attention shifts while typing common English words (“subject”). The overlaid gaze plot demonstrates that after locating the typing area (fixation 1), the participant first fixates on the text within the instructions in the right window (indicated by fixations 2 through 7) to read the required word. Fixation 8 shows that once they knew what they needed to type, they shifted their attention across the screen to the typing bar in the left window.

B.2 Correction and backspace metrics

Entries w/ Backspace is the number of formula entries where at least one Backspace occurred.

P(Observed Correction) is formula entries with at least one Backspace / observed formula entries

This is a probability-like estimate of how often users noticed something and corrected it during a formula entry.

Correction Episodes estimates typo/correction events. A correction episode is a run of one or more Backspace key presses. For example:

Backspace Backspace Backspace

counts as 1 correction episode and 3 Backspace presses. This is closer to "how many typo events happened" than raw Backspace count.

Backspaces is the raw number of Backspace keypresses.

Backspaces / Episode estimates how many characters users removed per correction event.

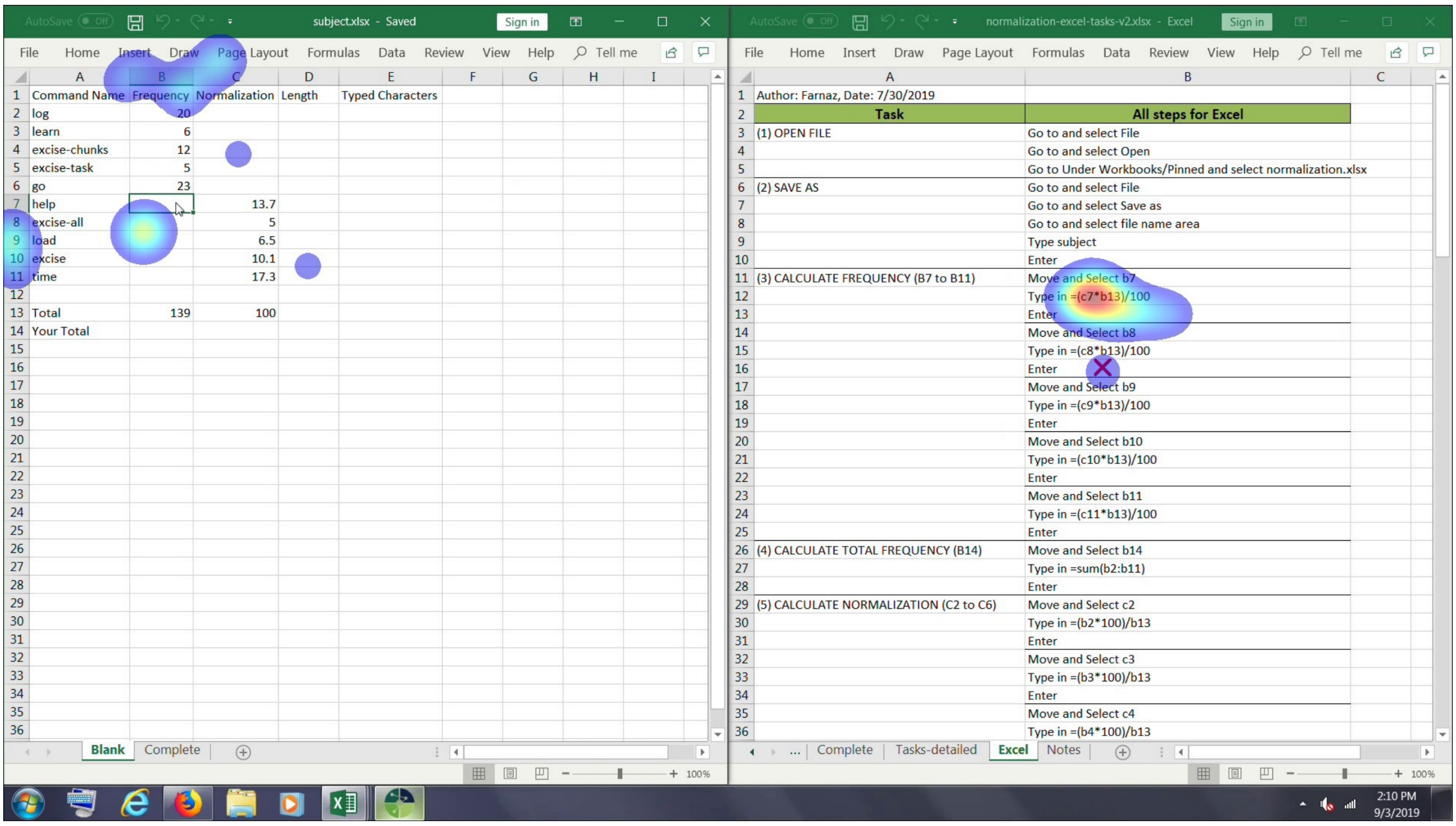

Figure S3: The gaze heat map highlights the visual search strategies employed by the participant for locating specific cells. In finding the initial cell, such as B7 referenced in the task list (Task 3: CALCULATE FREQUENCY), the gaze points on the column headers (around header B) illustrate the participant searching for the column location. The gaze then shifts to the row location, resulting in a large cluster of warm colors on the row header (around row 7), and then shifts attention to the intended cell, B7.

Table S1. Correction and backspace metrics by formula-entry task (the Participants column gives per-step contributors).

Task	Participants	Observed entries	Entries w/ Back-space	P(observed corre-ction)	Correction episodes	Backspaces	Backspaces / episode	Chars before correction
Step 3	19	95	28	29.5%	40	72	1.80	4.96
Step 4	19	19	6	31.6%	8	10	1.25	6.25
Step 5	19	95	29	30.5%	40	74	1.85	5.09
Step 6	19	19	4	21.1%	5	7	1.40	4.38
Step 7	19	190	45	23.7%	54	90	1.67	5.59
Step 8	18	18	7	38.9%	8	12	1.50	5.57
Step 9	18	174	33	19.0%	42	63	1.50	3.66
Step 10	16	16	5	31.2%	8	21	2.62	5.60
Step 13	13	13	3	23.1%	3	4	1.33	5.33

B.3 Within-task transition intervals

Table S2 reports pauses between consecutive formula entries inside the same task. Enter to next = is the time from submitting one formula to starting the next formula. Last char to next = includes the last visible typed character before Enter, the Enter key, and the transition to the next formula.

As shown in Table S2, Steps 3 and 5 produced substantially longer between-entry pauses than Steps 7 and 9. At the subject level, the mean Enter to next = transition was 9.55s for Steps 3/5 versus 4.75s for

Table S2. Within-task transition intervals between consecutive formula entries (19 participants with usable keystroke logs).

Task	Keypress task	Participants	Transitions	Avg Enter to next = (s)	Median Enter to next = (s)	Avg last char to next = (s)	Median last char to next = (s)
Step 3	Calculate frequency	19	76	11.69	6.30	13.64	7.96
Step 5	Calculate normalization	19	76	7.41	4.45	9.21	6.98
Step 7	Calculate length	19	171	4.56	2.51	5.44	3.54
Step 9	Calculate typed characters	18	156	4.25	2.11	5.33	3.16

Steps 7/9 (19 participants), paired t-test $p<0.001$, Wilcoxon $p<0.001$. The limited eye-tracking subset ($n = 8$) showed a consistent pattern: users made more left-right attention shifts during Steps 3/5 than Steps 7/9, 29.12 vs 17.81 shifts per formula-task window. Because only eight usable overlays were available, this contrast is reported descriptively and no inferential test is attached to it. This means that even though error making and error correction did not vary between formulas, the processing and encoding of the formulas did show an effect on typing speed, transition intervals, and the number of attention shifts.

B.4 Character-level correction summary

We suspected some characters that might be less used by the participants in everyday typing might have higher error and/or higher IKI. Less-used symbols include punctuation/operators such as =, /, :, *, (,), -, and +. Standard characters are letters and digits.

Operator-heavy formulas were slower: 0.765s mean IKI vs 0.606s for formulas without * or /, paired by subject $p<0.001$, with 16/19 subjects slower on operator-heavy formulas. Backspace error rates were not meaningfully different: 24.7% vs 25.5%, chi-square $p=0.9057$.

Supplementary Appendix C. ACT-R Model Implementation Details

This appendix preserves the lower-level ACT-R and VisiTor implementation details that were shortened in the main manuscript. The main text describes the architecture and mechanism-level contribution; this appendix reports the chunk structures, production-rule logic, visual-search implementation, typing pathways, motor-error implementation, logging streams, and baseline-model corrections needed for transparency and replication.

C.1 Baseline-model corrections

The perfect-user baseline follows the general task structure of the earlier Excel model developed by Tehranchi et al. (2023), but several implementation issues were corrected before comparison with the realistic VisiTor-enabled model. The corrected baseline includes attention shifts as part of the task routine, uses EMMA to simulate eye-movement timing, and corrects finger-position assumptions during typing. These corrections provide a cleaner baseline for estimating the additional contribution of VisiTor-enabled perception, motor-error production, and correction.

C.2 Declarative representations and chunk structure

The ACT-R model uses task-specific declarative chunks to represent the spreadsheet task, action meanings, target categories, remembered spatial locations, words, character sequences, and typing state. The primary chunk classes are summarized in Table S3.

Table S3. Primary declarative chunk classes used by the spreadsheet ACT-R model.

Chunk class	Role in the model
**task**	Represents the numbered spreadsheet tasks in the 14-task procedure.
**subtask and subtaskX_Y_list**	Represent ordered instruction structures within each task and subtask.
**actionencode**	Maps human-readable actions such as Goto, Select, Type, and Press into internal action meanings, including attention-and-cursor movement, clicking, typing, and pressing Enter.
**target-spec**	Stores target-category information used during visual search, including whether a target is a spreadsheet cell, row, column, or ordinary non-cell visual object.
**remembered-location**	Stores locations already found by the model, including item identity and spatial coordinates; these chunks allow later operations on the same or nearby interface objects to reuse spatial memory.
**word and item**	Represent words, formulas, and their character-level or token-level components.
**typing**	Stores the current text-entry state, including the word or formula being typed, current token or character, position, next position, and whether an error position has been detected.

C.3 Production-rule sequence for task execution

The spreadsheet model advances through tasks using a recurring production-rule sequence. It retrieves the pending task, creates a visual query for the task marker, and starts VisiTor logging for that task. The model then moves into a task-attention state, attends to the visible task marker, repeats the attention process for the current subtask, retrieves the next action-item pair from the relevant subtask list, maps the action through actionencode, and executes the appropriate visual-search, motor-action, or typing pathway.

This sequence matters because the model does not begin by directly manipulating the target object. It first attends to the instruction region, retrieves the current declarative structure, interprets the requested action, and only then acts in the interface. Thus, the action list supplied through knowledge acquisition is task knowledge that must be retrieved, interpreted, and executed through ACT-R buffers and production rules rather than a direct script.

C.4 Visual-search implementation

Non-cell targets, such as File Tab, Open, Save a Copy, Home, and Insert Cells, are treated as ordinary interface objects. The model retrieves a target-spec chunk, checks whether a remembered-location chunk is available, and, if available, searches near that remembered position. If no remembered position is available, the model chooses between local and global visual search. Local search requests an unattended non-cell text object near the current attentional locus; global search requests an eligible unattended non-cell text

object without the local-neighborhood constraint. Candidate objects are attended and compared with the target until the correct item is found.

Cell targets are treated separately because spreadsheet cells have row-column structure. A target such as B7 is decomposed into a column label and a row label. The model checks whether the highlighted cell already matches the target; otherwise, it retrieves or searches for the column header, retrieves or searches for the row header, and then requests the visual location at their intersection. The resulting target-cell location is encoded in remembered-location memory for later reuse.

The visual-perception bridge can use screenshot-based template matching for pre-cropped areas of interest and structured Microsoft UI Automation output for live interface extraction. The UIA pathway serializes visible interface elements and converts them into ACT-R-compatible visicon features, including location, size, label/value information, cell status, row/column status, and highlight state. ACT-R then uses ordinary visual-location requests over these features rather than receiving target positions directly.

C.5 Typing, formula entry, and correction logic

When the current instruction is Type, the model retrieves a word or formula representation and selects the appropriate typing pathway. Ordinary words are typed character by character. Before each character is sent, the motor-error mechanism may substitute a plausible wrong key. The bridge writes the delivered character to the live interface and updates a typed-echo visual feature at the typing location. Intended and delivered keys are retained for audit logs, but their equality is not exposed to ACT-R as an error signal.

The ordinary word pathway includes immediate and delayed perceptual checking. When a check is selected (probability 0.85 in the reported realistic condition), ACT-R requests the typed-echo visual location, moves attention to it, incurs visual encoding time, reads the encoded character, and compares that percept with the intended character. A perceived mismatch initiates Backspace/Delete repair and retyping. If an immediate word check is skipped, the stored uncertainty is realized at a final-character check of the completed word, which uses the same overt attend-and-encode chain before the word can finish. Thus correction depends on an overt perceptual chain rather than on direct access to the bridge's intended/delivered log.

Formula typing uses a separate pathway because users often alternate attention between the instruction source and the target cell while entering formula components. The tokenizer groups formula components such as alphabetic sequences, numeric sequences, cell references, and symbols into manageable tokens. The typing goal tracks the token index, character index, current token, target cell, and formula source. After a token is completed, the model shifts attention back to the instruction source, obtains the next token, and returns to the target cell. This loop continues until the formula is complete.

Formula and learned-formula entries are checked probabilistically through the same perceptual chain, but the unit of checking is the token rather than the individual keystroke. When formula typing is token-batched (B=1), the model returns attention to the formula source after each completed nonfinal token; a check rides along at that boundary, and each such boundary draws once (probability 0.90 in the reported configuration) whether the accumulated entry is verified overtly - attend the typed-echo feature, encode the on-screen text, compare it with the intended characters - or left unverified at that moment. A slip is therefore detected at most one token after it is made. When a formula is typed without batching (B=0) there are no token boundaries, so no per-keystroke or per-token check probability applies during typing: the formula is typed straight through and detection falls entirely to the entry-level check. Token boundaries whose draw skips the overt check, and the final token of every formula, are not verified in progress; any surviving typo is realized

at a guaranteed final check that always precedes Enter. Before submitting an entry the model shifts attention to the typed text, encodes it, and compares the tracked entry with the intended formula; a dirty entry is repaired by deleting back to the first wrong character and retyping the tail, with the repair keystrokes passing through the same typo injection, and the read-back repeats until the entry passes. Check-driven attention shifts are logged with path-specific sources (word-immediate, word-final, formula-token, and formula-final variants of typed-text-check), and the direct escape-hatch productions that bypass the overt chain (retained only as deadlock protection) fired zero times in the reported runs.

Characters that require Shift are represented explicitly as a Shift action followed by the base key. This keeps the ACT-R motor sequence explicit for symbols such as parentheses and formula operators rather than treating shifted symbols as atomic characters.

C.6 EVisiTor motor bridge, error sampling, and logging

The EVisiTor motor bridge monitors ACT-R manual commands, sends them to a Python server through a persistent TCP connection, and executes them through operating-system-level keyboard and mouse functions. The implementation records the intended key for logging, samples whether a character-level motor typo should occur, stores the delivered key separately, and uses only the delivered key to update the live interface and typed-echo visual feature. Protected command keys, including Enter, Shift, Backspace, Delete, Tab, arrows, Ctrl, and Alt, are excluded from character-level typo injection in the reported model.

For typo-eligible characters, the wrong key can be sampled from an empirical conditional probability table when available; otherwise, EVisiTor uses plausible adjacent or nearby substitutions. Shifted characters are handled by tracking pending Shift state and mapping the base key to the shifted output. After delivery, ACT-R cannot query a zero-time intended-versus-delivered comparison. Correction productions can respond only after the visual system locates and encodes the typed-echo feature. Conditions without correction checking disable this perceptual check chain.

EVisiTor logs attention shifts, keypresses, subtask times, task times, error events, correction events, and visual-search events. Attention logs include the run ID, model time, task, subtask, attended location, coordinates, and source; check-driven shifts to the typed text are path-labelled. In the reported build the model keeps the entry field attended and re-encodes it automatically as characters arrive, so checking does not generate a separate typed-text-check shift per keystroke: the full-realistic cell logs about 1,701 attention shifts per run, of which 38 are explicit shifts to the typing area (one per entry) preceding the guaranteed pre-Enter read-back. Keypress logs include intended and delivered keys for audit, while model behavior depends on the perceptually encoded echo. Batch execution reloads the model for each run, stores run-level logs in separate directories, and writes a per-run condition manifest (the actual state of every mechanism switch plus the run's seed) and a post-run check recording that the perceptual chain remained active throughout the run.

C.7 Reproducibility details

The reported spreadsheet batch used ACT-R 7.x, Python 3.12, Windows 11, Excel version 2604, and the local EVisiTor UI Automation/TCP bridge. The eight factorial cells (142 valid runs each, 1,136 runs total) are archived under Spreadsheet/logs/draft36_check090_factorial (22 July 2026), together with SHA-256 hashes of the frozen model sources, the archived 142-element seed list applied in the same order to every cell (seeding both the host random stream and ACT-R's :seed parameter), and the per-run condition manifests. Supplementary Appendix K records the model files, parameters, batch provenance, and analysis configuration needed to interpret those results.

Supplementary Appendix D. LLM and VLM Model Reporting Table

The Knowledge-Acquisition Accuracy section reports six evaluated instruction-to-chunk models. All six were accessed through the same OpenAI-compatible client with identical decoding settings: temperature 0.2, top-p 0.95, maximum 512 output tokens, no streaming. Outputs were generated in a single pass per instruction variant, with no retries and no manual repair before scoring. The GPT-OSS-120b entry is dated 08/05/2025 because it was an early pilot evaluation performed with the model's public release build (released 5 August 2025; OpenAI, 2025), before the 2026 runs of the other models; it was retained because gpt-oss-120b remains one of the most widely used open-weight baselines. For the original spreadsheet evaluation, 18 calls were represented (six models × three instruction variants): the structured response payloads were archived verbatim as returned, before normalization, while the corresponding request objects were reconstructed deterministically from the pinned evaluation script and disclose that provenance in their _provenance field. The reconstructed requests record the endpoint, the full prompt, and the strict JSON-schema response format. The original HTTP envelopes — response IDs, token usage, finish reasons, and system fingerprints — were not persisted, so the historical calls cannot be replayed exactly against these evolving hosted endpoints, and the reported accuracies should be read as snapshots of the accessed versions. The prompts, instruction variants, gold structures, and normalized structures with their ACT-R translations are archived (Appendices E–H, and appendix_h_llm_calls/). Complete raw request/response JSON was archived for every model call of the later Windows generalization-probe evaluation (Appendix M). Supplementary Appendix H gives the full account of what was and was not retained.

The lower rows of Table S4 list the systems evaluated for visual-interface perception. The adopted backend is the Microsoft UI Automation plus VisiTor visicon converter. Before adopting it, we also tested a dedicated screenshot-based GUI parser and several general-purpose vision-language models as candidate perception backends; these attempts are reported in Supplementary Appendix N and are listed here for completeness. The hosted vision systems (GPT-5.5 and Gemini 3.1 Pro) were accessed through their respective APIs with structured-JSON output, and the open-weight models (EGM-8B, Qwen3-VL-30B-A3B, and Gemma 4 31B) were served with vLLM on Google Colab (A100 GPU) using grammar-constrained JSON decoding. Each was prompted to return task-relevant interface objects as labels with bounding boxes; none reached the spatial precision required for direct use as ACT-R visicon entries, and none was adopted.

Table S4. Model and backend reporting information for instruction-to-chunk conversion and visual-interface extraction.

Model/backend	Provider or route	Role	Access/decoding notes
GPT-5.5	OpenAI API	Instruction-to-chunk conversion	Recorded date: 05/28/2026.
GPT-5.4-mini	OpenAI API	Instruction-to-chunk conversion	Recorded date: 2026-03-17.
GPT-5.4-nano	OpenAI API	Instruction-to-chunk conversion	Recorded date: 2026-03-17.
Gemini-3.5-Flash	Google Gemini endpoint	Instruction-to-chunk conversion and execution-equivalence check	Recorded date: 05/2026.
Gemini-3.1-Flash-Lite	Google Gemini endpoint	Instruction-to-chunk conversion	Recorded date: 05/2026.

Model/backend	Provider or route	Role	Access/decoding notes
openai/GPT-OSS-120b	OpenRouter-hosted open-weight model	Instruction-to-chunk conversion	Recorded date: 08/05/2025.
DeepSeek-V4-Flash	Hosted open-weight model	Attempted but excluded	Not evaluated because of free-tier rate limiting on the hosting service; it was, however, evaluated in the Windows-task generalization-probe instruction-parsing analysis (Appendix M).
Nemotron-3-Super	Hosted open-weight model	Attempted but excluded	Not evaluated because of free-tier rate limiting on the hosting service; it was, however, evaluated in the Windows-task generalization-probe instruction-parsing analysis (Appendix M).
Microsoft UI Automation plus VisiTor visicon converter	Local Windows UIA, Python/TCP bridge, and ACT-R visual-location interface	Visual parsing and visicon extraction	Used for live interface feature extraction rather than instruction-to-chunk generation.
OmniParser V2	Local screenshot-based GUI parser (Microsoft OmniParser V2)	Exploratory visual-interface perception (screenshot parsing)	Appendix N.1; not adopted (adjacent cells merged, column headers and window controls dropped). Ref: Yu et al., 2026.
GPT-5.5 (vision parsing)	OpenAI API	Exploratory visual-interface perception	Appendix N.2 (OpenAI_VisionParser.py); structured-JSON output; plausible object labels but bounding boxes not precise enough for visicon coordinates.

Model/backend	Provider or route	Role	Access/decoding notes
Gemini 3.1 Pro	Google Gemini API	Exploratory visual-interface perception	Appendix N.2-N.3 (Gemini_VisionParser.py); served checkpoint gemini-3.1-pro-preview; structured-JSON output at temperature 0.0; best-performing parser tested, but incomplete and unstable across cascading-menu states. Ref: DeepMind, 2026a.
EGM-8B	Open-weight VLM (vLLM on Google Colab, A100 GPU)	Exploratory visual-interface perception	Appendix N.2 (run_egm.py); checkpoint nvidia/EGM-8B-SFT; grammar-constrained JSON decoding; did not reliably localize task-relevant objects.
Qwen3-VL-30B-A3B	Open-weight VLM (vLLM on Google Colab, A100 GPU)	Exploratory visual-interface perception	Appendix N.2 (run_qwen3vl.py); served checkpoint Qwen/Qwen3-VL-30B-A3B-Instruct; grammar-constrained JSON decoding; did not reliably localize task-relevant objects. Ref: Qwen, 2026.
Gemma 4 31B	Open-weight VLM (vLLM on Google Colab, A100 GPU)	Exploratory visual-interface perception	Appendix N.2 (run_gemma4.py); checkpoint google/gemma-4-31b-it; grammar-constrained JSON decoding; most detailed output among the small models but localization still imprecise. Ref: DeepMind, 2026b.

Supplementary Appendix E. Prompt Templates for Instruction-to-Chunk Conversion

The instruction parser used a constrained prompt that asked the model to generate only declarative Subtask chunks. The language model was not asked to write ACT-R production rules or modify the cognitive model. The schema, action vocabulary, and protected naming rules were supplied by the modeler.

System/context prompt: You are an ACT-R expert. You need to only give the users what they want without any explanation.

User prompt template: I am writing an ACT-R model. I want you to break down the following sentence and only generate chunks for the chunk-type {chunk_type}. Based on the following prompt {prompt}. Remember you can create as many chunks as needed and every action needs an object. An object might be

shared between two verbs. Chunks are defined as follows: (define-chunks ("this is chunk e" isa chunk-type feature value)).

Chunk type supplied to the model: (chunk-type Subtask actions items).

Expected raw output form: (define-chunks (chunk-name isa Subtask action VERB item TARGET) ...). No prose, markdown, commentary, alternate schema, or production rules were allowed.

Normalized executable form: (setq subtaskT_S_list '((Verb "Target") (Verb "Target") (end|endsub))).

Action vocabulary: Goto, Move, Select, Type, and Press. Goto or Move map to attention-and-cursor movement, Select maps to click, Type maps to text entry, and Press maps to keyboard command entry.

Schema constraints: Every action must have exactly one object; shared objects are repeated as separate action-item pairs; action order is preserved; each subtask closes with end or endsub; no extra chunks are accepted during scoring.

Protected naming constraints: Preserve exact target labels and spreadsheet references. Required literal labels include File Tab, Save a Copy, Insert Cells, Open, Home, normalization.xlsx, file_name, Row_1, subject, normalization-subject, and Excel cell/formula strings. Row_1 must remain Row_1 because the ACT-R/UIA lookup maps that protected label to the row-header object. File Tab must retain its capitalization and space; file_name must retain lowercase spelling and underscore.

Supplementary Appendix F. Original and Paraphrased Instruction Sets

The original instruction sequence and two semantics-preserving paraphrases (Table S5) were used to evaluate the robustness of instruction-to-chunk conversion. Protected interface labels and spreadsheet targets were kept unchanged so that errors could be attributed to instruction parsing rather than to unsupported UI naming variants.

Table S5. Original task instructions and paraphrased variants were used for robustness testing.

Task	Original instruction	Paraphrase 1	Paraphrase 2
1	Open the File Tab, choose Open, and open normalization.xlsx.	Use File Tab to open normalization.xlsx from Open.	Go to File Tab, pick Open, then choose normalization.xlsx.
2	Open the File Tab, choose Save a Copy, select file_name, type subject, and press enter.	Save a copy from File Tab, activate file_name, enter subject, and confirm with enter.	Go through File Tab and Save a Copy, click file_name, type subject, then press enter.
3	In B7:B11, calculate the frequencies using formulas =(C7*B13)/100 through =(C11*B13)/100.	Fill B7 through B11 with the corresponding frequency formulas from =(C7*B13)/100 to =(C11*B13)/100.	Compute the lower frequency cells B7, B8, B9, B10, and B11 with their matching C-row formulas divided by B13 and multiplied by 100.
4	In B14, calculate the total frequency using =sum(B2:B11).	Put =sum(B2:B11) in B14 for the frequency total.	Compute B14 as the sum of B2 through B11.

Task	Original instruction	Paraphrase 1	Paraphrase 2
5	In C2:C6, calculate normalization using formulas =(B2*100)/B13 through =(B6*100)/B13.	Fill C2 through C6 with the matching normalization formulas from =(B2*100)/B13 to =(B6*100)/B13.	Compute C2, C3, C4, C5, and C6 with the corresponding B-row values times 100 over B13.
6	In C14, calculate the total normalization using =sum(C2:C11).	Put =sum(C2:C11) in C14 for total normalization.	Compute C14 as the sum of C2 through C11.
7	In D2:D11, calculate word length using =len(A2) through =len(A11).	Enter =len(A2) through =len(A11) in D2 through D11.	Compute D2 through D11 as the length of the text in A2 through A11.
8	In D14, calculate the total length using =sum(D2:D11).	Put =sum(D2:D11) in D14.	Compute D14 as the sum of D2 through D11.
9	In E2:E11, calculate typed characters using =D2*B2 through =D11*B11.	Enter the typed-character formulas =D2*B2 through =D11*B11 in E2 through E11.	Compute E2 through E11 by multiplying each D-row value by the corresponding B-row value.
10	In E14, calculate total typed characters using =sum(E2:E11).	Put =sum(E2:E11) in E14.	Compute E14 as the sum of E2 through E11.
11	Insert two rows at Row_1 by selecting Row_1, Home, and Insert Cells twice.	Insert Row_1 twice by using Row_1, Home, and Insert Cells for each insertion.	Select Row_1 and use Home then Insert Cells; repeat the same Row_1 insertion once more.
12	In A1, type subject and press enter.	Select A1, type subject, and press enter.	Put subject in A1 and press enter.
13	In A2, type =today() and press enter.	Select A2, type =today(), and press enter.	Put =today() in A2 and press enter.
14	Open File Tab, choose Save a Copy, select file_name, type normalization-subject, and press enter.	Save another copy from File Tab by selecting file_name, typing normalization-subject, and pressing enter.	Use File Tab and Save a Copy, select file_name, type normalization-subject, and press enter.

Supplementary Appendix G. Gold-Standard Declarative Structures

The executable gold-standard reference used for scoring and execution contains 94 named subtask lists and 187 non-terminal atomic action items. The representation below is read directly from GOLD_LISP in EnvironmentParsers/InstructionAcquisitionParser.py; terminal markers end and endsub are not counted as actions.

G.1 Hand-authored subtask-list chunks

The following code block gives the normalized executable subtask-list representation.

```
(setq subtask1_1_list '((Goto "File Tab") (Select "File Tab") (end)))
```

```
(setq subtask1_2_list '((Goto "open") (Select "open") (end)))
(setq subtask1_3_list '((Goto "normalization") (Select "normalization") (endsub)))
(setq subtask2_1_list '((Goto "File Tab") (Select "File Tab") (end)))
(setq subtask2_2_list '((Goto "Save a Copy") (Select "Save a Copy") (end)))
(setq subtask2_3_list '((Goto "file_name") (Select "file_name") (end)))
(setq subtask2_4_list '((Type "subject") (end)))
(setq subtask2_5_list '((Goto "Save") (Select "Save") (endsub)))
(setq subtask3_1_list '((Goto "B7") (Select "B7") (end)))
(setq subtask3_2_list '((Type "=(c7*b13)/100") (Press "enter") (end)))
(setq subtask3_3_list '((Goto "B8") (Select "B8") (end)))
(setq subtask3_4_list '((Type "=(c8*b13)/100") (Press "enter") (end)))
(setq subtask3_5_list '((Goto "B9") (Select "B9") (end)))
(setq subtask3_6_list '((Type "=(c9*b13)/100") (Press "enter") (end)))
(setq subtask3_7_list '((Goto "B10") (Select "B10") (end)))
(setq subtask3_8_list '((Type "=(c10*b13)/100") (Press "enter") (end)))
(setq subtask3_9_list '((Goto "B11") (Select "B11") (end)))
(setq subtask3_10_list '((Type "=(c11*b13)/100") (Press "enter") (endsub)))
(setq subtask4_1_list '((Goto "B14") (Select "B14") (end)))
(setq subtask4_2_list '((Type "=sum(b2:b11)") (Press "enter") (endsub)))
(setq subtask5_1_list '((Goto "C2") (Select "C2") (end)))
(setq subtask5_2_list '((Type "=(b2*100)/b13") (Press "enter") (end)))
(setq subtask5_3_list '((Goto "C3") (Select "C3") (end)))
(setq subtask5_4_list '((Type "=(b3*100)/b13") (Press "enter") (end)))
(setq subtask5_5_list '((Goto "C4") (Select "C4") (end)))
(setq subtask5_6_list '((Type "=(b4*100)/b13") (Press "enter") (end)))
(setq subtask5_7_list '((Goto "C5") (Select "C5") (end)))
(setq subtask5_8_list '((Type "=(b5*100)/b13") (Press "enter") (end)))
(setq subtask5_9_list '((Goto "C6") (Select "C6") (end)))
(setq subtask5_10_list '((Type "=(b6*100)/b13") (Press "enter") (endsub)))
(setq subtask6_1_list '((Goto "c14") (Select "c14") (end)))
(setq subtask6_2_list '((Type "=sum(c2:c11)") (Press enter) (endsub)))
(setq subtask7_1_list '((Goto "D2") (Select "D2") (end)))
(setq subtask7_2_list '((Type "=len(a2)") (Press enter) (end)))
```

```
(setq subtask7_3_list '((Goto "d3") (Select "d3") (end)))
(setq subtask7_4_list '((Type "=len(a3)") (Press enter) (end)))
(setq subtask7_5_list '((Goto "d4") (Select "d4") (end)))
(setq subtask7_6_list '((Type "=len(a4)") (Press enter) (end)))
(setq subtask7_7_list '((Goto "d5") (Select "d5") (end)))
(setq subtask7_8_list '((Type "=len(a5)") (Press enter) (end)))
(setq subtask7_9_list '((Goto "d6") (Select "d6") (end)))
(setq subtask7_10_list '((Type "=len(a6)") (Press enter) (end)))
(setq subtask7_11_list '((Goto "d7") (Select "d7") (end)))
(setq subtask7_12_list '((Type "=len(a7)") (Press enter) (end)))
(setq subtask7_13_list '((Goto "d8") (Select "d8") (end)))
(setq subtask7_14_list '((Type "=len(a8)") (Press enter) (end)))
(setq subtask7_15_list '((Goto "d9") (Select "d9") (end)))
(setq subtask7_16_list '((Type "=len(a9)") (Press enter) (end)))
(setq subtask7_17_list '((Goto "d10") (Select "d10") (end)))
(setq subtask7_18_list '((Type "=len(a10)") (Press enter) (end)))
(setq subtask7_19_list '((Goto "d11") (Select "d11") (end)))
(setq subtask7_20_list '((Type "=len(a11)") (Press enter) (endsub)))
(setq subtask8_1_list '((Goto "d14") (Select "d14") (end)))
(setq subtask8_2_list '((Type "=sum(d2:d11)") (Press enter) (endsub)))
(setq subtask9_1_list '((Goto "e2") (Select "e2") (end)))
(setq subtask9_2_list '((Type "=d2*b2") (Press enter) (end)))
(setq subtask9_3_list '((Goto "e3") (Select "e3") (end)))
(setq subtask9_4_list '((Type "=d3*b3") (Press enter) (end)))
(setq subtask9_5_list '((Goto "e4") (Select "e4") (end)))
(setq subtask9_6_list '((Type "=d4*b4") (Press enter) (end)))
(setq subtask9_7_list '((Goto "e5") (Select "e5") (end)))
(setq subtask9_8_list '((Type "=d5*b5") (Press enter) (end)))
(setq subtask9_9_list '((Goto "e6") (Select "e6") (end)))
(setq subtask9_10_list '((Type "=d6*b6") (Press enter) (end)))
(setq subtask9_11_list '((Goto "e7") (Select "e7") (end)))
(setq subtask9_12_list '((Type "=d7*b7") (Press enter) (end)))
(setq subtask9_13_list '((Goto "e8") (Select "e8") (end)))
```

(setq subtask9_14_list '((Type "=d8*b8") (Press enter) (end)))

(setq subtask9_15_list '((Goto "e9") (Select "e9") (end)))

(setq subtask9_16_list '((Type "=d9*b9") (Press enter) (end)))

(setq subtask9_17_list '((Goto "e10") (Select "e10") (end)))

(setq subtask9_18_list '((Type "=d10*b10") (Press enter) (end)))

(setq subtask9_19_list '((Goto "e11") (Select "e11") (end)))

(setq subtask9_20_list '((Type "=d11*b11") (Press enter) (endsub)))

(setq subtask10_1_list '((Move "e14") (Select "e14") (end)))

(setq subtask10_2_list '((Type "=sum(e2:e11)") (Press enter) (endsub)))

(setq subtask11_1_list '((Goto "Row_1") (Select "Row_1") (end)))

(setq subtask11_2_list '((Goto "Home") (Select "Home") (end)))

(setq subtask11_3_list '((Goto "Cells") (Select "Cells") (end)))

(setq subtask11_4_list '((Goto "Insert") (Select "Insert") (end)))

(setq subtask11_5_list '((Goto "Insert-cell") (Select "Insert-cell") (end)))

(setq subtask11_6_list '((Goto "Row_1") (Select "Row_1") (end)))

(setq subtask11_7_list '((Goto "Home") (Select "Home") (end)))

(setq subtask11_8_list '((Goto "Cells") (Select "Cells") (end)))

(setq subtask11_9_list '((Goto "Insert") (Select "Insert") (end)))

(setq subtask11_10_list '((Goto "Insert-cell") (Select "Insert-cell") (endsub)))

(setq subtask12_1_list '((Goto "A1") (Select "A1") (end)))

(setq subtask12_2_list '((Type "subject") (Press enter) (endsub)))

(setq subtask13_1_list '((Goto "A2") (Select "A2") (end)))

(setq subtask13_2_list '((Type "=today()") (Press enter) (endsub)))

(setq subtask14_1_list '((Goto "File Tab") (Select "File Tab") (end)))

(setq subtask14_2_list '((Goto "Save") (Select "Save") (end)))

(setq subtask14_3_list '((Goto "file_name") (Select "file_name") (end)))

(setq subtask14_4_list '((Type "normalization-subject") (Press enter) (endsub)))

G.2 Atomic action items derived from the hand-authored chunks

Table S6 expands the 94 subtask lists into 187 atomic action items, excluding terminal markers end and endsub.

Table S6. Atomic action items from the gold-standard subtask-list representation.

Subtask list	Action	Target
subtask1_1_list	Goto	File Tab
subtask1_1_list	Select	File Tab
subtask1_2_list	Goto	open
subtask1_2_list	Select	open
subtask1_3_list	Goto	normalization
subtask1_3_list	Select	normalization
subtask2_1_list	Goto	File Tab
subtask2_1_list	Select	File Tab
subtask2_2_list	Goto	Save a Copy
subtask2_2_list	Select	Save a Copy
subtask2_3_list	Goto	file_name
subtask2_3_list	Select	file_name
subtask2_4_list	Type	subject
subtask2_5_list	Goto	Save
subtask2_5_list	Select	Save
subtask3_1_list	Goto	B7
subtask3_1_list	Select	B7
subtask3_2_list	Type	=(c7*b13)/100
subtask3_2_list	Press	enter
subtask3_3_list	Goto	B8
subtask3_3_list	Select	B8
subtask3_4_list	Type	=(c8*b13)/100
subtask3_4_list	Press	enter
subtask3_5_list	Goto	B9
subtask3_5_list	Select	B9
subtask3_6_list	Type	=(c9*b13)/100
subtask3_6_list	Press	enter
subtask3_7_list	Goto	B10
subtask3_7_list	Select	B10
subtask3_8_list	Type	=(c10*b13)/100
subtask3_8_list	Press	enter
subtask3_9_list	Goto	B11
subtask3_9_list	Select	B11
subtask3_10_list	Type	=(c11*b13)/100
subtask3_10_list	Press	enter
subtask4_1_list	Goto	B14
subtask4_1_list	Select	B14
subtask4_2_list	Type	=sum(b2:b11)
subtask4_2_list	Press	enter
subtask5_1_list	Goto	C2
subtask5_1_list	Select	C2
subtask5_2_list	Type	=(b2*100)/b13
subtask5_2_list	Press	enter
subtask5_3_list	Goto	C3

Subtask list	Action	Target
subtask5_3_list	Select	C3
subtask5_4_list	Type	=(b3*100)/b13
subtask5_4_list	Press	enter
subtask5_5_list	Goto	C4
subtask5_5_list	Select	C4
subtask5_6_list	Type	=(b4*100)/b13
subtask5_6_list	Press	enter
subtask5_7_list	Goto	C5
subtask5_7_list	Select	C5
subtask5_8_list	Type	=(b5*100)/b13
subtask5_8_list	Press	enter
subtask5_9_list	Goto	C6
subtask5_9_list	Select	C6
subtask5_10_list	Type	=(b6*100)/b13
subtask5_10_list	Press	enter
subtask6_1_list	Goto	c14
subtask6_1_list	Select	c14
subtask6_2_list	Type	=sum(c2:c11)
subtask6_2_list	Press	enter
subtask7_1_list	Goto	D2
subtask7_1_list	Select	D2
subtask7_2_list	Type	=len(a2)
subtask7_2_list	Press	enter
subtask7_3_list	Goto	d3
subtask7_3_list	Select	d3
subtask7_4_list	Type	=len(a3)
subtask7_4_list	Press	enter
subtask7_5_list	Goto	d4
subtask7_5_list	Select	d4
subtask7_6_list	Type	=len(a4)
subtask7_6_list	Press	enter
subtask7_7_list	Goto	d5
subtask7_7_list	Select	d5
subtask7_8_list	Type	=len(a5)
subtask7_8_list	Press	enter
subtask7_9_list	Goto	d6
subtask7_9_list	Select	d6
subtask7_10_list	Type	=len(a6)
subtask7_10_list	Press	enter
subtask7_11_list	Goto	d7
subtask7_11_list	Select	d7
subtask7_12_list	Type	=len(a7)
subtask7_12_list	Press	enter
subtask7_13_list	Goto	d8

Subtask list	Action	Target
subtask7_13_list	Select	d8
subtask7_14_list	Type	=len(a8)
subtask7_14_list	Press	enter
subtask7_15_list	Goto	d9
subtask7_15_list	Select	d9
subtask7_16_list	Type	=len(a9)
subtask7_16_list	Press	enter
subtask7_17_list	Goto	d10
subtask7_17_list	Select	d10
subtask7_18_list	Type	=len(a10)
subtask7_18_list	Press	enter
subtask7_19_list	Goto	d11
subtask7_19_list	Select	d11
subtask7_20_list	Type	=len(a11)
subtask7_20_list	Press	enter
subtask8_1_list	Goto	d14
subtask8_1_list	Select	d14
subtask8_2_list	Type	=sum(d2:d11)
subtask8_2_list	Press	enter
subtask9_1_list	Goto	e2
subtask9_1_list	Select	e2
subtask9_2_list	Type	=d2*b2
subtask9_2_list	Press	enter
subtask9_3_list	Goto	e3
subtask9_3_list	Select	e3
subtask9_4_list	Type	=d3*b3
subtask9_4_list	Press	enter
subtask9_5_list	Goto	e4
subtask9_5_list	Select	e4
subtask9_6_list	Type	=d4*b4
subtask9_6_list	Press	enter
subtask9_7_list	Goto	e5
subtask9_7_list	Select	e5
subtask9_8_list	Type	=d5*b5
subtask9_8_list	Press	enter
subtask9_9_list	Goto	e6
subtask9_9_list	Select	e6
subtask9_10_list	Type	=d6*b6
subtask9_10_list	Press	enter
subtask9_11_list	Goto	e7
subtask9_11_list	Select	e7
subtask9_12_list	Type	=d7*b7
subtask9_12_list	Press	enter
subtask9_13_list	Goto	e8

Subtask list	Action	Target
subtask9_13_list	Select	e8
subtask9_14_list	Type	=d8*b8
subtask9_14_list	Press	enter
subtask9_15_list	Goto	e9
subtask9_15_list	Select	e9
subtask9_16_list	Type	=d9*b9
subtask9_16_list	Press	enter
subtask9_17_list	Goto	e10
subtask9_17_list	Select	e10
subtask9_18_list	Type	=d10*b10
subtask9_18_list	Press	enter
subtask9_19_list	Goto	e11
subtask9_19_list	Select	e11
subtask9_20_list	Type	=d11*b11
subtask9_20_list	Press	enter
subtask10_1_list	Move	e14
subtask10_1_list	Select	e14
subtask10_2_list	Type	=sum(e2:e11)
subtask10_2_list	Press	enter
subtask11_1_list	Goto	Row_1
subtask11_1_list	Select	Row_1
subtask11_2_list	Goto	Home
subtask11_2_list	Select	Home
subtask11_3_list	Goto	Cells
subtask11_3_list	Select	Cells
subtask11_4_list	Goto	Insert
subtask11_4_list	Select	Insert
subtask11_5_list	Goto	Insert-cell
subtask11_5_list	Select	Insert-cell
subtask11_6_list	Goto	Row_1
subtask11_6_list	Select	Row_1
subtask11_7_list	Goto	Home
subtask11_7_list	Select	Home
subtask11_8_list	Goto	Cells
subtask11_8_list	Select	Cells
subtask11_9_list	Goto	Insert
subtask11_9_list	Select	Insert
subtask11_10_list	Goto	Insert-cell
subtask11_10_list	Select	Insert-cell
subtask12_1_list	Goto	A1
subtask12_1_list	Select	A1
subtask12_2_list	Type	subject
subtask12_2_list	Press	enter
subtask13_1_list	Goto	A2

Subtask list	Action	Target
subtask13_1_list	Select	A2
subtask13_2_list	Type	=today()
subtask13_2_list	Press	enter
subtask14_1_list	Goto	File Tab
subtask14_1_list	Select	File Tab
subtask14_2_list	Goto	Save
subtask14_2_list	Select	Save
subtask14_3_list	Goto	file_name
subtask14_3_list	Select	file_name
subtask14_4_list	Type	normalization-subject
subtask14_4_list	Press	enter

Supplementary Appendix H. Raw LLM Outputs and Normalized Generated Structures

Raw request and response JSON for the six evaluated instruction-to-chunk models is archived in appendix_h_llm_calls/ in the submission package: 18 calls (six models × three instruction variants) under the grounding condition reported in Table 6 of the main text, indexed by manifest.json. Each responses/<model>__<variant>.response.json file is the model's structured output exactly as returned, before the normalization applied for scoring; these files are verbatim copies of the payloads persisted by the evaluation script and are also distributed in generated_chunks/ alongside their ACT-R .lisp translations. Each requests/<model>__<variant>.request.json file records the endpoint, the full prompt, and the strict JSON-schema response format for that call; these request objects are reconstructed deterministically from the pinned evaluation script rather than captured from the wire, and each states this in its _provenance field. What was not persisted, and cannot be recovered, is the HTTP envelope of each historical call — response id, token usage, finish reason, and system fingerprint — so the recorded calls cannot be replayed against these evolving hosted endpoints. The normalized subtask-list representation used for scoring and execution is given in Supplementary Appendix G.

For the generalization-probe evaluation (Appendix M), complete raw request/response JSON was archived for every model call (llm_parsing_eval/ in the repository, one file per model x instruction variant x grounding condition), addressing the archival gap of the original spreadsheet evaluation.

Gemini-generated structures were considered executable after normalization only when their ordered action-target pairs and protected UI names matched the gold representation. Table S7 records the prompt and output evidence available for each model together with the normalized structure used for scoring.

Supplementary Appendix I. Scoring Procedure for Instruction-to-Chunk Accuracy

Generated instruction-to-chunk structures were scored against the gold-standard representation in Supplementary Appendix G using the criteria below; Table S8 reports the resulting per-model accuracy.

Schema validity: A response is valid only if it can be parsed into the supplied Subtask schema, contains action-item pairs, uses the allowed action vocabulary, assigns one target to each action, and contains no extra prose or unsupported fields.

Table S7. Available prompt/output evidence and normalized structure used for scoring and execution.

Item	Example or status	Use in the manuscript
Prompt example	chunk_type = "(chunk-type Subtask actions items)"; prompt = "Go to and select Open"	Documents the constrained instruction-to-chunk conversion format.
Raw LLM output example	(define-chunks (move isa Subtask action move item Open) (select isa Subtask action select item Open))	Illustrates the raw chunk form before normalization.
Normalized generated structure	(setq subtask1_2_list '((Goto "Open") (Select "Open") (end)))	Executable representation used for scoring and ACT-R execution.
Raw JSON archive	appendix_h_llm_calls/ — 18 request/response files (six models × three instruction variants) plus manifest.json.	Responses are verbatim as returned; requests are reconstructed from the pinned evaluation script. The HTTP envelope (id, usage, finish reason) was not persisted, so the historical calls cannot be replayed.

Action-verb accuracy: Each generated verb is mapped case-insensitively to the canonical vocabulary Goto, Move, Select, Type, or Press. Semantic substitutions such as click for Select are accepted only when the normalization map explicitly defines the equivalence.

Target accuracy: Target strings must match the gold target after allowed whitespace cleanup and protected-name normalization. Spreadsheet cell references and formulas must match exactly, including row numbers, column letters, arithmetic operators, parentheses, and ranges.

Naming normalization: Trim surrounding whitespace; normalize verb case to canonical spelling; preserve target case for UI labels; map row one, row 1, and first row to Row_1 only in contexts that explicitly refer to row insertion; preserve File Tab, Save a Copy, Insert Cells, file_name, and normalization.xlsx as exact protected labels; preserve formulas exactly except for harmless outer whitespace.

Score reporting: Report schema-valid count, subtask coverage, action-verb accuracy, target accuracy, exact-match rate, false positives, false negatives, and protected-name violations.

Table S8. Instruction-to-chunk conversion accuracy by model.

Model	Provider	Valid Schema	Action acc.	Target orig.	Target P1	Target P2
GPT-5.5	OpenAI	100%	99.5%	90.3%	96.8%	89.8%
Gemini-3.5-Flash	Google	100%	99.5%	94.6%	92.5%	96.8%
GPT-5.4-mini	OpenAI	100%	76.6%	90.3%	88.2%	10.7%
GPT-5.4-nano	OpenAI	100%	68.9%	89.9%	86.0%	10.6%
Gemini-3.1-Flash-Lite	Google	100%	77.4%	91.4%	92.5%	12.8%
GPT-OSS-120b	OpenRouter	100%	78.2%	90.3%	88.2%	17.7%

Supplementary Appendix J. End-to-End Execution Check: Modeler-Defined vs Gemini-Generated Chunks

Gemini-generated instruction structures were normalized into the same executable subtask-list representation as the modeler-defined chunks. The equivalence check compared each subtask name, ordered action verb, ordered target, and terminal marker against the gold reference.

Downstream behavior is identical when the normalized Gemini-generated list is structurally identical to the modeler-defined list for all executable action-target pairs, because the ACT-R model reads only the normalized subtask lists during execution. Under this criterion, equal normalized lists force the same retrieved actions and targets, leaving all downstream timing and error processes controlled by the same model parameters. Table S9 reports the check for each generated subtask list.

Table S9. End-to-end execution check for modeler-defined and Gemini-generated structures.

Source	Constraints	Status	Outcome
Gold-standard chunks	modeler-defined; UIA-compatible names	Successful	Reference execution
Gemini-generated chunks	Schema prompt with Row_1 and File Tab	Successful	Equivalent structures; identical downstream performance

Supplementary Appendix K. Model Parameters, Seeds, and Batch Configuration

This appendix summarizes the simulation environment, ACT-R parameters, typing and checking parameters, visual-search parameters, and instruction-parser API defaults used for the spreadsheet model; Table S10 gives the full summary.

Table S10. Model environment and configuration summary.

Category	Configuration details
**ACT-R and EVisiTor environment**	**Local ACT-R 7.x integration with Windows UI Automation and a Python/TCP motor bridge; Windows 11, Python 3.12, and Excel 2604.**

Table S10. Model environment and configuration summary. (Continued)

Category	Configuration details
**Spreadsheet model and ablation files**	**Spreadsheet/Complete_error_model_tcp.lisp loads New_Error (3-5-2-TCP-7-21-2026).lisp, running_model_v4.lisp, fucntion_new_tcp.lisp (sic; literal filename), and Ablation_support.lisp; apply-spreadsheet-factorial in Ablation_support.lisp configures the three factors independently. The eight cells are defined by Ablation_Perfect.lisp (000), Ablation_Visual_Search_Only.lisp (100), Ablation_Batched_Formula_Typing.lisp (010), Ablation_Correction_Checking.lisp (001), Ablation_Visual_Plus_Batched.lisp (110), Ablation_Visual_Plus_Checking.lisp (101), Ablation_Batched_Plus_Checking.lisp (011), and Ablation_Full_Realistic.lisp (111). Each cell loads the base model named by the SPREADSHEET_ABLATION_BASE_MODEL environment variable; the reported batch set it to New_Error (3-5-2-TCP-7-21-2026).lisp, whose SHA-256 is recorded in analysis/batch_provenance/draft36_check090_factorial/source_hashes.json.**
**ACT-R parameters from the realistic model source**	**:v t; :needs-mouse t; :show-focus t; :trace-detail high; :emma t; :TT 3; :MAS 1.6; :BLL 0.1; :RT 0; :LF 0.05; :LE 1.0; :BLC 5.0; :ANS 0.005; :MD −1.0; :DECLARATIVE-NUM-FINSTS 4; :DECLARATIVE-FINST-SPAN 3.0; :OL T; :ESC T; :ER NIL; :EGS 0.1; :NU 20; :UL T; :ALPHA 0.2; :IU 10; :VISUAL-NUM-FINSTS 1000; :VISUAL-FINST-SPAN 1000.**
**Typing and perceptual-checking parameters**	**C=1 cells: formula content-key typo rate 0.034 and word content-key typo rate 0.063; formula per-token visual-check probability 0.90, applied at each formula look-back and therefore only in the token-batched (B=1) cells; immediate word-check probability 0.85; guaranteed pre-Enter read-back enabled in every C=1 cell. C=0 cells: both typo rates 0.0 and every checking/repair mechanism disabled. The checking path locates and encodes a typed-echo visual feature; no direct intended-versus-delivered error signal is available to ACT-R.**
**Visual-search parameters**	**Local-neighbor search probability 0.9; global-search probability 0.1 when the probabilistic branch chooses non-local search.**

Table S10. Model environment and configuration summary. (Continued)

Category	Configuration details
**Spreadsheet batch and uncertainty analysis**	**Spreadsheet/logs/draft36_check090_factorial (2026-07-22); 142 valid runs in each of the eight factorial cells (1,136 runs), one archived 142-element seed list applied identically to every cell, SHA-256 source freeze, and per-run condition manifests. The factorial bootstrap resampled runs within each cell (10,000 resamples); the primary task bootstrap and the participant/run Wasserstein bootstrap each used 10,000 resamples. The human distributional analysis used 21 participants with complete subtask windows; analysis seed 20260720.**
**Instruction-parser API parameters**	**VLLM_URL defaults to http://127.0.0.1:8000/v1/chat/completions; VLLM_MODEL defaults to Xingyu-Zheng/gemma-4-E2B-it-int4-foem; VLLM_TIMEOUT_SECONDS defaults to 120; requests use temperature 0.2, top_p 0.95, max_tokens 512, and stream false unless overridden.**

Supplementary Appendix L. Sensitivity Analyses: Typing-Error Rates and Formula-Check Probability

This appendix reports how the model's fit responds to the three free quantities that govern typing errors and their detection: the formula typo rate, the word typo rate, and the probability that a completed formula token is visually checked. All three sweeps were run with visual search disabled and formula typing token-batched (cell 011: V=0, B=1, C=1), the configuration in which the per-token check is active, so that the reported spread reflects the manipulated quantity rather than visual-search variance, and all use the same model build and archived 142-seed sequence as the main factorial. Because visual search contributes roughly 86 s of task time, the absolute completion times and RMSE values here are not comparable to the full realistic model in the main text; only the shape of each curve should be read.

L.1 Independent typing-error-rate sweeps

The formula and word typo rates were varied one at a time from 0 % to 5 % in 1 % steps, 142 runs per condition (12 conditions, 1 704 runs). When one rate was varied, the other was held at its calibrated human value (word 6.3 %, formula 3.4 %). Table S11 reports the formula and word typing-error-rate sweeps, including the manipulated and held-constant rates, mean total completion time, RMSE with 95 % bootstrap confidence intervals, the pooled correction probability, and the realized formula-entry keystroke error rate. Figure S4 plots per-subtask RMSE against each manipulated rate. RMSE compares the 14 simulated subtask means with the 14 human means; 95 % intervals come from 5000 run-level bootstrap resamples.

The two rates behave very differently. Raising the formula typo rate from 0 % to 5 % lengthens total time by about 27 s (628.1 s to 655.1 s) and worsens RMSE by 1.0 s (20.68 s to 21.67 s), with non-overlapping confidence intervals at the extremes. Raising the word typo rate over the same range moves total time by 2.8 s and RMSE by 0.08 s, and every interval overlaps every other, so that curve is flat within noise. The correction measure locates the operating point directly: pooled P(correction) rises from 1.0 % at a nominal

Table S11. Independent formula and word typing-error-rate sweeps for cell 011, with 142 runs per condition. The table reports the manipulated and held-constant rates, mean total completion time, RMSE with 95 % bootstrap confidence intervals, the pooled probability that a formula entry contains at least one correction, and the realized error rate over formula-entry content keystrokes. P(correction) uses the same first-"=" to Enter segmentation as the human 25.0 % value, so the two are directly comparable.

Sweep	Varied rate (%)	Formula rate (%)	Word rate (%)	Mean total (s)	RMSE (s) [95 % CI]	P(correction) (%)	Realized formula error rate (%)
formula	0	0.0	6.3	628.1	20.68 [20.65, 20.72]	1.0	0.13
formula	1	1.0	6.3	633.1	20.82 [20.76, 20.87]	10.0	1.10
formula	2	2.0	6.3	638.4	21.01 [20.94, 21.08]	17.9	2.08
formula	3	3.0	6.3	643.6	21.21 [21.12, 21.30]	25.3	3.01
formula	4	4.0	6.3	648.9	21.49 [21.38, 21.60]	31.7	3.99
formula	5	5.0	6.3	655.1	21.67 [21.56, 21.77]	38.7	5.08
word	0	3.4	0.0	642.1	21.41 [21.32, 21.50]	27.2	3.29
word	1	3.4	1.0	642.5	21.38 [21.30, 21.48]	27.2	3.30
word	2	3.4	2.0	643.4	21.37 [21.29, 21.46]	27.5	3.34
word	3	3.4	3.0	644.0	21.35 [21.26, 21.44]	27.6	3.35
word	4	3.4	4.0	644.5	21.34 [21.25, 21.44]	27.8	3.37
word	5	3.4	5.0	644.9	21.33 [21.24, 21.42]	27.8	3.39

0 % formula rate to 38.7 % at 5 %, crossing the human 25.0 % value at the 3 % condition (25.3 %), that is, at the independently measured human formula rate of 3.35 % that the shipped model uses. Varying the word rate over the same range moves P(correction) only from 27.2 % to 27.8 %, because the model types roughly nine times as many formula as word keystrokes. The realized rates confirm that the manipulation was delivered as intended (0.13 % to 5.08 % across the formula sweep). The asymmetry follows from the task rather than from any fitted quantity: formulas are long, densely delimited strings entered under look-back, so an error in one triggers far more repair work than a mistyped word. These are sensitivity results, not parameter fitting; the calibrated rates were fixed in advance from the human keystroke data and were retained even though they do not minimise RMSE.

L.2 Formula-check-probability sweep

The probability that a completed formula token is visually checked at its look-back boundary was varied from 0.0 to 1.0 in steps of 0.1 (11 conditions, 142 runs each, 1 562 runs). Typing-error rates were held at their calibrated values (formula 3.4 %, word 6.3 %), so checking probability is the only quantity that differs between conditions. Each run executed subtasks 1 through 10; subtasks 1 and 2 establish the workbook state that later subtasks edit and are excluded from scoring, so all fit statistics below are computed over subtasks 3-10 only (Table S12).

The fit is largely insensitive to this parameter. RMSE varies by less than 1 s across the entire grid (19.59 s at $p = 0.0$ down to 18.64 s at $p = 0.5$), and the 95 % confidence interval of the nominally best condition overlaps those of all conditions from $p = 0.2$ to $p = 1.0$. The value used throughout the paper, $p = 0.90$, yields 18.66 s and is statistically indistinguishable from the nominal minimum. The deviation measures also disagree in direction: RMSE mildly favours more checking, whereas mean absolute error and correlation both favour little or no in-progress checking (MAE is lowest at $p = 0.1$, 11.13 s, and essentially tied at $p = 0.0$, 11.16 s, against 12.11 s at $p = 0.90$; $r = 0.961$ at $p = 0.0$). We therefore do not treat any value on this grid as a fitted estimate, and retain the a priori 0.90. We report the full curve rather than an argmin precisely because a minimum read off a flat curve would overstate what these data constrain.

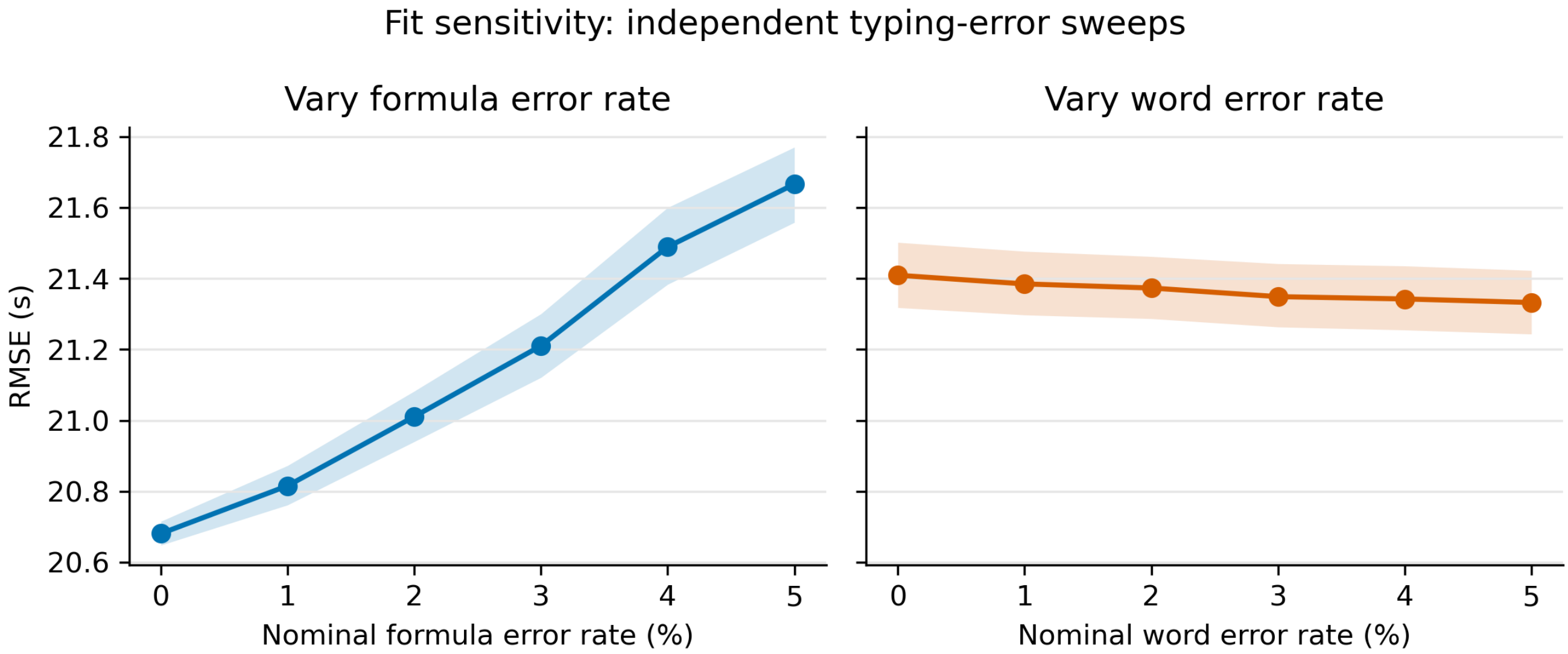

Figure S4: Sensitivity of model–human fit to the two typing-error rates (142 runs per condition, visual search disabled so that its run-to-run variance cannot mask the manipulation). Left: the formula error rate varied from 0 % to 5 % with the word rate held at 6.3 %. Right: the word rate varied over the same range with the formula rate held at 3.4 %. Each panel shows per-subtask RMSE against the 14 human means; shaded bands are 95 % bootstrap intervals.

Table S12. Formula-check-probability sweep (cell 011, subtasks 3-10 scored, 142 runs per condition).

Check probability	Scored total, tasks 3-10 (s)	RMSE (s) [95 % CI]	MAE (s)
0.0	596.7	19.59 [19.17, 20.04]	11.16
0.1	590.5	19.31 [18.91, 19.74]	11.13
0.2	585.0	19.17 [18.82, 19.52]	11.50
0.3	580.4	18.95 [18.63, 19.30]	11.79
0.4	577.7	18.88 [18.59, 19.16]	11.94
0.5	574.9	18.64 [18.39, 18.89]	12.02
0.6	574.1	18.76 [18.53, 18.97]	12.18
0.7	574.2	18.66 [18.46, 18.86]	12.09
0.8	573.9	18.66 [18.48, 18.83]	12.09
0.9	573.9	18.66 [18.49, 18.84]	12.11
1.0	574.8	18.73 [18.57, 18.89]	12.11

Provenance. Rate sweeps: Spreadsheet/logs/draft36_novisual_rate_sweeps_check090 (Ablation_NoVisual_Rates.lisp). Check-probability sweep: Spreadsheet/logs/draft34_20260722_checkprob_sweep (Ablation_NoVisual_CheckProb.lisp, Run_CheckProb_Shard.lisp). Both roots contain a design_manifest.json recording the SHA-256 of every model source, the seed list, the held-constant rates, and the runs per condition; both were verified to hold exactly 142 completed runs in every condition.

Supplementary Appendix M. Generalization Probe: Novel Windows Tasks

This appendix documents the generalization probe reported in the main text. Tasks were drawn from the public Paradigm Shift AI computer-use dataset (Howland et al., 2025; MIT license), which contains recorded human executions of everyday computer tasks: screen video, timestamped input events, and vendor-written step descriptions. From its 142 Windows-native recordings we initially selected ten tasks; six

were retained (Table S13). Exclusions and reasons: changing the screen resolution (the demonstration skips an unstated confirmation dialog), ending a process in Task Manager (the instruction names no resolvable target process), recording an Excel macro (unstated data range and scope), and reading ipconfig output in a terminal (UI Automation exposes no text elements inside the console, so the perception pathway does not apply; this exclusion marks a boundary of UIA-based perception).

Table S13. The six retained Windows tasks with their PSAI dataset identifiers.

ID	Task	Interface context	PSAI unique_data_id
W01	Open Notepad via the Run dialog	Run dialog (Win+R)	cmaela4ed001gzr0xhfhqg921
W02	Rename budget.xlsx to budget_2025.xlsx	File Explorer command bar	cmaela4el00dezr0xar3kk07l
W03	Create folder 'Projects'	File Explorer/Desktop; Ctrl+Shift+N	cm9egbfne005d0e0v3a0rw2pb
W04	Compress 'Logs' to a zipped folder	Two-tier context menu (Show more options > Send to)	cmaela4el00dgzr0xvj1od7q9
W09	Open Calculator via Start-menu search	Taskbar search	cma092ib400ghwu0v52t3qxia
W10	Find report.docx in Documents	File Explorer search box	cm9egbkl7005j0e0v774e8iqr

For each retained task, we hand-authored one gold decomposition of the method shown in the recorded demonstration, using the paper's action schema extended with RightClick and Scroll. Target labels were reconciled with the UIA output available to EVisiTor, and the chunks were validated through live execution. The six gold decompositions contain 21 subtasks and 39 atomic actions. The generic task-independent model retains only primitive interaction skills from the realistic spreadsheet model; every Excel-specific production was removed. The gold is an executable reference path, not a claim that no other Windows strategy is correct.

Broad-instruction conversion scoring. Seven scorable models converted the dataset task sentences into chunks. Exact matching compares the generated and gold flat action sequences, independent of subtask segmentation. Because these instructions often permit several valid strategies, a deterministic functional-equivalence check separately judged whether each generated sequence would complete the task from its actual start state. Complete prompts, raw responses, aligned comparisons, and verdicts are archived in the repository workbook.

UIA-grounded prompting. Each broad instruction was tested without UIA context and with full UIA grounding, where the prompt included the union of element names captured during the live task. UIA grounding supplies interface vocabulary, but it does not uniquely determine which valid procedure the model should choose.

Live execution. The generic model, driven by the gold chunks, completed all six tasks in the live Windows 11 interface (Table S21). Completion was verified by system state: Notepad and Calculator launched, budget_2025.xlsx renamed on disk, the Projects folder created, a valid Logs.zip produced, and report.docx located. A lightweight run-time resolver mapped chunk target names to live UIA labels when exact matching failed (for example, Rename to "Rename (F2)"); all mappings were cached and logged. Two bridge-level fixes were applied globally during the live sessions (re-scanning the interface after clicks that open cascading menus, and completing pending motor actions before halting); no task-specific tuning

was performed. Screen recordings of the model runs, the resolver transcript, and per-task completion logs (task_times.csv) are archived in the repository. Human reference times are the span from first to last recorded event of the single vendor demonstration of each task; they are single observations, not samples, and no model parameter was adjusted toward them.

M.1 UIA-grounded and guided parsing: a proof of concept for automating model perception and action specification

Following the initial probe, the instruction-to-chunk evaluation was re-run (July 2026) with three refinements, as a proof of concept that UI Automation and LLMs together can automate what the cognitive model perceives (the visicon is built from UIA scans) and what actions it takes (task chunks are generated from natural-language instructions). Complete inputs, outputs, per-step scans, comparisons, and verdicts are archived in llm_parsing_eval/W6_model_IO_and_gold_2026-07-16.xlsx; the evaluation code is EnvironmentParsers/UIAGroundedParser.py, run_perstep.py, build_w6_excel.py, and functional_check.py.

Gold corrections. The decompositions were corrected for start-state consistency and perceptual groundability: W03 was revised from the demonstrated right-click on the folder view's empty background to the Ctrl+Shift+N chord: the unoccupied region is not a UIA visual item (no scan exposes a 'Desktop background' element; the empty view's only accessible text is 'This folder is empty.'), so a gold step anchored on it can never be grounded, whereas the chord (or the command-bar New > Folder entry) references only real, scannable objects and is reproduced perfectly. The corrected gold has 21 subtasks and 39 atomic actions.

Schema fix. The Windows evaluation sends a seven-verb schema, extending the five-verb spreadsheet vocabulary (Goto, Move, Select, Type, Press) with RightClick and Scroll, because the retained tasks require context menus and scrolled file lists that the spreadsheet vocabulary cannot express. Scoring is otherwise unchanged from Supplementary Appendix I.

Conditions and models. Seven models were scorable: GPT-5.5, GPT-5.4-mini, GPT-5.4-nano, Gemini-3.1-Flash-Lite, GPT-OSS-120b, DeepSeek-V4-Flash, and Nemotron-3-Super; Gemini-3.5-Flash remained unavailable because of a provider outage. Two grounding conditions are reported: no-UIA (no element list) and full-UIA (the union of live UIA scans). Two instruction forms were tested: the dataset sentence and a detailed guide containing one step per gold subtask. Functional equivalence is reported only for the broad-instruction form. The guide explicitly specifies the intended method, so guide results are compared only with the gold sequence.

Detailed guide instructions used in the guide condition

The following text is reproduced verbatim from the guide inputs used in the evaluation.

W01 — Open Notepad through the Run dialog

1. Press Win+R to open the Run dialog.
2. Type 'notepad' and press Enter.

W02 — Rename budget.xlsx

1. Click the File Explorer icon on the taskbar to open File Explorer.
2. Click the file 'budget.xlsx' in the file list.
3. Click the Rename button in the toolbar.

4. Type 'budget_2025.xlsx' and press Enter.

W03 — Create the Projects folder

1. Click the File Explorer icon on the taskbar to open File Explorer.
2. Click 'Desktop' in the navigation pane.
3. Press Ctrl+Shift+N to create a new folder.
4. Type 'Projects' and press Enter.

W04 — Compress the Logs folder

1. Right-click the folder 'Logs'.
2. Click 'Show more options' in the context menu.
3. Click 'Send to', then click 'Compressed (zipped) folder' in the submenu that opens.
4. Press Enter to accept the default archive name.

W09 — Open Calculator

1. Click the Search box on the taskbar.
2. Type 'calculator' and press Enter.
3. Look at the Calculator window that opened.

W10 — Search for report.docx

1. File Explorer is already open. Click 'Documents' in the navigation pane.
2. Click the search box labeled 'Search Documents'.
3. Type 'report.docx' and press Enter.
4. Look at the search result 'report.docx'.

Supplementary Appendix N. Exploratory Vision-Language-Model Perception

EVisiTor perceives the interface through UI Automation and application-specific extractors. Before settling on that design, we tested whether general-purpose vision-language models (VLMs) and screenshot-based GUI parsers could serve directly as the perception backend, taking a screenshot and returning labelled, localized objects that could be written into the ACT-R visicon. This appendix reports those attempts. The outcome was a negative result: none of the parsers we tried met the requirement that task-critical objects (cells, row and column headers, menu items, dialog fields, and dynamically appearing overlays) be recovered with enough spatial precision for ACT-R visual-location requests. We report it because it is the reason the framework uses accessibility metadata for spatial grounding, and because the failure modes are specific enough to be informative. The evaluation criterion here is not aggregate object-detection accuracy, which can be high while the few objects the model actually needs are the ones that are missed. Model and backend identifiers for every system named below are listed in Supplementary Appendix D; the prompt templates follow the pattern in Supplementary Appendix E.

N.1 Screenshot-based GUI parsing (OmniParser V2)

We first applied OmniParser V2 (Yu et al., 2026), a dedicated screen-parsing model, to several states of the Dismal spreadsheet task. It recovered much of the interface, but the errors fell on exactly the objects the model needs (Figure S5): adjacent spreadsheet cells were merged into a single detection, column headers were dropped, and window controls were missed. A merged cell pair is not a small localization error for a cognitive model - it removes a candidate the visual-location request is searching for, so the model either fails to find its target or attends to the wrong one.

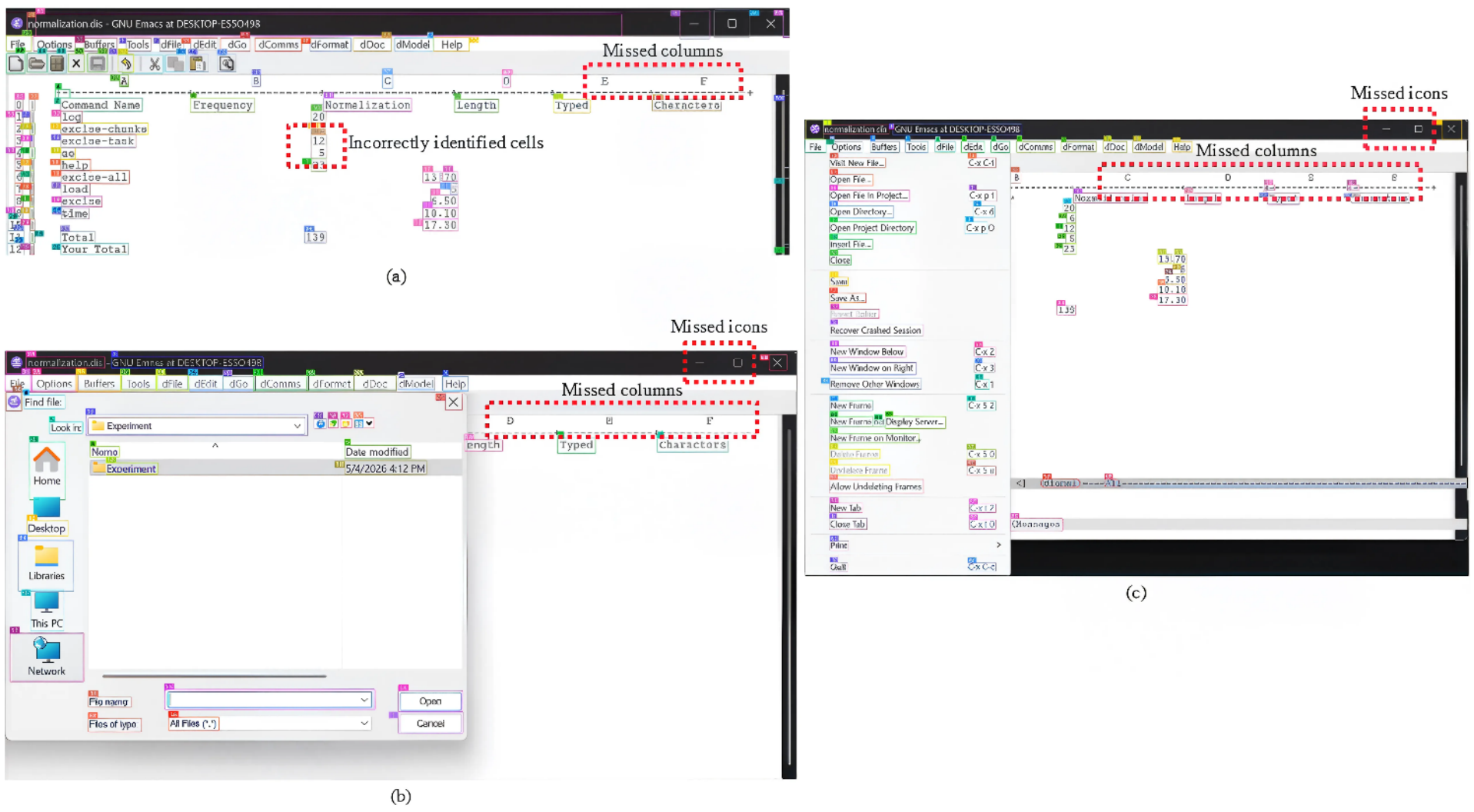

Figure S5: OmniParser V2 visual object detection in different stages of the spreadsheet task in Dismal. In (a), the model incorrectly merged two cells (B3 and B4) and detected them as one visual object, and did not detect two column headers (E and F). In (b), the model missed two icons (minimize and maximize icons) and missed three columns. In (c) the model missed two icons and 4 columns.

N.2 General-purpose vision-language models

We then compared general-purpose VLMs on a single fixed Dismal interface state, prompting each to return task-relevant objects as labels with bounding boxes (Figure S6). Semantic labelling and spatial grounding dissociated clearly. The frontier models produced plausible, often correct names for what was on screen, but their bounding boxes were not accurate enough to be written into the visicon as coordinates. The smaller open-weight models were weaker on both dimensions.

N.3 Stability across interface states

Because the model must perceive the interface continuously rather than once, we applied the best-performing model from N.2 to four Dismal states spanning the range the task actually visits (Figure S7). Performance degraded with clutter and occlusion. Simple workspace views were parsed reasonably well; overlapping and cascading menu states produced missed objects, merged boxes, and hallucinated labels.

N.4 Interpretation and consequences for the architecture

These errors are consequential for cognitive simulation in a way they would not be for an interface-automation agent. An incorrect or missing visual object can alter the model's search path, send attention to the wrong location, or produce an invalid action, and it does so silently: the model has no way to tell

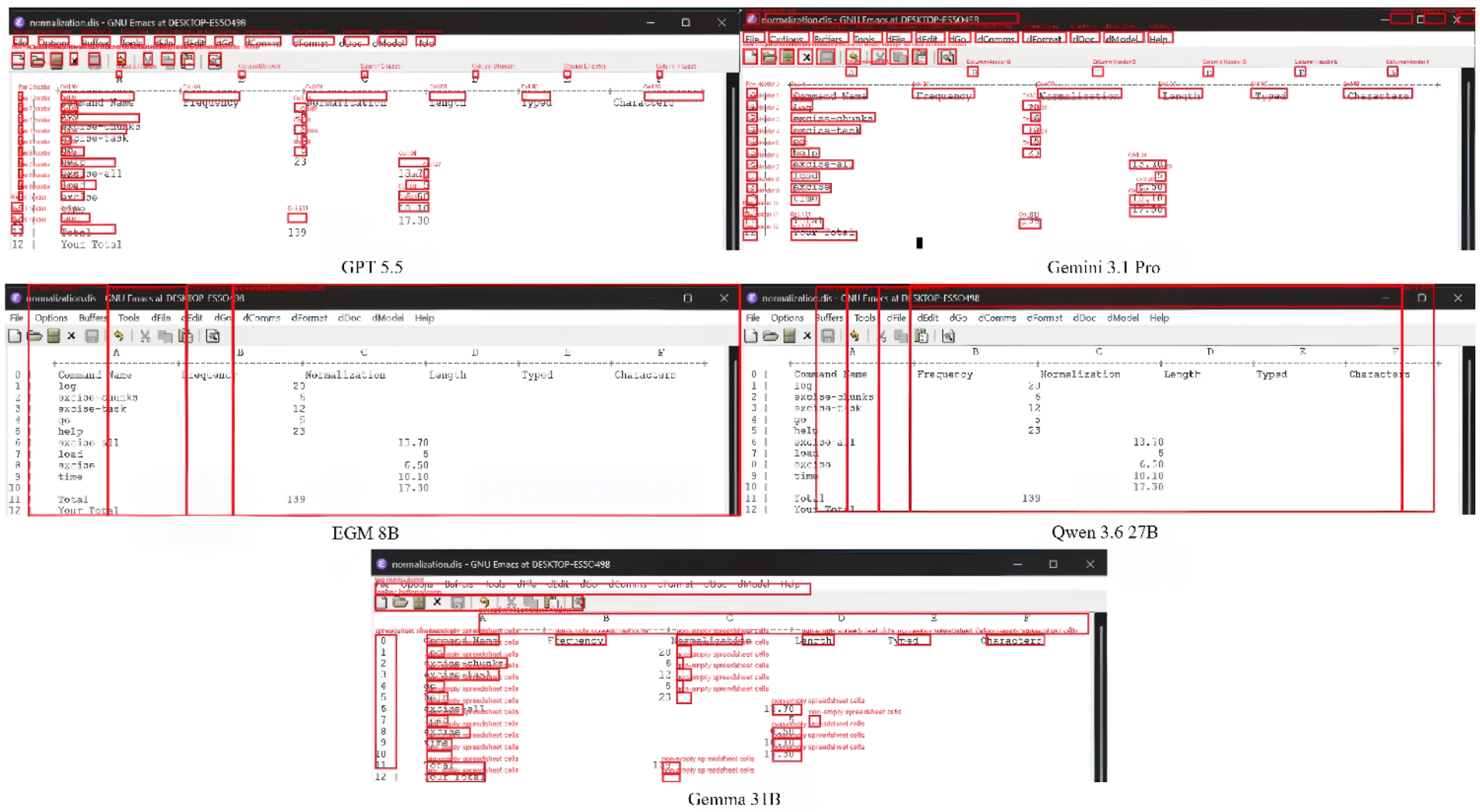

Figure S6: Comparison of VLM-based visual-environment parsing for the same Dismal interface state. GPT-5.5 produced plausible semantic labels for many visible objects, but its bounding-box locations were not sufficiently accurate for direct use as ACT-R visicon entries. Gemini 3.1 Pro (DeepMind, 2026a) produced the most accurate parsing among the tested models in this interface state. The smaller/open-weight models tested here - EGM-8B, Qwen3-VL-30B-A3B (Qwen, 2026), and Gemma 4 31B (DeepMind, 2026b) - did not reliably identify and localize the task-relevant visual objects, although Gemma 4 31B produced the most detailed output among this smaller-model group.

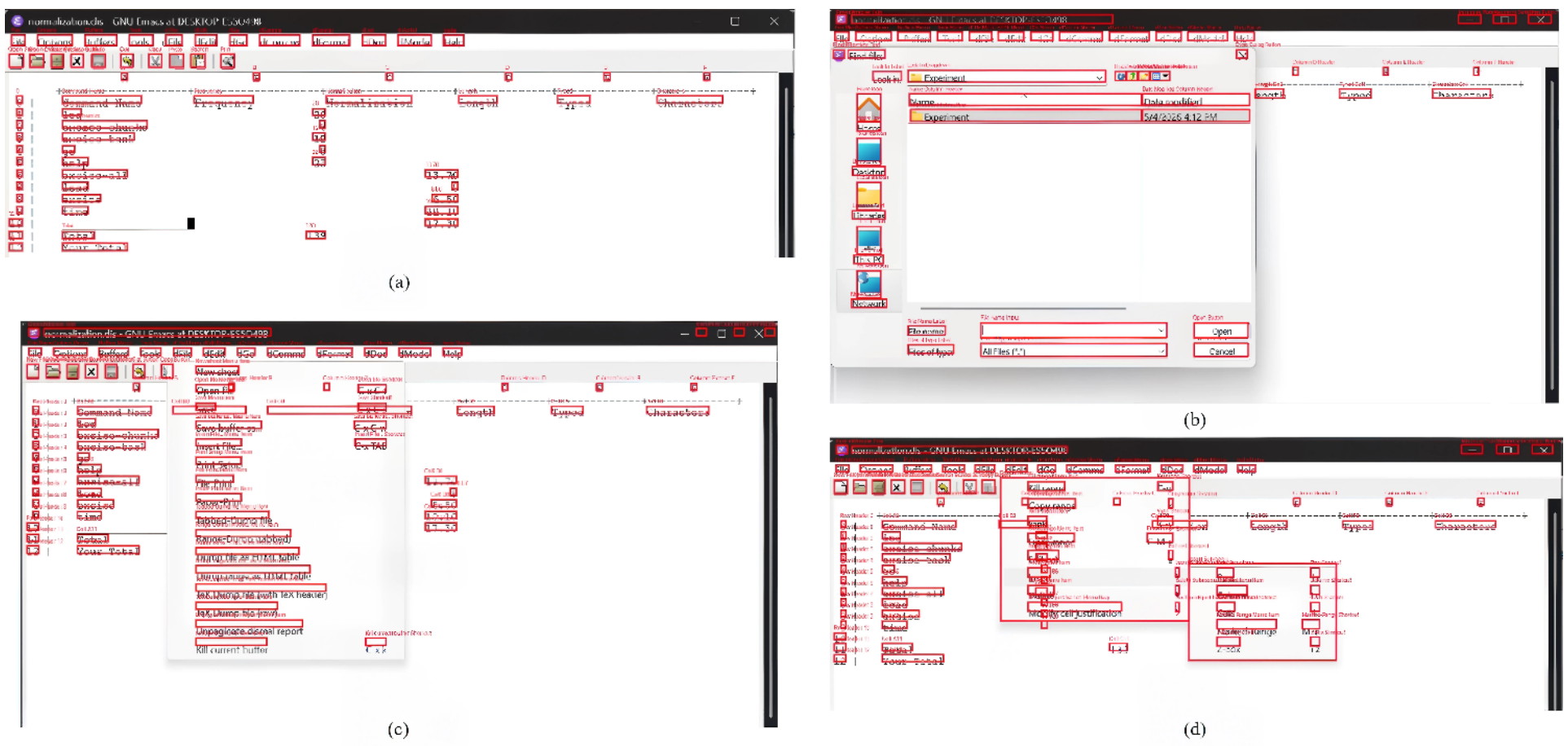

Figure S7: Preliminary VLM-based visual-object extraction across different Dismal interface states using Gemini 3.1 Pro. The model was prompted to identify visible task-relevant interface objects and return labels with bounding boxes. The examples show detections for: (a) the main Dismal spreadsheet workspace, (b) a modal file-opening dialog, (c) an expanded application menu, and (d) an overlapping/cascading menu state. Gemini 3.1 Pro detected many salient objects, including menu labels, toolbar controls, dialog fields, row and column headers, and some spreadsheet entries. However, the output was not fully complete or stable: some small controls were missed, some adjacent objects were merged into larger bounding boxes, and several labels or box locations were imprecise, especially when menus overlapped the spreadsheet.

a parser failure from an interface that genuinely lacks the object. Instability across states is the harder problem, because a backend that is accurate on the workspace view but unreliable on cascading menus is unreliable for the task as a whole.

The useful conclusion is that semantic interpretation and spatial grounding should be separated. General-purpose VLMs were the strongest component we tested for interpreting an unfamiliar layout - saying what an element is - while accessibility metadata and application-specific extractors remain far better for dense object coverage and coordinate precision. For EVisiTor the appropriate design is therefore the hybrid pipeline described in the main text, in which UIA and application-specific extraction supply the visicon geometry and VLMs are reserved for semantic interpretation, subject to the constraint that the model must not gain access to hidden interface state that a human user could not see.

Availability. The parser implementations and run scripts are in EnvironmentParsers/ (OpenAI_VisionParser.py, Gemini_VisionParser.py, UIAGroundedParser.py, uia_grounding_common.py, run_qwen3vl.py, run_gemma4.py, run_egm.py, run_perstep.py, analyze_w6_uia.py, functional_check.py) and in LLMParser.py at the repository root. The annotated Dismal interface states these figures were produced from are in Dismal_annotated/ and Dismal_json/. The exact model identifiers, providers, access dates and endpoints for every system named above are in Supplementary Appendix D.

N.5 References cited only in the Supplementary Material

The following works are cited in this appendix but not in the main article, and are therefore listed here rather than in the main reference list. All other citations in this Supplementary Material appear in the main article's reference list.

DeepMind, G. (2026a, February 19, 2026). Gemini 3.1 Pro - Model Card. Google DeepMind. https://deepmind.google/models/model-cards/gemini-3-1-pro/

DeepMind, G. (2026b, April 2026). Gemma 4. Google. https://huggingface.co/collections/google/gemma-4

https://blog.google/innovation-and-ai/technology/developers-tools/gemma-4/

Qwen, T. (2026). Qwen3-VL-30B-A3B-Instruct [Large Multimodal Model]. Qwen.

https://huggingface.co/Qwen/Qwen3-VL-30B-A3B-Instruct

Yu, W., Yang, Z., Wan, J., Song, S., Tang, J., Cheng, W., Liu, Y., and Bai, X. (2026). Omniparser v2: Structured-points-of-thought for unified visual text parsing and its generality to multimodal large language models. IEEE Transactions on Pattern Analysis and Machine Intelligence.

Supplementary Appendix O. Task 7/9 Sensitivity and Factorial Ablation Diagnostics

O.1 Post hoc Task 7/9 sensitivity analysis

The primary comparison includes all 14 spreadsheet subtasks. Because Tasks 7 and 9 were the only subtasks on which the realistic run distribution was farther from the human distribution, and both contain long repeated-formula sequences, we recomputed the fit after excluding each task separately and both together. These are post hoc diagnostic analyses rather than revised inclusion criteria. Task-level metrics use the same human means and SDs and the same 142-run condition means as the primary analysis. Wilcoxon tests are exact, paired tests of absolute subtask error. Correlation is reported only as a secondary rank-order description because it can remain high despite systematic timing error and is not used as the principal differentiating criterion. Table S14 reports the mean-time fit under each exclusion scope.

Table S14. Sensitivity of mean-time fit to Tasks 7 and 9. Metric cells report realistic / perfect.

Scope	RMSE R/P	SD-norm R/P	MAE R/P
All (n = 14)	17.37 / 29.19	0.657 / 1.240	10.52 / 23.11
Exclude 7 (n = 13)	10.93 / 30.05	0.438 / 1.279	7.35 / 23.83
Exclude 9 (n = 13)	16.49 / 29.50	0.669 / 1.281	9.31 / 22.97
Exclude 7+9 (n = 12)	8.47 / 30.44	0.435 / 1.325	5.77 / 23.74

In the 12-task diagnostic, task-resampling 95% confidence intervals for the realistic-minus-perfect differences were $[-33.03, -9.32]$ s for RMSE, $[-1.08, -0.73]$ for human-SD-normalized RMSE, and $[-28.79, -9.28]$ s for MAE. The realistic model had the smaller absolute error on 12/14, 12/13, 12/13, and 12/12 subtasks in the four scopes; the corresponding exact paired Wilcoxon p values were 0.011, 0.00049, 0.013, and 0.00049. Realistic/perfect correlations for the four scopes were 0.950/0.908, 0.970/0.919, 0.941/0.894, and 0.981/0.949, respectively, and each dependent difference reached significance (Steiger $p = 0.006$, 0.002, 0.008, and 0.038). As in the main text, correlation is reported only as a secondary rank-order description and is not used as the principal differentiating criterion; the inference rests on RMSE, normalized RMSE, MAE, and Wasserstein distance. Table S15 reports the distributional sensitivity under the same exclusion scopes.

Table S15. Distributional sensitivity using Wasserstein-1 distance. Per-subtask cells report realistic / perfect in seconds. Difference intervals and p values come from a 10,000-resample bootstrap of participants and model runs.

Scope	Mean W1 R/P	Difference [95% CI]	p
All (n = 14)	16.45 / 23.44	−6.99 [−11.21, −2.52]	0.002
Excl. 7 (n = 13)	13.55 / 23.64	−10.09 [−13.54, −6.25]	< 0.001
Excl. 9 (n = 13)	14.21 / 22.99	−8.78 [−12.18, −5.18]	< 0.001
Excl. 7+9 (n = 12)	10.87 / 23.18	−12.31 [−14.80, −9.26]	< 0.001

For total completion time, Wasserstein distance was 125.5 s versus 305.6 s in the primary 14-subtask analysis and 83.4 s versus 276.2 s after excluding Tasks 7 and 9 (realistic versus perfect).

The sensitivity analysis localizes the full-sample uncertainty rather than eliminating it by fiat. Tasks 7 and 9 require repeated formula entry, where participants can exhibit within-sequence practice, larger formula chunks, template reuse, memory-based typing, and adaptive checking. Those strategy changes are not represented by the model's fixed tokenization, look-back schedule, and checking policy. The Excel task is used as a case study of the perception-action and instruction capabilities EVisiTor enables, not as a claim that the present model captures all mechanisms of Excel expertise.

O.2 Factorial ablation: mechanism locality and interaction estimates

The task breakdown supports mechanism locality: visual search adds substantial time on the interface-navigation tasks 1, 2, and 11 (23.30 s, 11.20 s, and 28.18 s) and little time on the typing-dominated tasks, whereas batched formula typing and correction checking add substantial time primarily on the formula tasks (Table S16). Task 14 is the fourth interface-navigation subtask but costs only 2.15 s, because it repeats the Save-a-Copy navigation of Task 2 and the model reuses the remembered-location chunks it stored there instead of searching again; its run-to-run variability, however, still comes from visual search, which is why the main text groups Tasks 1, 2, 11, and 14 together when discussing between-run spread. The reported batch is the complete $2 \times 2 \times 2$ factorial, so the interactions between mechanisms are estimated directly

rather than bounded by a residual: the saturated factorial fit for total completion time is reported in the main text (Table 5), with effect coding (−0.5, +0.5) and a stratified bootstrap of runs within each of the eight cells.

Table S16. Descriptive mechanism increments and joint departure from additivity (seconds), computed from the eight-cell factorial batch. Single-mechanism increments are cell mean minus Perfect (000). The residual is Full − Visual-only − Batched-only − Correction-only + 2 × Perfect; under 0/1 coding it equals the sum of the two- and three-way interactions, which the factorial fit shows to be dominated by B × C.

Task	Δ visual	Δ batched	Δ checking	Joint residual
1	23.30	0.00	0.00	+0.00
2	11.20	0.00	2.00	+0.04
3	3.46	43.92	14.09	−7.55
4	0.72	7.53	2.03	−0.90
5	3.46	43.90	13.77	−7.14
6	0.51	7.44	2.81	−1.54
7	5.65	51.91	12.00	−4.12
8	0.56	7.39	2.12	−0.87
9	5.67	39.45	6.94	−0.86
10	0.56	7.29	1.85	−0.83
11	28.18	−0.01	0.01	+0.95
12	0.71	−0.00	2.00	+0.04
13	0.48	−0.02	2.43	+0.12
14	2.15	0.01	5.92	0.00

Summed over all 14 tasks, the joint residual of the four single-mechanism cells was −22.7 s [−27.6, −17.7] (independent-run bootstrap 95% CI, $p < 0.001$). The factorial fit attributes this departure from additivity entirely to the B × C interaction (−23.04 s [−26.49, −19.69], $p < .001$): the error-and-correction package costs about 23 s less when formula typing is token-batched than when it is not, because token boundaries already return attention to the formula source and so catch a slip within one token, keeping the deleted-and-retyped tail short. The interaction is sub-additive, which is the expected shape for two partly redundant mechanisms; it is a smaller increment rather than a cheaper condition, since batched formula typing remains the largest single cost in the model (+197.74 s). The per-task residuals in Table S16 are correspondingly negative and concentrated on the long formula subtasks (Tasks 3, 4, 5, 6, 7, 8, 9, and 10; Holm-adjusted $p \leq .007$ in the subtask-level factorial tests). No interaction involving visual search was significant (V × B +0.99 s [−2.42, +4.30]; V × C −0.53 s [−3.88, +2.89]; V × B × C +0.17 s [−6.64, +6.89]), so the visual-search cost combines additively with the typing-side mechanisms.

Supplementary Appendix P. Detailed Tables Supporting the Main Text

This appendix reports the detailed tables that underlie summary results in the main text. Tables S17 and S18 give the per-subtask completion-time breakdowns for the realistic and perfect models summarized in Table 4 of the main text. Tables S19 and S20 give the human-fit and process measures for the eight ablation cells discussed in the Ablation study section. Table S21 gives the gold decompositions and live execution times for the six novel Windows tasks discussed in the generalization analysis.

Table S17. Realistic model per-subtask completion-time breakdown.

Subtask	Human mean	Human SD	Real. mean	Real. SD	Diff	Abs err.	Diff (SD)
1	25.02	15.44	29.29	10.83	4.27	4.27	0.28
2	42.38	18.38	23.50	3.73	−18.88	18.88	−1.03
3	117.09	35.41	110.87	2.68	−6.22	6.22	−0.18
4	28.87	16.31	20.20	1.16	−8.67	8.67	−0.53
5	111.85	42.32	110.93	2.38	−0.92	0.92	−0.02
6	20.89	7.91	20.32	1.14	−0.57	0.57	−0.07
7	105.02	27.46	156.72	2.74	51.70	51.70	1.88
8	22.25	8.72	19.94	1.28	−2.31	2.31	−0.26
9	108.51	55.99	134.80	2.71	26.29	26.29	0.47
10	22.75	8.98	20.31	1.00	−2.44	2.44	−0.27
11	61.46	28.95	42.99	9.70	−18.47	18.47	−0.64
12	13.81	11.63	9.81	0.86	−4.00	4.00	−0.34
13	13.15	4.28	11.58	0.97	−1.57	1.57	−0.37
14	22.34	7.01	21.37	1.48	−0.97	0.97	−0.14

Table S18. Perfect model per-subtask completion-time breakdown.

Subtask	Human mean	Human SD	Perf. mean	Perf. SD	Diff	Abs err.	Diff (SD)
1	25.02	15.44	5.99	0.35	−19.03	19.03	−1.23
2	42.38	18.38	10.26	0.20	−32.12	32.12	−1.75
3	117.09	35.41	56.95	0.33	−60.14	60.14	−1.70
4	28.87	16.31	10.82	0.12	−18.05	18.05	−1.11
5	111.85	42.32	56.93	0.44	−54.92	54.92	−1.30
6	20.89	7.91	11.10	0.22	−9.79	9.79	−1.24
7	105.02	27.46	91.29	0.41	−13.73	13.73	−0.50
8	22.25	8.72	10.74	0.15	−11.51	11.51	−1.32
9	108.51	55.99	83.59	0.53	−24.92	24.92	−0.45
10	22.75	8.98	11.44	0.28	−11.31	11.31	−1.26
11	61.46	28.95	13.86	0.30	−47.60	47.60	−1.64
12	13.81	11.63	7.07	0.16	−6.74	6.74	−0.58
13	13.15	4.28	8.56	0.20	−4.59	4.59	−1.07
14	22.34	7.01	13.30	0.10	−9.04	9.04	−1.29

Table S19. Human-fit summary by ablation cell (complete 2×2×2 factorial, VBC coding, 142 runs each; human total 715.4 s).

Condition	Total (s)	r	RMSE (s)	SD-norm RMSE	Mean abs err (s)
Perfect (000)	391.9 ± 1.1	0.908	29.19	1.240	23.11
Visual search only (100)	478.5 ± 14.8	0.924	23.68	0.991	17.53
Batched formula typing (010)	600.7 ± 2.0	0.923	20.72	0.987	15.72
Correction checking (001)	459.9 ± 19.6	0.919	24.19	1.010	18.25
Visual + batched (110)	688.2 ± 15.8	0.950	16.04	0.733	11.73
Visual + checking (101)	545.9 ± 23.6	0.944	18.27	0.750	13.22
Batched + checking (011)	645.6 ± 5.9	0.922	21.31	0.892	14.53
Full realistic (111)	732.6 ± 16.2	0.950	17.37	0.657	10.52

Table S20. Process measures by ablation cell (142-run means). Formula IKI is the within-formula-entry inter-key interval matched to the human 0.70 s definition.

Condition	Keys	Attn shifts	Search events	Look-backs	Formula IKI (s)
Perfect (000)	481.0	524.0	0.0	0.0	0.37
Visual search only (100)	481.0	1359.3	292.8	0.0	0.37
Batched formula typing (010)	481.0	852.0	0.0	164.0	0.89
Correction checking (001)	638.2	524.0	0.0	0.0	0.38
Visual + batched (110)	481.0	1696.3	295.8	164.0	0.89
Visual + checking (101)	631.9	1386.7	301.9	0.0	0.38
Batched + checking (011)	529.2	852.0	0.0	164.0	0.88
Full realistic (111)	527.7	1700.9	297.3	164.0	0.88

Table S21. The six novel Windows tasks, their gold decompositions, and live execution times. Human time is the span of the single recorded PSAI demonstration (first to last recorded event); model time is ACT-R simulated time for the completed live run (completion verified by system state).

ID	Task (application)	Subtasks	Actions	Human demo (s)	Model live (s)
W01	Open Notepad via the Run dialog (Win+R)	2	3	10.7	8.2
W02	Rename budget.xlsx to budget_2025.xlsx (File Explorer command bar)	4	8	18.2	19.0
W03	Create folder 'Projects' (Ctrl+Shift+N)	4	7	21.9	12.4
W04	Compress 'Logs' folder (Show more options > Send to > Compressed folder)	4	9	20.3	9.7
W09	Open Calculator via Start-menu search	3	5	12.6	12.5
W10	Find report.docx via File Explorer search	4	7	25.3	14.4
	Mean			18.2	12.7
